# Chemical Recycling of Polystyrene to Valuable Chemicals via Selective Acid-Catalyzed Aerobic Oxidation under Visible Light

**DOI:** 10.1021/jacs.2c01410

**Published:** 2022-03-30

**Authors:** Zhiliang Huang, Muralidharan Shanmugam, Zhao Liu, Adam Brookfield, Elliot L. Bennett, Renpeng Guan, David E. Vega Herrera, Jose A. Lopez-Sanchez, Anna G. Slater, Eric J. L. McInnes, Xiaotian Qi, Jianliang Xiao

**Affiliations:** † Department of Chemistry, University of Liverpool, Liverpool L69 7ZD, U.K.; ‡ Department of Chemistry and Photon Science Institute, The University of Manchester, Manchester M13 9PL, U.K.; § Engineering Research Center of Organosilicon Compounds & Materials, Ministry of Education, College of Chemistry and Molecular Sciences, Wuhan University, Wuhan, Hubei 430072, P. R. China

## Abstract

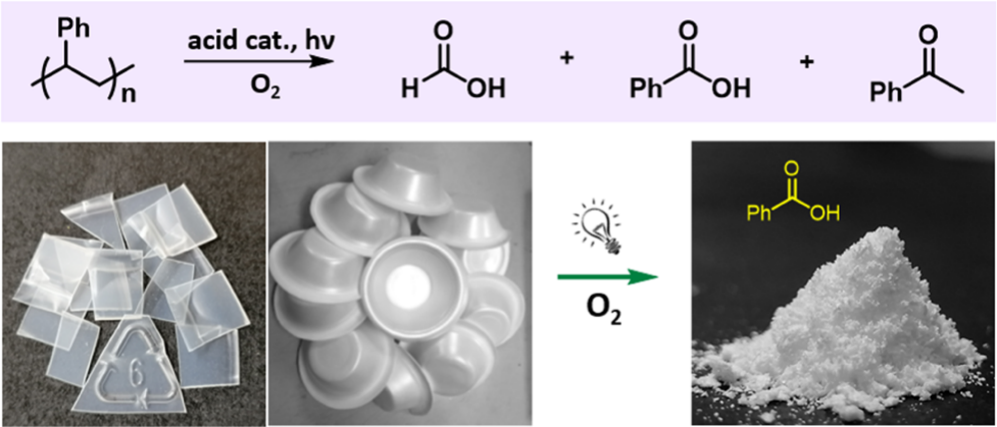

Chemical recycling is one of the most promising technologies that could contribute to circular economy targets by providing solutions to plastic waste; however, it is still at an early stage of development. In this work, we describe the first light-driven, acid-catalyzed protocol for chemical recycling of polystyrene waste to valuable chemicals under 1 bar of O_2_. Requiring no photosensitizers and only mild reaction conditions, the protocol is operationally simple and has also been demonstrated in a flow system. Electron paramagnetic resonance (EPR) investigations and density functional theory (DFT) calculations indicate that singlet oxygen is involved as the reactive oxygen species in this degradation process, which abstracts a hydrogen atom from a tertiary C–H bond, leading to hydroperoxidation and subsequent C–C bond cracking events via a radical process. Notably, our study indicates that an adduct of polystyrene and an acid catalyst might be formed in situ, which could act as a photosensitizer to initiate the formation of singlet oxygen. In addition, the oxidized polystyrene polymer may play a role in the production of singlet oxygen under light.

## Introduction

Since the 1950s, synthetic plastics derived from petroleum have been widely used to improve the quality of people’s lives through clothing, food preservation, and medical applications, among many other domestic and industrial applications. Over the past 70 years, their production has risen sharply, from 1.5 million tonnes in 1950 to 368 million tonnes in 2019,^1,2^ and the production is projected to double again within the next 20 years.^3^ However, once these plastics serve their designated purpose, they pose a serious problem, as most of them are not recycled and do not degrade.^4−6^ Globally, 58% of discarded plastics end up in landfills or are incinerated,^2,4,7,8^ causing severe environmental pollution, including soil contamination, water contamination, air pollution, microplastic pollution, etc.^9−16^ Over the past several decades, some successes have been made to develop closed-loop life cycles for synthetic plastics via collection, separation, and mechanical recycling.^17,18^ Nevertheless, those successes are limited, as the recycled plastics can only be used for downgraded applications^19−21^ and eventually end up in landfills or used for energy recovery after a single recycle. In this regard, chemical recycling is considered one of the most promising solutions to the challenge posed by plastic waste,^22,23^ since this recycling method is able to retain the value of postconsumer polymers by converting them into their original monomers, fuels, or valuable chemicals with potential for upcycled applications.^24−27^ However, to date, chemical recycling is often more energy-intensive and expensive to implement, in comparison to mechanical recycling and incineration.^8,19,28−30^ Therefore, developing more efficient, low-cost methods for the chemical recycling of plastics has become a critical area of research in both chemistry and chemical engineering.^31^ In particular, the search for industrially applicable methods capable of selectively converting plastic wastes to valuable and isolable chemicals with narrow distribution is of utmost interest.

**Scheme 1:**
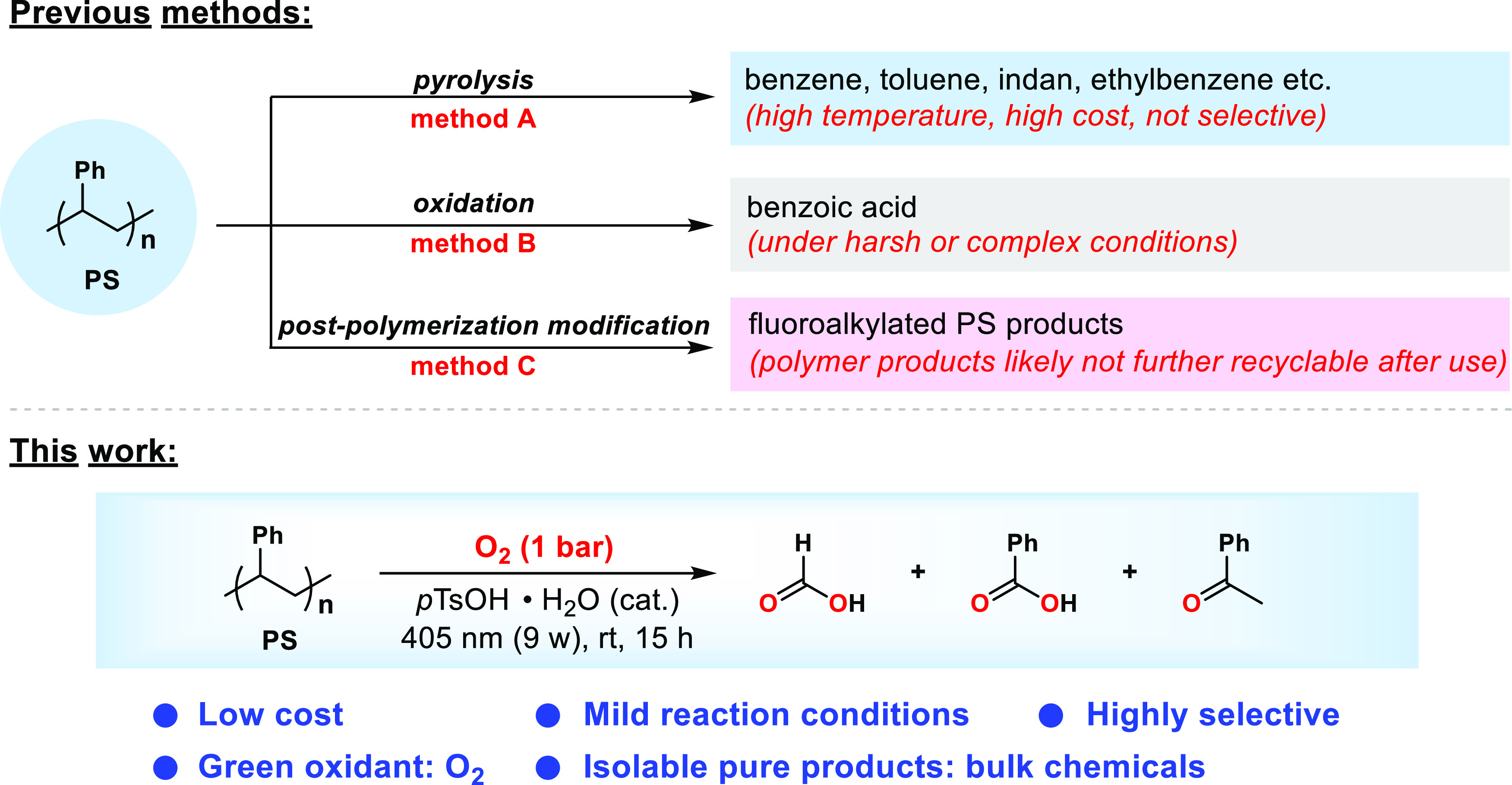
Methods for the Chemical Recycling of PS

Polystyrene (PS), one of the most important materials in the modern plastic industry, has been widely used in our daily life from building materials, electronics, protective packaging, to food containers. Tens of millions of tonnes are produced annually, accounting for about 6% of the current global plastic market share.^32^ Since all atoms of PS are connected by strong C–C and C–H bonds, PS is remarkably inert and difficult to degrade without special treatment. Thermal and catalytic pyrolysis has been developed for chemical recycling of PS under an inert or hydrogen atmosphere (Scheme 1, method A).^33−39^ Generally, this technique requires high temperature (typically >300 °C), appropriate reactors, and catalysts to produce hydrocarbons with a narrow distribution, which lead to high costs.^40−42^ In the last two decades or so, there have been only three examples of catalytic oxidative degradation of PS, reporting aerobic oxidation of PS to form benzoic acid, but the reaction conditions tend to be harsh or complex with a long degradation time,^43−45^ which limits its practical application (Scheme 1, method B). Recently, a new method for postpolymerization modification of PS to the corresponding fluoroalkyl polymers has been developed by Leibfarth and co-workers (Scheme 1, method C).^46^ These modifications could lead to upcycled applications of PS waste, even though the upgraded polymer products are not likely to be further recyclable after use. It is clear that, until now, there appear to be no known efficient methods capable of chemically recycling PS under mild conditions. Herein, following on from our recent studies of dioxygen (O_2_) activation and aerobic oxidation reactions,^47−52^ we report a novel, simple selective degradation method that enables the oxidative cleavage of PS to benzoic acid, formic acid, and acetophenone by singlet oxygen (^1^O_2_) under ambient temperature and pressure with cheap, readily available inorganic or organic acids as a simple catalyst (Scheme 1).

Singlet oxygen is a well-known reactive oxygen species (ROS), which has a relatively high energy of about 94 kJ/mol compared to the ground-state molecular O_2_.^53^ Therefore, ^1^O_2_ is able to initiate low-temperature oxidation of various organic molecules, inspiring a wide array of applications in chemical and biochemical reactions as well as treatment of organic wastes and contaminants.^53−58^ Based on this knowledge, we envisaged that ^1^O_2_ may be able to abstract hydrogen at the weak tertiary benzylic C–H bond in PS and thereby induce the chemical degradation of PS. Indeed, there are some methods in the literature regarding the ^1^O_2_-mediated degradation of PS under light irradiation.^59−62^ However, there are two main disadvantages present in these processes, limiting their practical application. One is that none of these ^1^O_2_-mediated degradation processes could selectively produce pure, valuable chemicals, as they often occur through an uncontrollable radical pathway,^63,64^ while the other is that expensive and/or toxic photosensitizers or initiators are required to produce ^1^O_2_ for the degradation.^65,66^ Hence, developing more practically applicable methods capable of producing ^1^O_2_ and subsequently achieving the degradation of PS in a highly selective manner is urgent. Our findings are presented below.

## Results and Discussion

We commenced our exploration of selective aerobic degradation by employing commercial PS (F.W.: 192,000) as a model substrate. During the search for an able catalyst, we surprisingly found that triflic acid (5 mol %, HOTf) can catalyze the selective degradation of PS using O_2_ (1 bar) as an oxidant under the irradiation of violet-blue light (405 nm), affording isolable formic acid (**1**, 72%), benzoic acid (**2**, 40%), and benzophenone (**3**, 2%) products (Table 1, entry 1). Acid is an essential catalyst for this degradation reaction, without which the chemistry could not proceed (Table 1, entry 2). Further examinations also indicate that light irradiation plays an important role during this aerobic degradation, as no desired products were obtained without irradiation (Table 1, entry 3), and only a small amount of the corresponding products were detected under the irradiation at 475 nm (Table 1, entry 5). The yield achieved under 365 nm irradiation is slightly lower than that of 405 nm (entry 4). We then tested several acid catalysts under irradiation at 405 nm (Table 1, entries 6–9 and 17). The results show that trifluoroacetic acid (CF_3_COOH) and nitric acid (HNO_3_) are inactive, while, in addition to triflic acid, methanesulfonic acid (CH_3_SO_3_H), sulfuric acid (H_2_SO_4_), and *p*-toluenesulfonic acid monohydrate (*p*TsOH·H_2_O) are all good to afford the corresponding products. Several Lewis acids were also examined. Among them, only Sc(OTf)_3_ and La(OTf)_3_ afforded compounds **1**–**3** but in significantly lower yields (entries 27–30). Thereafter, the influence of the quantities of acid catalysts on the yield was investigated. The results showed that 5–10 mol % of an acid catalyst gave better yields (entries 10, 18, and 19), and a larger amount led to a yield drop for **1** (entries 11 and 20). This is likely a result of formic acid decomposition rather than suppression of the reaction by the acid formed (see Section 3.1 in SI). The choice of the reaction solvent revealed a great influence, as no or only trace amounts of target products were obtained when benzene, CH_3_CN, acetone, or EtOAc were employed (entries 12–15), and lower yields were obtained when DCE was used (entry 16). The effect of reaction time, concentration, as well as a solvent on product yields were further examined using *p*TsOH·H_2_O as a catalyst (entries 21–25). The combination of *p*TsOH**·**H_2_O (5 mol %) and benzene/CH_3_CN (1/1, 1 mL) for 15 h was able to afford optimum product yields, while benzene could be replaced by DCE or EtOAc to afford the desired products in slightly lower yields (see SI for more details).^67^ Note that the reaction was much less efficient when carried out in air (entry 26). Compared to the cheap inorganic acid H_2_SO_4_, the industrial-scale and milder *p*TsOH·H_2_O is much easier to handle. Therefore, our subsequent investigation was centered around using *p*TsOH·H_2_O (5 mol %) as a catalyst and O_2_ (1 bar) as an oxidant in benzene/CH_3_CN (1/1, 1 mL) with continuous violet-blue light irradiation at room temperature for 15 h. Note that under such conditions, the main byproduct resulting from the aerobic degradation is oxidized PS of a smaller average molecular weight. An example is seen in the reaction of PS (Mw = 172,389; Mz = 329,367; Mn = 26,934; Mp = 144,697; Mw/Mn = 6.400), in which oxidized PS of a considerably reduced molecular weight was observed (Mw = 41,014; Mz = 92,054; Mn = 15,378; Mp = 16,451; Mw/Mn = 2.667) (see Figures S2 and S3 for the detailed GPC results).

**Table 1:**
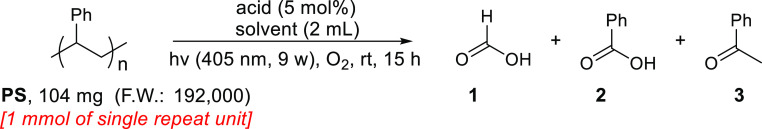

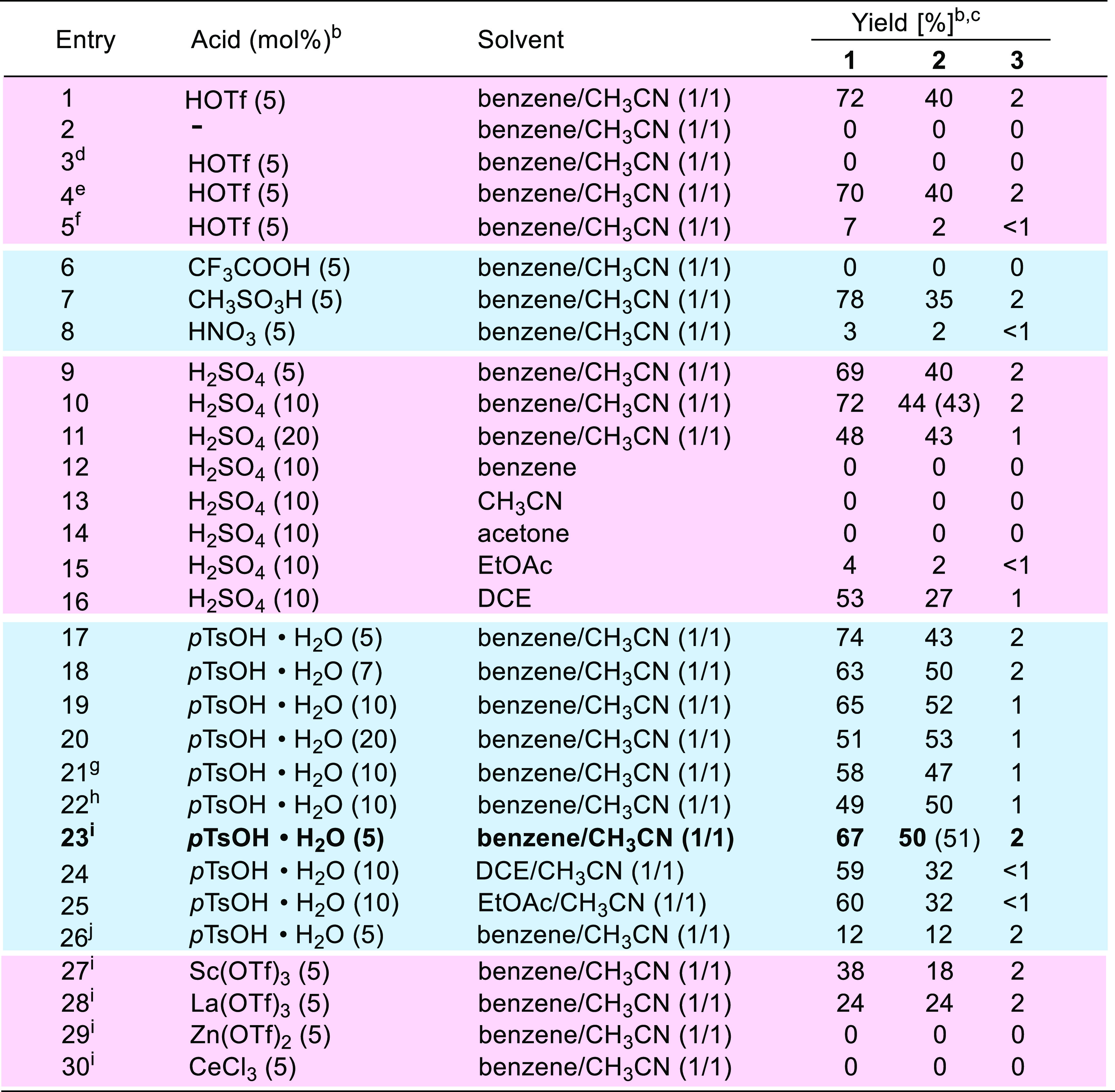
Optimization of the Reaction Conditions^a^^b^^c^^d^^e^^f^^g^^h^^i^^j^

a Reaction was carried out with 104 mg of PS in the presence of an acid catalyst in 2 mL of a solvent under O_2_ (1 bar) and violet-blue light (405 nm, 9 W) for 15 h.

b Catalytic amount of acid and the yield of products are based on the single repeat unit (1 mmol) of polystyrene.

c Yield determined by ^1^H NMR with 1,3,5-trimethoxybenzene as an internal standard; isolated yield in parentheses.

d Without light.

e 365 nm.

f 475 nm.

g 10 h.

h 24 h.

i Benzene/CH_3_CN (1/1, 1 mL).

j Under air.

With our optimized reaction conditions established, the aerobic degradation of commercial, pure PS, and PS waste from our daily life was investigated. As shown in Table 2, all PS materials were readily cleaved by O_2_ to the desired acid products in a highly selective manner. Thus, commercial pure PS of different average molecular weights could be oxidatively cleaved to afford **1** in a 57–67% NMR yield, **2** in a 36–51% isolated yield, and **3** in a 2–5% NMR yield (entries 1–3). Moreover, it seems that PS with a higher molecular weight could give better product yields. Remarkably, PS waste from cup lids, yogurt containers, loose-fill chips, EPS foam, food boxes, as well as laboratory weighing boats are all suitable, producing **1** in a 58–64% NMR yield, **2** in a 38–48% isolated yield, and **3** in a 2–3% NMR yield (entries 4-9). Poly(4-*tert*-butylstyrene) (F.W.: 50,000–100,000) was also tested, which could be selectively degraded to **1** in a 49% NMR yield, 4-*tert*-butylbenzoic acid **4** in a 50% isolated yield, and 4′-*tert*-butylacetophenone **5** in a 3% NMR yield (entry 10).

**Table 2:**
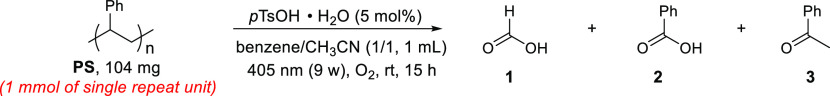

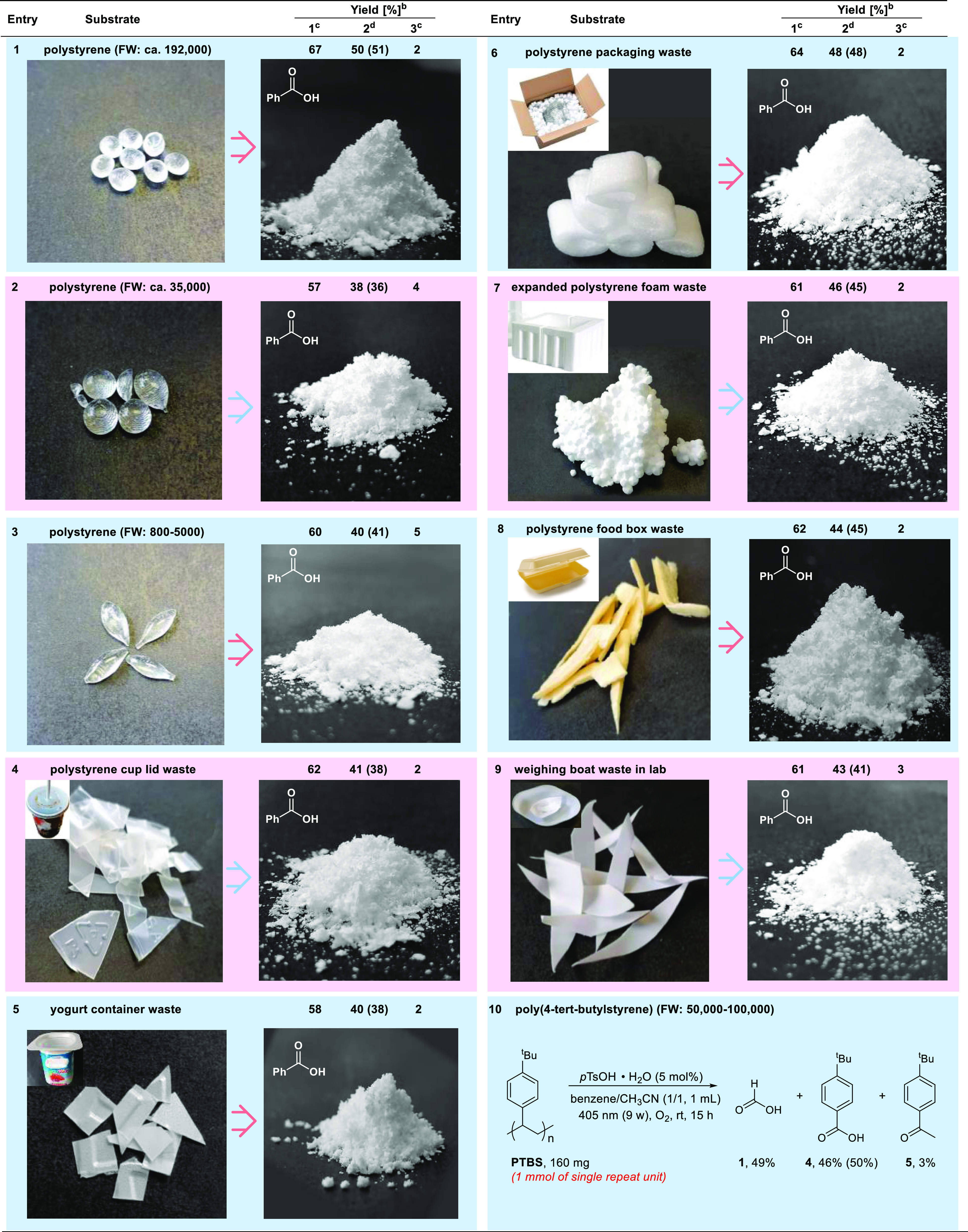
Aerobic Degradation of Commercial Pure PS or PS Waste from Our Daily Life^a^^b^^c^^d^

a Reaction was carried out with PS (104 mg) in the presence of *p*TsOH·H_2_O (9.5 mg) as the catalyst in 1 mL of benzene/CH_3_CN (1/1) under O_2_ (1 bar) and violet-blue light (405 nm, 9 W) for 15 h.

b Catalytic amount of acid and the yield of products are based on the single repeat unit (1 mmol) of PS.

c Yield determined by ^1^H NMR with 1,3,5-trimethoxybenzene as an internal standard.

d Isolated yield in parentheses.

**Scheme 2:**
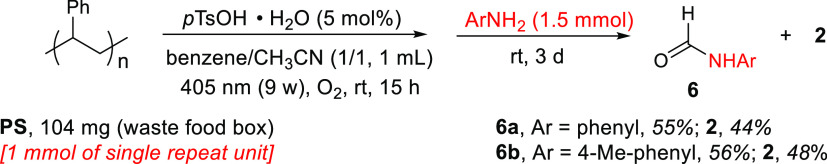
Conversion of the Resulting Formic Acid to Isolable Pure Formanilide

It is worth noting that benzoic acid **2** could be isolated as a pure white crystalline powder from the above-mentioned degradation reactions, as shown in the images in Table 2. Meanwhile, the resulting formic acid could be converted to the isolable pure formanilide by the addition of 1.5 equiv of aniline or *p*-toluidine to the reaction mixture after oxidation. Examples are shown in Scheme 2, where a 55% yield of formanilide **6a** and a 44% yield of **2** were isolated after the addition of aniline, while a 56% yield of 4′-methylformanilide **6b** and a 48% yield of **2** were isolated after the addition of *p*-toluidine.

**Scheme 3:**
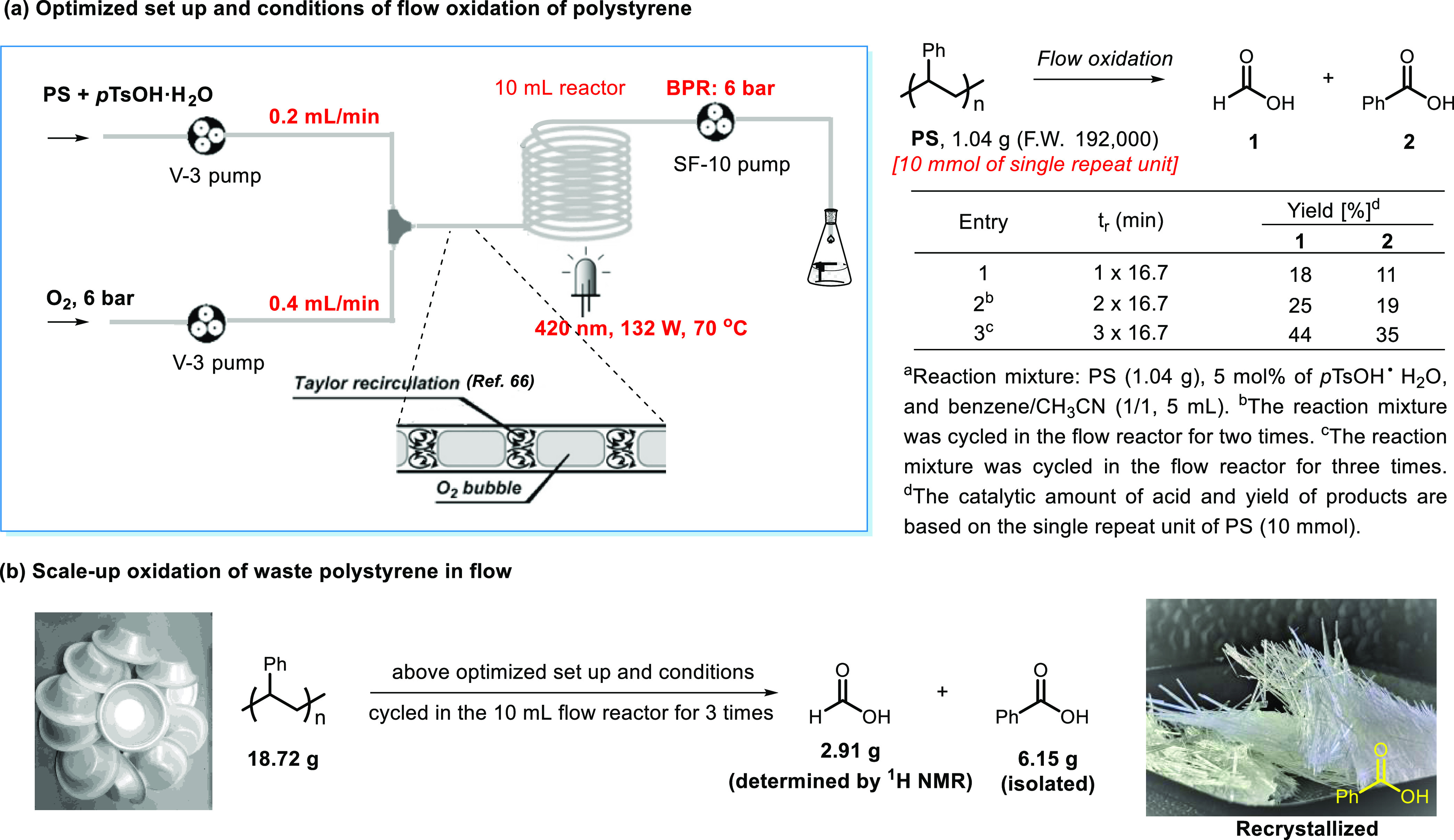
Degradation of PS Enabled by Photocatalysis in Flow: (a) Optimized Setup and Conditions and (b) Gram-Scale Reaction (Note: the E-Series System from Vapourtec Was Used for This Transformation)

The practical applicability of this photo-acid-enabled protocol was further enhanced using the continuous-flow microreactor technology, which has been hailed as an enabling technology to scale-up operationally photochemical transformations.^68,69^ Pleasingly, after a systematic optimization (see the SI for more details), the flow degradation of PS was able to afford the desired products smoothly when a solution of PS and *p*TsOH·H_2_O was mixed with oxygen gas under 6 bar of pressure (BPR) at 70 °C in the presence of violet-blue light (Scheme 3a, entry 1). Note that the 405 nm light is normally more efficient than 420 nm for the aerobic degradation of PS; however, we chose a 420 nm, 132 W high power lamp because it is more easily available and offers acceptable yields (see Table S1 in the SI). Recycling the resulting reaction solution in the flow reactor one or two more times further increased the product yields (Scheme 3a, entries 2 and 3). With the established setup and flow conditions, a scale-up degradation of the waste PS food box was carried out, which produced pure crystalline benzoic acid and formic acid at a gram scale (Scheme 3b).

**Scheme 4:**
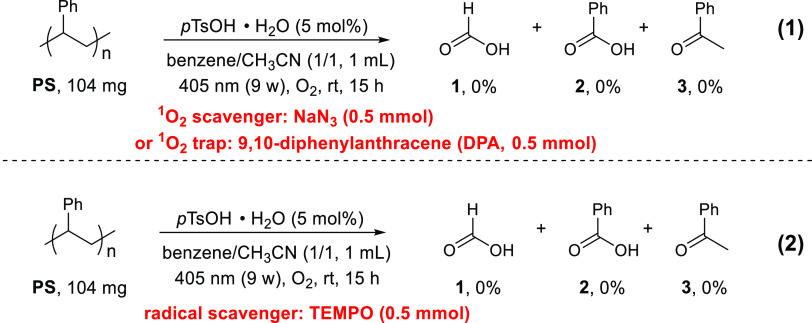
Controlled Experiments in the Presence of an ^1^O_2_ Trap, a Scavenger (1), or a Radical Trap (2)

To shed light on possible reaction pathways, a range of control experiments were carried out. As singlet oxygen (^1^O_2_) was proposed as the ROS for the photodegradation of PS,^63,64^ we thought that it is important to first determine whether ^1^O_2_ indeed plays a role in our degradation system. When PS was subjected to the standard oxidation conditions but in the presence of DPA or NaN_3_ as a ^1^O_2_ trap or scavenger,^70,71^ no conversion or expected degradation products were observed (Scheme 4, eq 1). These observations indicate that ^1^O_2_ is likely the ROS involved in our photo-acid-enabled degradation reaction. Meanwhile, a radical trapping experiment was also conducted. As shown in Scheme 4, eq 2, no PS was converted to the target products under the standard oxidation conditions in the presence of TEMPO, suggesting that the degradation might occur via radical pathways, which is consistent with the reaction mechanism of ^1^O_2_.^63,64,72^

Further evidence on the generation of ^1^O_2_ was obtained by *in situ* electron paramagnetic resonance (EPR) spectroscopy measurements with 4-oxo-TMP (2,2,6,6-tetramethyl-4-piperidone), a well-known ^1^O_2_ trap that generates the nitroxyl radical 4-oxo-TEMPO (2,2,6,6-tetramethyl-4-piperidone-*N*-oxyl). Upon 405 nm irradiation of the reaction solution containing 4-oxo-TMP, the characteristic three-line spectrum is observed immediately but is not observed in an identical experiment under a N_2_ atmosphere (Figure S6A). Furthermore, control experiments show that this signal is enhanced by a known ^1^O_2_ photosensitizer (H_2_TPP; tetraphenyl porphyrin; Figure S6B) and is diminished by a ^1^O_2_ quencher (β-carotene; Figure S6B).

**Figure 1:**
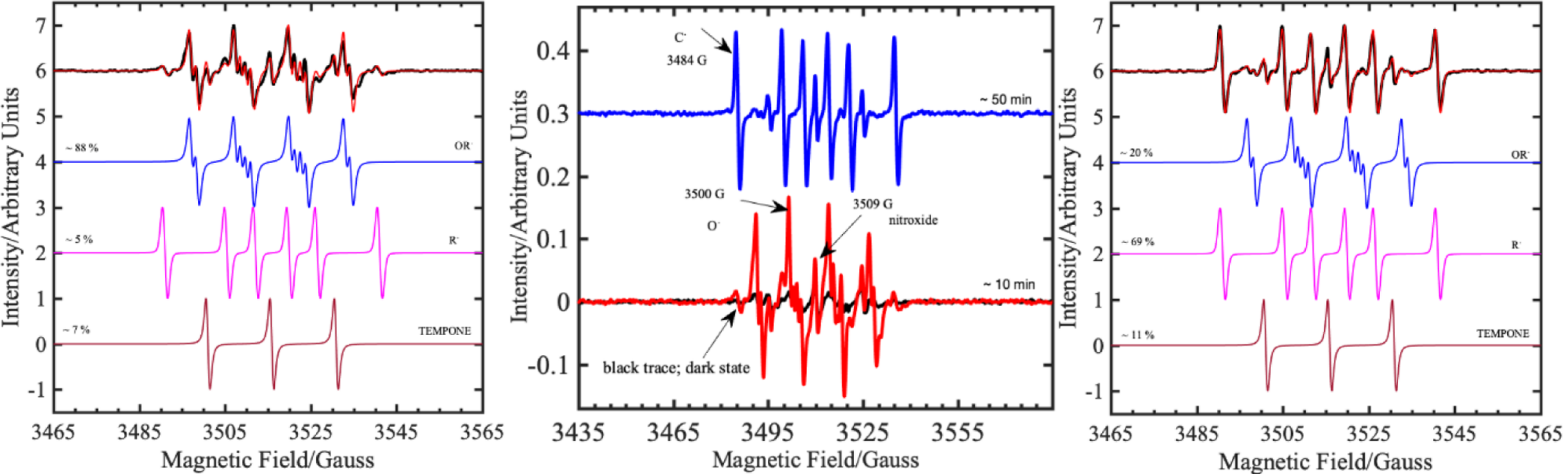
cw EPR spectra from *in situ* irradiation at 405 nm (1 mW LED) of PS and a *p*TsOH·H_2_O solution with 4-oxo-TMP and DMPO spin traps (1:4). Center: experimental spectra measured after 10 min (red) and 50 min (blue) irradiation. Left and right: simulated spectra of three separate components of a nitroxyl [*g* = 2.0056, *a*_iso_(^14^N) = 1. 5 mT], a carbon-centered DMPO adduct [*g* = 2.0055, *a*_iso_(^14^N) = 1.45 mT, *a*_iso_(β*-*^1^H) = 2.11 mT], and an oxygen-centered DMPO adduct [*g* = 2.0057, *a*_iso_(^14^N) = 1.29 mT, *a*_iso_(β*-*^1^H) = 1.03 mT, *a*_iso_(γ*-*^1^H) = 0.13 mT]. Simulations (red) of the experimental spectra (black) after 10 and 50 min (left and right, respectively) are weighted sums of these three components. The magnetic field positions marked in the central panel were used to monitor the separate components as function of time (Figure S10).

The subsequent formation of radicals in the depolymerization reaction was probed by *in situ* EPR reactions also in the presence of the radical trap DMPO (5,5-dimethyl-1-pyrroline *N*-oxide), although this is complicated because DMPO and TEMPO can inhibit reactivity and possibly undergo other reactions under the experimental conditions (see the SI). In experiments with 4-oxo-TMP and DMPO spin traps (1:4, excess DMPO is necessary), we observe complex spectra that can be deconvoluted into four components (identified by the observed hyperfine coupling patterns): a three-line nitroxyl spectrum (presumably 4-oxo-TEMPO and an unidentified DMPO-nitroxide, but not DMPO-X); an oxygen-centered DMPO adduct, most likely DMPO-OR (although DMPO-O_2_H and DMPO-OR have similar spectra, the former is not very persistent, and DMPO-OR forms more readily than DMPO-O_2_R);^73^ and a carbon-centered DMPO-R adduct (Figure 1). The nitroxyl and DMPO-OR adducts are detected initially; then, as they peak, the DMPO-R signal develops (Figure S10). Although we cannot unambiguously identify the radicals, O- and C-centered DMPO-radical adducts are quite distinct,^74^ and control experiments show that the formation of all three types of radical is much quicker in the presence of a PS substrate and an acid catalyst cf. one or neither (Figure S10).

**Figure 2:**
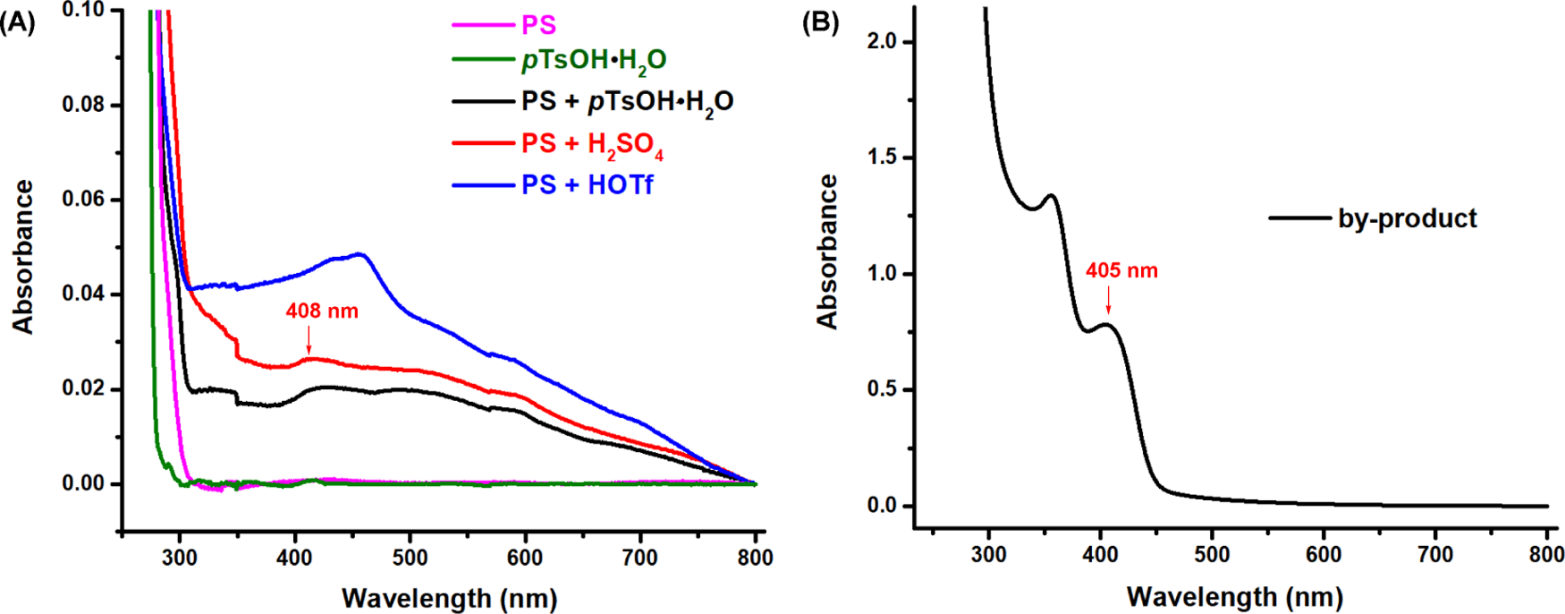
UV–vis spectra of PS, *p*TsOH·H_2_O, the mixture of PS and acid (*p*TsOH·H_2_O, H_2_SO_4_, or HOTf), and the byproduct ([PS]: 10 mM (based on single repeat unit); [acid]: 10 mM; [byproduct]: 1 mg/mL, in DCE).

The formation of ^1^O_2_ under the current conditions raised another question, i.e., how is it formed in the absence of a photosensitizer? To answer the question, UV–vis experiments were performed. As shown in Figure 2A, no absorption band was observed at about 405 nm for both PS and *p*TsOH·H_2_O; however, when they were mixed together, an obvious absorption was detected at around 408 nm. A similar absorption was found in the mixture of PS and H_2_SO_4_ (or HOTf). All of these observations suggested that a [PS---H^+^] adduct resulting from the interaction of PS with the acid might be the photosensitizer that initiates the formation of ^1^O_2_ under the irradiation of violet-blue light.^37^ Meanwhile, the UV–vis spectrum of the isolated byproduct (oxidized PS, Mw: 41,014) was measured, which shows a strong absorption at 405 nm (Figure 2B). This result indicates that during the photochemical degradation, the in situ-formed oxidized PS byproduct could further boost the formation of ^1^O_2_.

**Scheme 5:**
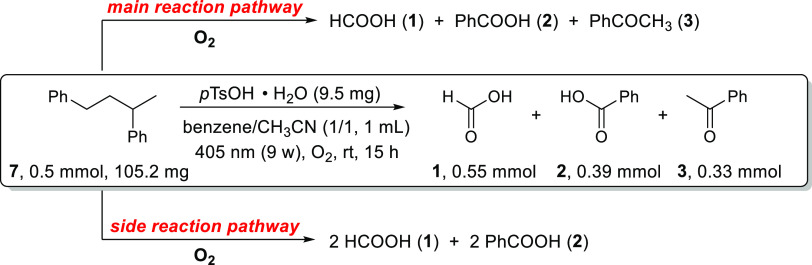
Oxidative Cleavage of 1,3-Diphenylbutane

To simplify the mechanistic investigation and gain further insight into the mechanistic possibilities, a styrene dimer, 1,3-diphenylbutane **7**, was employed to replace PS as a model substrate. As shown in Scheme 5, 0.5 mmol of **7** could be photochemically oxidized to 0.55 mmol of **1**, 0.39 mmol of **2**, and 0.33 mmol of **3** under the standard oxidation conditions. According to this quantitative data, we suspected that there may be at least two pathways for this photochemical oxidation of **7**, i.e., the main reaction pathway that affords 1 equiv of **1**, **2**, and **3**, respectively, and some side reactions, e.g., one that leads to the formation of 2 equiv of **1** and **2**, respectively.

**Scheme 6:**
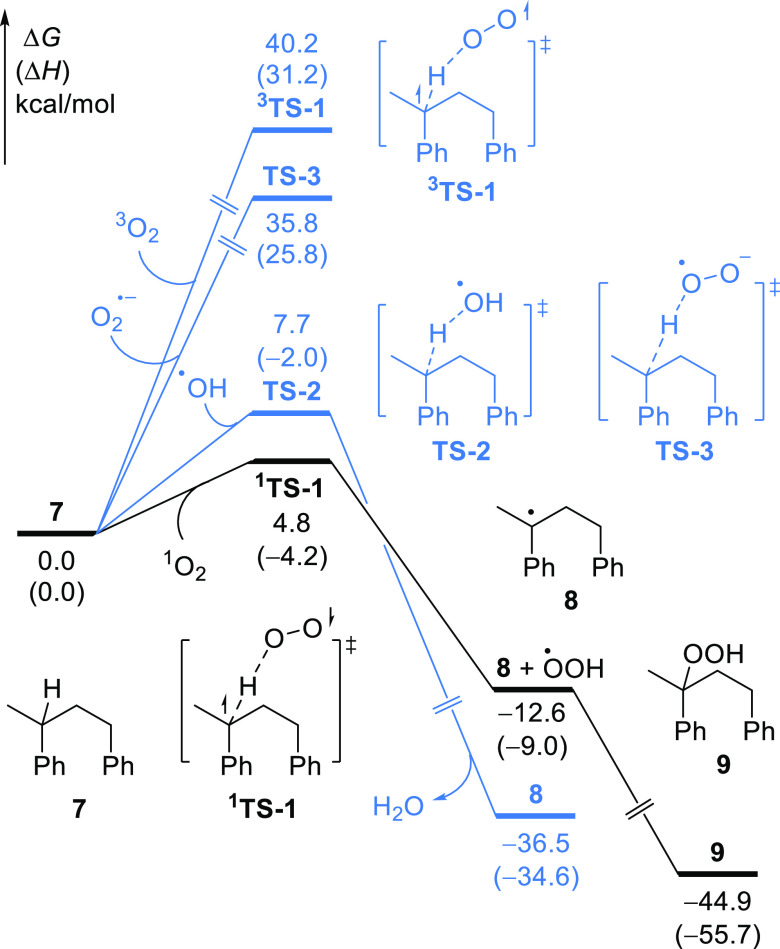
Computational Study of the Hydrogen Atom Transfer (HAT) Reaction of 1,3-Diphenylbutane 7 with Various Oxygen Species All energies were calculated at the B3LYP-D3/6-311+G(d,p)/SMD(acetonitrile^75^)//B3LYP -D3/6-31G(d) level of theory.

Following on from these experimental studies, density functional theory (DFT) calculations were carried out to investigate the oxidative cleavage of **7** with various ROS, including singlet oxygen (^1^O_2_), hydroxyl radical (^•^OH), superoxide ion (O_2_^•–^), and triplet oxygen (^3^O_2_). These ROS could initiate the oxidation of **7** by abstracting the hydrogen atom from the tertiary C–H bond and forming a stable benzyl radical intermediate **8**. As shown in Scheme 6, the ^1^O_2_ involved hydrogen atom transfer (HAT) through the transition state ^**1**^**TS-1** has the lowest energy barrier (Δ*G*^‡^ = 4.8 kcal/mol), which indicates that ^1^O_2_ is the most likely ROS to initial PS degradation. This computational result is consistent with the ^1^O_2_ quenching experiments as well as EPR investigations that support ^1^O_2_ to be the real ROS for the degradation of PS.

**Scheme 7:**
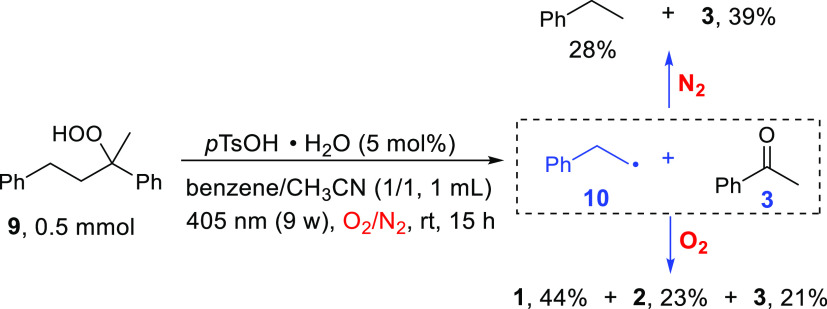
Decomposition of Peroxide 9 under N_2_ or O_2_

Subsequently, the rebound of a hydroperoxyl radical (^•^OOH) generates the peroxide compound **9** irreversibly (Scheme 6). The formation of **9** is exergonic by 44.9 kcal/mol with respect to **7**, which indicates that this stable intermediate is most likely an active intermediate for the following C–C bond cleavage.^76^ Indeed, this speculation was then verified by experimental studies. As shown in Scheme 7, when **9** was subjected to the standard conditions but in the absence of O_2_, ethylbenzene (resulting from 2-phenylethyl radical **10**) and acetophenone were obtained as the main products. Meanwhile, formic acid, benzoic acid, and acetophenone were also observed when the decomposition of **9** was performed under O_2_. In a recent study,^1^O_2_ has been shown to easily insert into α-ethereal C–H bonds, forming hydroperoxides.^56^

**Scheme 8:**
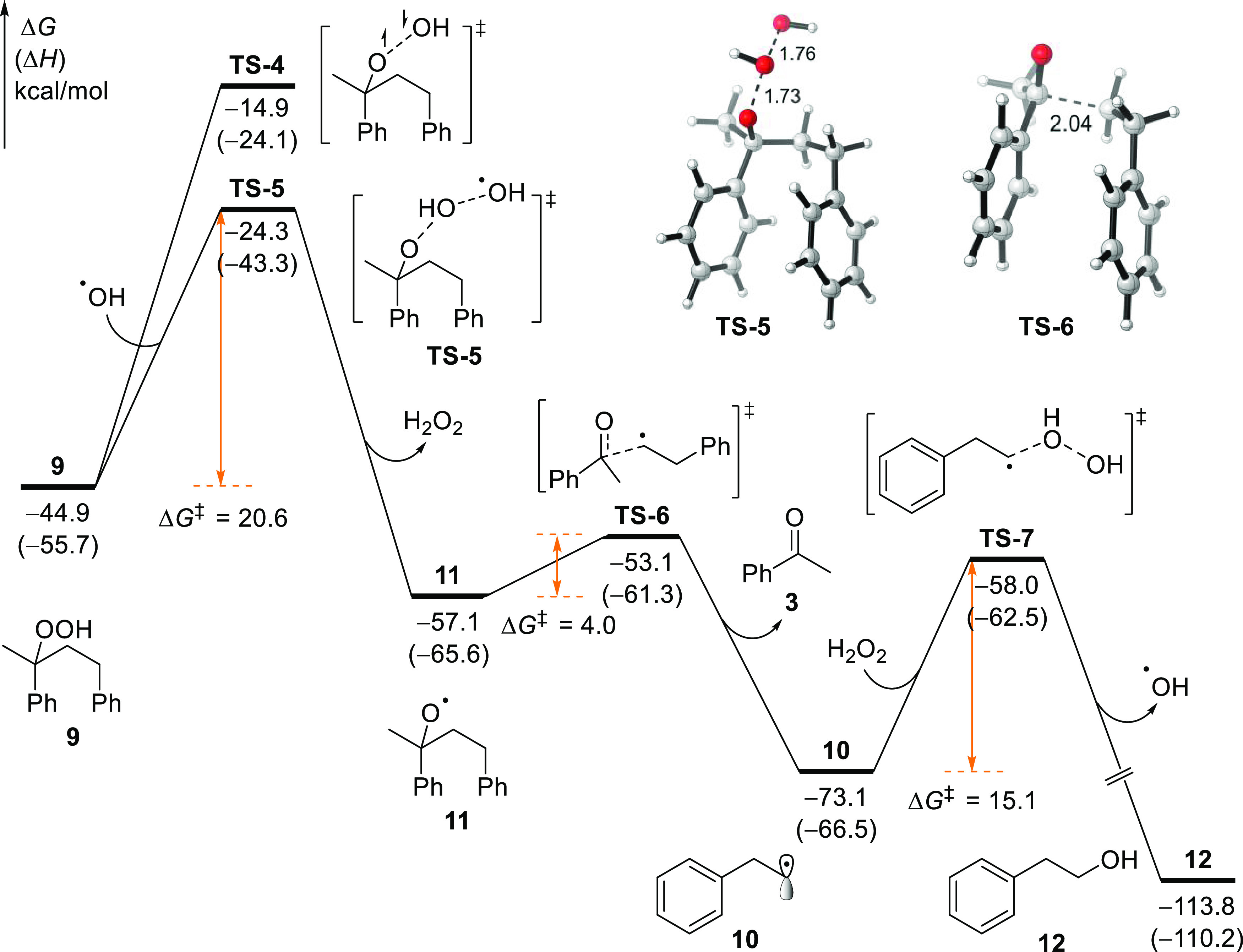
Computational Study of the Decomposition of Peroxide 9 All energies were calculated at the B3LYP-D3/6-311+G(d,p)/SMD(acetonitrile)//B3LYP-D3/6-31G(d) level of theory.

Computational studies of the decomposition of **9** suggest that the homolytic cleavage of the O–O bond through an open-shell singlet transition state **TS-4** appears less likely, requiring an activation free energy of 30.0 kcal/mol (Scheme 8). Surprisingly somehow, the radical substitution pathway with ^•^OH through a doublet transition state **TS-5** entails a significantly lower energy barrier (Δ*G*^‡^ = 20.6 kcal/mol). This step irreversibly generates a key O-centered radical **11**, which can undergo the β-scission facilely (via **TS-6**, Δ*G*^‡^ = 4.0 kcal/mol), leading to the C–C bond cleavage. The formation of acetophenone and alkyl radical **10** is also highly exergonic. The following radical substitution between alkyl radical **10** and hydrogen peroxide gives rise to phenethyl alcohol **12** and regenerates the hydroxyl radical (^•^OH), thereby completing the catalytic cycle. Because phenethyl alcohol **12** also contains the benzyl C–H bond, analogous pathways that consist of HAT, radical rebound, radical substitution, and β-scission of an O-centered radical will result in the decomposition of **12** as well. The generated benzaldehyde and alkyl radical can be finally oxidized to benzoic acid **2** and formic acid **1** under the standard oxidation conditions.

## Conclusions

In summary, a novel photo-acid-enabled protocol has been established for the selective degradation of polystyrene wastes by molecular oxygen for the first time. Featuring photosensitizer-free and mild reaction conditions, the protocol is operationally simple for the chemical recycling of polystyrene waste to valuable chemicals, such as formic acid, benzoic acid, and acetophenone. Flow degradation of polystyrene has also been demonstrated, providing support toward its potential application in industry. Mechanistic investigations indicate that singlet oxygen is formed as the key ROS in the degradation process. Notably, a possible [polystyrene---acid] adduct plays a vital role in the formation of ^1^O_2_ under violet-blue light, the concentration of which is likely to be boosted by the *in situ*-formed oxidized polystyrene polymer acting as a photosensitizer. These findings may open new photosensitizer-free pathways that were previously considered impossible for the aerobic degradation of polystyrene or other polymers featuring weak C–H bonds. DFT calculations suggest that the ^1^O_2_-mediated selective C–H bond hydroperoxidation is the key process for the subsequent C–C bond cracking of polystyrene, although many radical pathways may well follow. Spin-trapping EPR experiments support the involvement of O- and C-centered radicals in this degradation process. Last but not least, this type of chemical recycling can result in the displacement of fossil carbon-based feedstocks while incentivizing better management of polystyrene waste by recovering a considerable material value that can be recirculated into the global economy.

## Supporting Information Available

Experimental details and procedures, optimization studies, mechanistic experiments, and spectral data for all compounds (PDF)
