# Supplementary material for: Chemical Recycling of Polystyrene to Valuable Chemicals via Selective Acid-Catalyzed Aerobic Oxidation under Visible Light
Source: J Am Chem Soc. 2022 Mar 30;144(14):6532–42. doi: 10.1021/jacs.2c01410 (PMC9011358; doi:10.1021/jacs.2c01410)
Supplement: Supplementary file 1 — ja2c01410_si_001.pdf [file ja2c01410_si_001.pdf]

Supporting Information

Chemical Recycling of Polystyrene to Valuable Chemicals via Selective Acid-Catalyzed Aerobic Oxidation under Visible Light

Zhiliang Huang,[1] Muralidharan Shanmugam,[2] Zhao Liu,[3] Adam Brookfield,[2] Elliot L. Bennett,[1] Renpeng Guan,[1] David E. Vega Herrera,[1] Jose A. Lopez-Sanchez,[1] Anna G. Slater,[1] Eric J. L. McInnes,[2,*] Xiaotian Qi,[3,*] and Jianliang Xiao[1,*]

[1] Department of Chemistry, University of Liverpool, Liverpool L69 7ZD, U.K

[2] Department of Chemistry and Photon Science Institute, The University of Manchester, Manchester M13 9PL, U.K

[3] Engineering Research Center of Organosilicon Compounds & Materials, Ministry of Education, College of Chemistry and Molecular Sciences, Wuhan University, Wuhan, Hubei 430072, PR China

E-mail: jxiao@liverpool.ac.uk, qi7xiaotian@whu.edu.cn, eric.mcinnes@manchester.ac.uk

Contents

1. General information 2

2. Information for the photoreactors 2

3. General procedure for the aerobic degradation of PS 3

4. The aerobic degradation of PS in flow 9

5. Mechanistic investigation 12

    5.1 Control experiments 12

    5.2 UV-Vis experiments 12

    5.3 EPR experiments 13

    5.4 DFT calculations 18

6. Analytical data of products 76

7. NMR spectra of products 77

8. References 81

1. General information

All manipulations were carried out using standard Schlenk techniques. All glassware was oven dried at 120 ^{o}C for more than 1 hour prior to use. Polystyrene (F.W. 192,000), polystyrene (F.W. 35,000), polystyrene (F.W. 800-5000), poly(4-*tert*-butylstyrene) (F.W. 50,000-100,000), triflic acid (HOTf), trifluoroacetic acid (CF_3COOH), nitric acid (HNO_3), methanesulfonic acid (CH_3SO_3H), *p*-toluenesulfonic acid monohydrate (*p*TsOH•H_2O), sulfuric acid (H_2SO_4), benzene, acetonitrile, acetone, ethyl acetate, 1,2-dichloroethane, aniline, *p*-toluidine, sodium azide, 5,5-Dimethyl-1-pyrroline *N*-oxide (DMPO), (2,2,6,6-Tetramethylpiperidin-1-yl)oxyl (TEMPO), and 1,3-diphenylbutane were purchased from commercial suppliers and used without further purification. Waste polystyrene materials were collected from daily life and used without further purification or after simply washing with water. (3-Hydroperoxybutane-1,3-diyl)dibenzene was synthesized according to the literature.[1] Unless otherwise noted, analytical grade solvents and commercially available reagents were used as received. Analytical thin-layer chromatography (TLC) was conducted with TLC Silica gel 60 F254 (Merck) and plates were revealed under UV irradiation. Flash column chromatography was performed using Aldrich Silica Gel 60 and columns were packed according to the dry method and equilibrated with the appropriate eluent prior to use. HPLC grade solvents were used and the solvent mixtures used as eluent are understood as volume/volume. The known compounds were characterized by ^{1}H NMR and ^{13}C NMR. The ^{1}H and ^{13}C NMR spectra were recorded on a Bruker Advance 400 NMR spectrometer at 400 MHz (^{1}H NMR) and 101 MHz (^{13}C NMR). The chemical shifts (δ) were given in part per million relative to $CDCl_3$ (7.26 ppm for ^{1}H and 77.00 ppm for ^{13}C). UV-Vis spectra were measured on an Agilent Cary 5000 at the Materials Innovation Factory, University of Liverpool.

2. Information for the photoreactors

Blue LEDs (470 nm): 2.95 V Blue LED SMD, Lumileds LUXEON Rebel LXML-PB01-0040; dominant wavelength or peak wavelength (minimum: 460 nm, typical: 470 nm, maximum: 485 nm); typical spectral half-width (20 nm); typical temperature coefficient of dominant or peak wavelength (0.05 nm/^{o}C); typical total included angle (160°); typical view angle (125°). Each hole on the photoreactor was fixed with three LEDs, with a total power of 9 W for each reaction tube.

UV LEDs (365 nm): 3.0 V UV LED SMD, LUMINUS SST-10-UV-E365-00; dominant wavelength or peak wavelength (minimum: 365 nm, typical: 365 nm, maximum: 370 nm); typical spectral half-width (10 nm); typical total included angle (160°); typical view angle (130°). Each hole on the photoreactor is fitted with three LEDs, giving a total power for each reaction tube as 9 W.

Violet-blue LEDs (405 nm): 3.0 V Violet-Blue LED SMD, LUMINUS SST-10-UV-F405-00; dominant wavelength or peak wavelength (minimum: 405 nm, typical: 405 nm, maximum: 410 nm); typical spectral half-width (10 nm); typical total included angle (160°); typical view angle (130°). Each hole on the photoreactor is fitted with three LEDs, giving a total power for each reaction tube as 9 W.

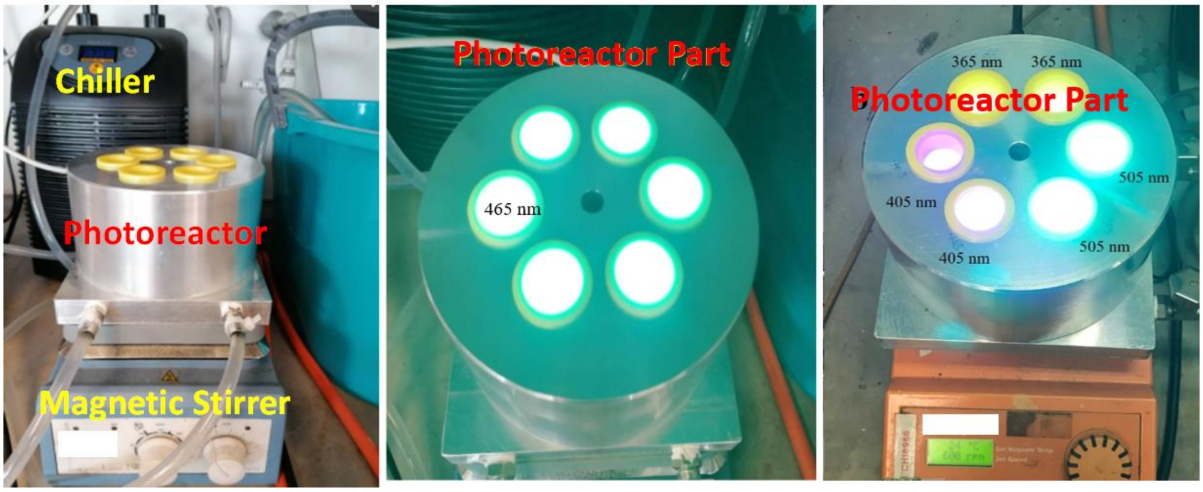

3. General procedure for the aerobic degradation of PS

3.1 The aerobic degradation of PS under various conditions

To an oven dried Schlenk tube, 104 mg of commercial PS (F.W. = 192,000), which contains 1 mmol of the single repeat unit, was added. Then the reaction tube was vacuumed and purged with oxygen via an oxygen balloon. Solvent (1 or 2 mL) was injected through a syringe. Finally, after the addition of an acid catalyst (5-20 mol%), the reaction tube was allowed to stir at room temperature under 405 nm, 365 nm, or 470 nm for 15 h. After the completion of the reaction, 1,3,5-trimethoxybenzene (50 mg) was added as internal standard, and 0.1 mL of the resulting mixture was taken out to dissolve in CD_3Cl (0.4 mL) for ^1H NMR yield analysis. Note: The catalytic amount of acid and yield of products are based on the single repeat unit of polystyrene (1 mmol).

Table S1. Optimization of the reaction conditions[a]

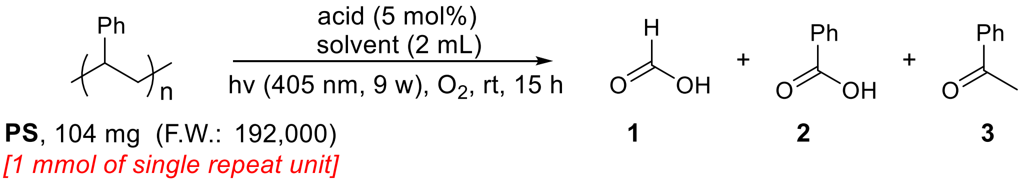

Entry	Acid (mol%)[b]	Solvent	Yield [%][b,c]		
			**1**	**2**	**3**
1	HOTf (5)	benzene/CH_3CN (1/1)	72	40	2
2	-	benzene/CH_3CN (1/1)	0	0	0
3[d]	HOTf (5)	benzene/CH_3CN (1/1)	0	0	0
4[e]	HOTf (5)	benzene/CH_3CN (1/1)	70	40	2
5[f]	HOTf (5)	benzene/CH_3CN (1/1)	7	2	<1
6	CF_3COOH (5)	benzene/CH_3CN (1/1)	0	0	0
7	CH_3SO_3H (5)	benzene/CH_3CN (1/1)	78	35	2
8	HNO_3 (5)	benzene/CH_3CN (1/1)	3	2	<1
9	H_2SO_4 (5)	benzene/CH_3CN (1/1)	69	40	2
10	H_2SO_4 (10)	benzene/CH_3CN (1/1)	72	44 (43)	2
11	H_2SO_4 (20)	benzene/CH_3CN (1/1)	48	43	1
12	H_2SO_4 (10)	benzene	0	0	0
13	H_2SO_4 (10)	CH_3CN	0	0	0
14	H_2SO_4 (10)	acetone	0	0	0
15	H_2SO_4 (10)	EtOAc	4	2	<1
16	H_2SO_4 (10)	DCE	53	27	1
17	*p*TsOH • H_2O (5)	benzene/CH_3CN (1/1)	74	43	2
18	*p*TsOH • H_2O (7)	benzene/CH_3CN (1/1)	63	50	2
19	*p*TsOH • H_2O (10)	benzene/CH_3CN (1/1)	65	52	1
20	*p*TsOH • H_2O (20)	benzene/CH_3CN (1/1)	51	53	1
21[g]	*p*TsOH • H_2O (10)	benzene/CH_3CN (1/1)	58	47	1
22[h]	*p*TsOH • H_2O (10)	benzene/CH_3CN (1/1)	49	50	1
**23**[i]	***p*TsOH • H_2O (5)**	**benzene/CH_3CN (1/1)**	**67**	**50** (51)	**2**
24	*p*TsOH • H_2O (10)	DCE/CH_3CN (1/1)	59	32	<1
25	*p*TsOH • H_2O (10)	EtOAc/CH_3CN (1/1)	60	32	<1
26[j]	*p*TsOH • H_2O (5)	benzene/CH_3CN (1/1)	12	12	2
27	*p*TsOH • H_2O (5)	benzene/EtOAc (1/1)	14	18	3
28	*p*TsOH • H_2O (5)	benzene/acetone (1/1)	21	12	4
29	*p*TsOH • H_2O (5)	benzene/DCE (1/1)	23	11	2
30[i]	$Sc(OTf)_3$ (5)	benzene/CH_3CN (1/1)	38	18	2
31[i]	$La(OTf)_3$ (5)	benzene/CH_3CN (1/1)	24	24	2
32[i]	$Zn(OTf)_2$ (5)	benzene/CH_3CN (1/1)	0	0	0
33[i]	$CeCl_3$ (5)	benzene/CH_3CN (1/1)	0	0	0
34	TFA (5)	benzene/CH_3CN (1/1)	0	0	0
35	TFA (100)	benzene/CH_3CN (1/1)	62	43	3

[a] The reaction was carried out with 104 mg of PS in the presence of an acid catalyst in 2 mL of solvent under O_2 (1 bar) and violet-blue light (405 nm, 9 W) for 15 h; [b] The catalytic amount of acid and yield of products are based on the single repeat unit (1 mmol) of polystyrene; [c] Yield determined by ^1H NMR with 1,3,5-trimethoxybenzene as internal standard; isolated yield in parentheses; [d] Without light; [e] 365 nm; [f] 475 nm; [g] 10 h; [h] 24 h; [i] Benzene/CH_3CN (1/1, 1 mL); [j] Under air.

Note: Compared to entry 17, entries 18-20 (Table 1, manuscript) showed a decrease in yield, but only for the yield of formic acid; actually, an increase was observed for the yield of benzoic acid. The decrease of the yield of formic acid might be caused by its decomposition under a strongly acidic condition, rather than the accumulation of protons. Formic acid is known to decompose in contact with strong acids (https://webwiser.nlm.nih.gov/substance?substanceId=486&identifier=Formic%20Acid&identifierType=name&menuItemId=31&catId=65).

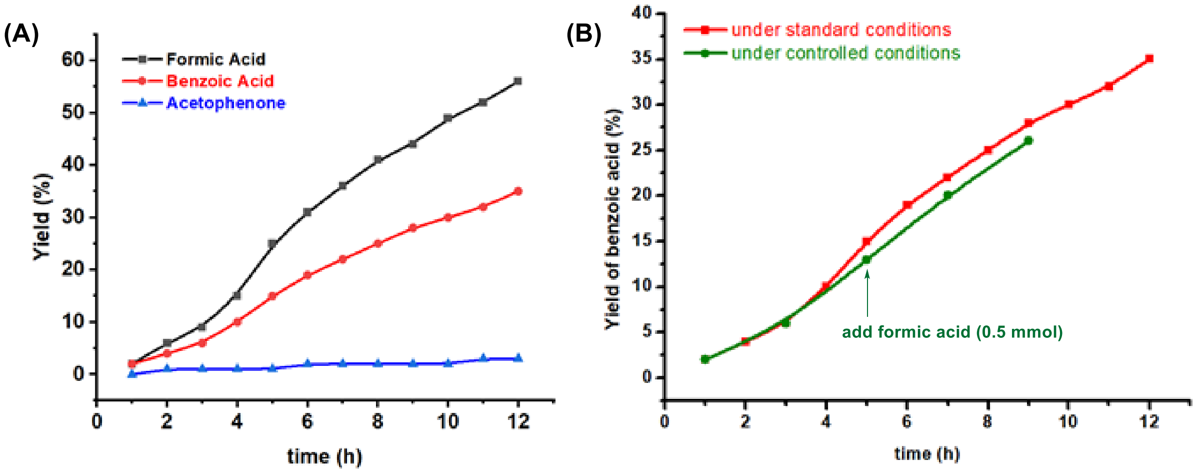

Figure S1. Kinetic behavior of *p*-TsOH•H_2O-catalyzed aerobic degradation of PS under standard conditions (A) and under controlled conditions (B), in which 0.5 mmol of formic acid was added after 5 h reaction under the standard conditions.

To examine whether the acid produced during the acid-catalyzed aerobic degradation would affect the reaction, kinetic studies were performed. As shown in Figure S1(A), there appears to be an induction period in the first 4 hours, which might be caused by the formation of oxidized PS photosensitizer. Thereafter, the formation of formic acid and benzoic acid is a steadily increasing process. The kinetic behavior was also examined by adding 0.5 mmol of formic acid 5 h after the reaction had started. As can be seen in Figure S1(B), no significant change of reaction rate was detected. These results suggest that the excess proton does not suppress the reaction.

The view that a high proton concentration does not suppress the reaction is also supported by additional experiments. As shown in entries 34 and 35 (Table S1), a stoichiometric amount of trifluoroacetic acid (TFA) was able to promote the aerobic degradation of PS to the desired

products in good yields, but a catalytic amount did not afford any products. These results indicate that a high proton concentration is beneficial for the degradation process.

3.2 Standard procedure for aerobic degradation of commercial PS or PS waste

To an oven dried Schlenk tube, 104 mg of commercial PS or PS waste (used after simple washing with water and ethanol), which contains 1 mmol of the single repeat unit, was added. Then the reaction tube was vacuumed and purged with oxygen via an oxygen balloon. Benzene (0.5 mL) and CH_3CN (0.5 mL) were injected through a syringe. Finally, after the addition of *p*TsOH•H_2O (9.5 mg, 5 mol%), the reaction tube was allowed to stir at room temperature under 405 nm for 15 h. After the completion of the reaction, 1,3,5-trimethoxybenzene (50 mg) was added as internal standard, and 0.1 mL of the resulting mixture was taken out to dissolve in CD_3Cl (0.4 mL) for ^1H NMR yield analysis. Note: The catalytic amount of acid and yield of products are based on the single repeat unit of polystyrene (1 mmol). The pure benzoic acid was obtained by flash chromatography on silica gel with hexane/ethyl acetate (4/1).

3.3 Standard procedure for the conversion of the resulting formic acid after degradation to isolable pure formanilides

To an oven dried Schlenk tube, 104 mg of PS waste food box (used after simple washing with water and ethanol), which contains 1 mmol of the single repeat unit, was added. Then the reaction tube was vacuumed and purged with oxygen via an oxygen balloon. Benzene (0.5 mL) and CH_3CN (0.5 mL) were injected through a syringe. Finally, after the addition of *p*TsOH•H_2O (9.5 mg, 5 mol%), the reaction tube was allowed to stir at room temperature under 405 nm for 15 h. After the completion of the reaction, aniline or *p*-toluidine (1.5 mmol) was added, and the resulting mixture was allowed to stir for 3 days in the dark at room temperature. Then, 1,3,5-trimethoxybenzene (50 mg) was added as internal standard, and 0.1 mL of the resulting mixture was taken out to dissolve in CD_3Cl (0.4 mL) for ^1H NMR yield analysis. Note: The catalytic amount of acid and yield of products are based on the single repeat unit of polystyrene (1 mmol). The pure benzoic acid and formanilides were obtained by flash chromatography on silica gel with hexane/ethyl acetate (4/1 to 1/1).

3.4 Isolation of the oxidized PS by-product

To an oven dried Schlenk tube, 104 mg of commercial PS (F.W. = 192,000), which contains 1 mmol of the single repeat unit, was added. Then the reaction tube was vacuumed and purged with oxygen via an oxygen balloon. Benzene (0.5 mL) and CH_3CN (0.5 mL) were injected

through a syringe. Finally, after the addition of *p*TsOH·H_2O (9.5 mg, 5 mol%), the reaction tube was allowed to stir at room temperature under 405 nm for 15 h. After the completion of the reaction, the reaction mixture was concentrated under reduced pressure to produce a yellow viscous oil, which was then washed with hot hexane (2 x 5 mL), and DCM/hexane (1/10, 5 mL). The remaining viscous product was then precipitated in water (5 mL), and finally, the oxidized PS by-product (43.6 mg) was collected after filtration and drying. The GPC characterization of the by-product in THF is shown in Figure S2. The GPC characterization of the commercial PS substrate (F.W. 192,000) was also performed as the reference (Figure S3).

**ID**	H21-6-55-2
**Calculated**	0.0000
**Recovery**	60.4986
**dn/dc**	0.1850
**Method**	20211126-0001.vcm

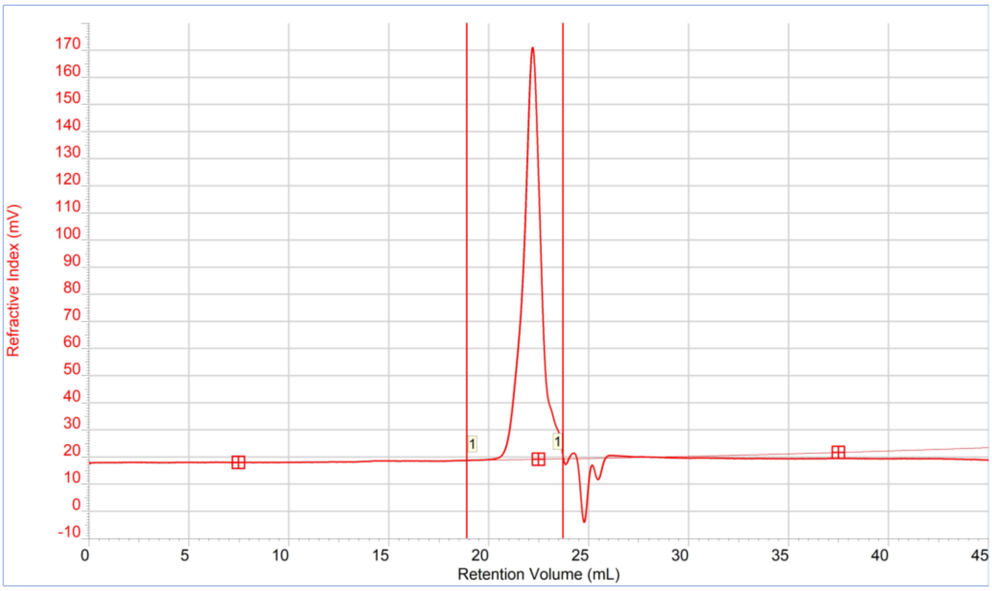

Sample	Mn	Mw	Mz	IV	Rh(w)
2021-11-26_18;48;53_H21-6-55	15,378	41,014	92,054	0.0000	0.00

Figure S2. GPC results of the oxidized PS by-product in the aerobic degradation of PS (F.W. 192,000).

ID	H21-6-55-4
Calculated	0.0000
Recovery	78.5503
dn/dc	0.1850
Method	20211126-0001.vcm

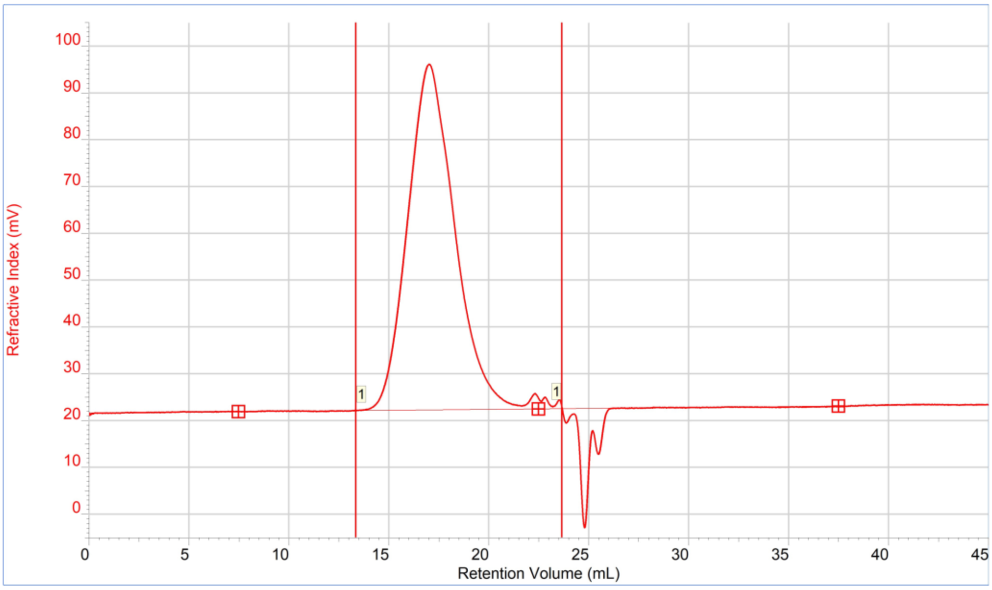

Sample	Mn	Mw	Mz	IV	Rh(w)
2021-11-26_21;52;40_H21-6-55	26,934	172,389	329,367	0.0000	0.00

Figure S3. GPS results of commercial PS (F.W. 192,000).

4. The aerobic degradation of PS in flow

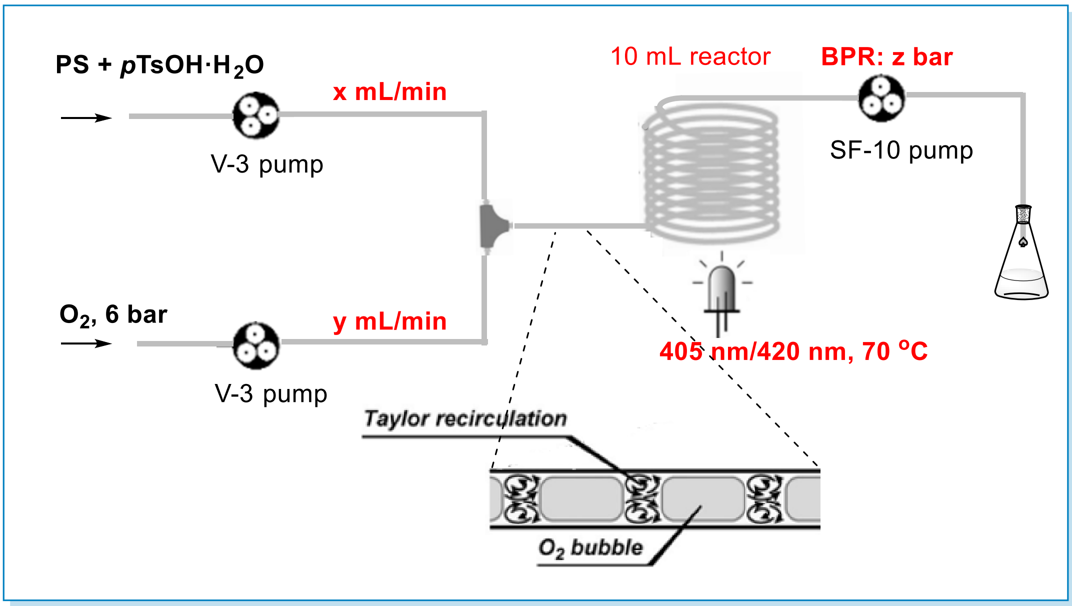

Figure S4. Schematic showing of the set up for the aerobic degradation of PS in flow.

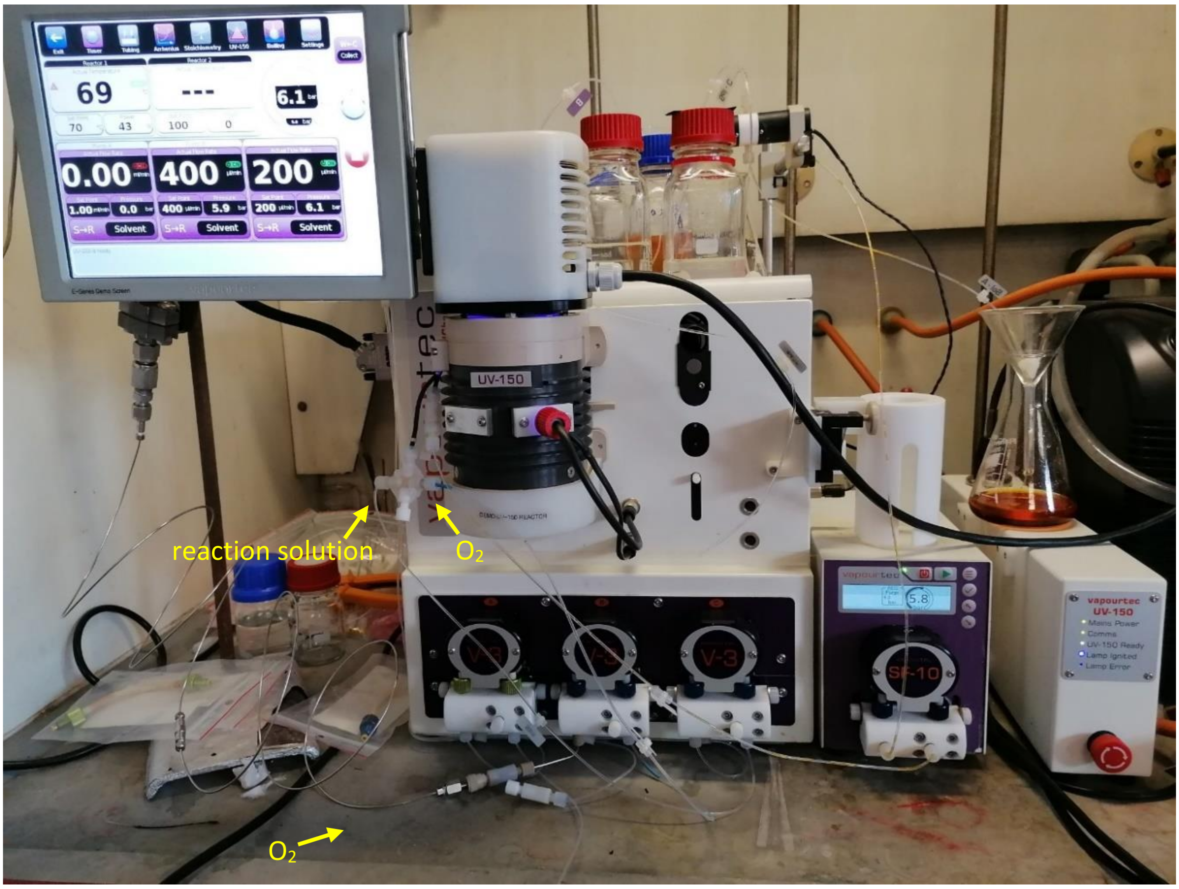

Figure S5. Set up for the aerobic degradation of PS in flow with E-Series system from Vapourtec Ltd (use of the image is with permission from Vapourtec).

Table S2. The optimization of aerobic oxidation of PS in flow

Entry	t_r (min)	BPR (bar)	T (°C)	O_2 (mL/min)	Reaction mixture (mL/min)	Light Source	Yield [%][b]	
							**1**	**2**
1	16.7	0	80	0.5	0.1	405 nm, 60 W	0	0
2	16.7	2	80	0.5	0.1	405 nm, 60 W	6	8
3	16.7	4	80	0.5	0.1	405 nm, 60 W	11	14
4	16.7	6	80	0.5	0.1	405 nm, 60 W	15	24
5	16.7	8	80	0.5	0.1	405 nm, 60 W	7	10
6	16.7	6	70	0.5	0.1	405 nm, 60 W	16	24
7	16.7	6	50	0.5	0.1	405 nm, 60 W	<1	<1
8	25	6	70	0.3	0.1	405 nm, 60 W	8	14
9	12.5	6	70	0.7	0.1	405 nm, 60 W	9	14
10	8.3	6	70	1.0	0.2	405 nm, 60 W	0	0
11[b]	2 x 16.7	6	70	0.5	0.1	405 nm, 60 W	31	48
12[c]	3 x 16.7	6	70	0.5	0.1	405 nm, 60 W	38	48
13[d]	16.7	6	70	0.5	0.1	405 nm, 60 W	<1	<1
14[e]	16.7	6	70	0.5	0.1	405 nm, 60 W	5	7
15[f]	16.7	6	70	0.5	0.1	405 nm, 60 W	6	9
16[f]	16.7	6	70	0.5	0.1	420 nm, 132 W	13	27
17[f]	16.7	6	70	0.4	0.2	420 nm, 132 W	11	18
18[e,f]	16.7	6	70	0.4	0.2	420 nm, 132 W	10	17
19[b,e,f]	2 x 16.7	6	70	0.4	0.2	420 nm, 132 W	19	25
20[c,e,f]	3 x 16.7	6	70	0.4	0.2	420 nm, 132 W	35	44
21	25	6	70	0.2	0.2	420 nm, 132 W	6	10
22	12.5	6	70	0.6	0.2	420 nm, 132 W	3	6

[a]Reaction mixture: polystyrene (1 M, 5 mmol), 10 mol% of *p*TsOH • H_2O, Benzene/CH_3CN (1/1, 5 mL). [b]The reaction was cycled in the flow reactor for two times. [c]The reaction mixture was cycled in the flow reactor for three times. [d]Polystyrene (0.5 M, 2.5 mmol). [e]Polystyrene (2 M, 10 mmol). [f]5 mol% of *p*TsOH • H_2O.

General procedure: PS (0.5 – 2 M, F.W. 192,000), and *p*TsOH•H_2O (5 or 10 mol%) were dissolved in benzene/acetonitrile (1/1, 5 mL). The reaction mixture was pumped into the flow photoreactor (10 mL) at a rate of 0.1 or 0.2 mL/min. Meanwhile, oxygen was also delivered in the system at 0.2 – 0.7 mL/min. The reaction mixture and gas were mixed via a T-piece, resulting in a Taylor recirculation[2] (Figure S4 and Figure S5). The flow degradation was carried out at 50 - 80 °C under 0 – 8 bar and 405 nm/420 nm. The results are shown in Table S2. Note: The concentration of PS, catalytic amount of acid and yield of products are based on the single repeat unit of PS.

Procedure for large-scale degradation of PS waste food box in flow: PS waste food box (18.72 g, containing 180 mmol of single repeat unit), and *p*TsOH•H_2O (5 mol%, 1.71 g) were dissolved in benzene/acetonitrile (1/1, 90 mL). The reaction mixture was pumped into the flow

photoreactor (10 mL) at a rate of 0.2 mL/min. Meanwhile, oxygen was also delivered in the system at a rate of 0.4 mL/min. The reaction mixture and gas were mixed via a T-piece, resulting in a Taylor recirculation. The flow degradation was carried out at 70 ^{o}C under 6 bar and 420 nm (132 W). The reaction mixture was cycled in the flow photoreactor three time before the analysis of products. The yield of formic acid was determined by ^{1}H NMR with 1,3,5-trimethoxybenzene as internal standard. Pure benzoic acid was isolated via following procedure: Firstly, the resulting solution after flow was alkalized to pH = 10 with aqueous solution of NaOH (0.5 M) and stirred for 10 mins. The aqueous phase was separated and collected via a separatory funnel. Meanwhile, the organic phase was further washed with H_2O (50 mL x 2), and aqueous phases were collected. Then, the combined aqueous phase was acidified to pH = 2 with 2 M HCl and stirred for 10 mins. After that, the resulting acidic solution was extracted with ethyl acetate (100 mL x 3), and the combined organic phase was dried over Na_2SO_4, and concentrated under reduced pressure to obtain the crude oily product. Finally, benzoic acid was extracted from the oily product by washing with hot hexane (50 mL x 3), and by concentrating the corresponding hexane washes. Crude benzoic acid was then recrystallized by dissolving in a minimum volume of 95°C distilled water (35 mL per 1g of crude material), filtered while hot and cooled slowly. Benzoic acid was obtained as colourless needle shape crystals. Note: The catalytic amount of acid and yield of products are based on the single repeat unit of PS.

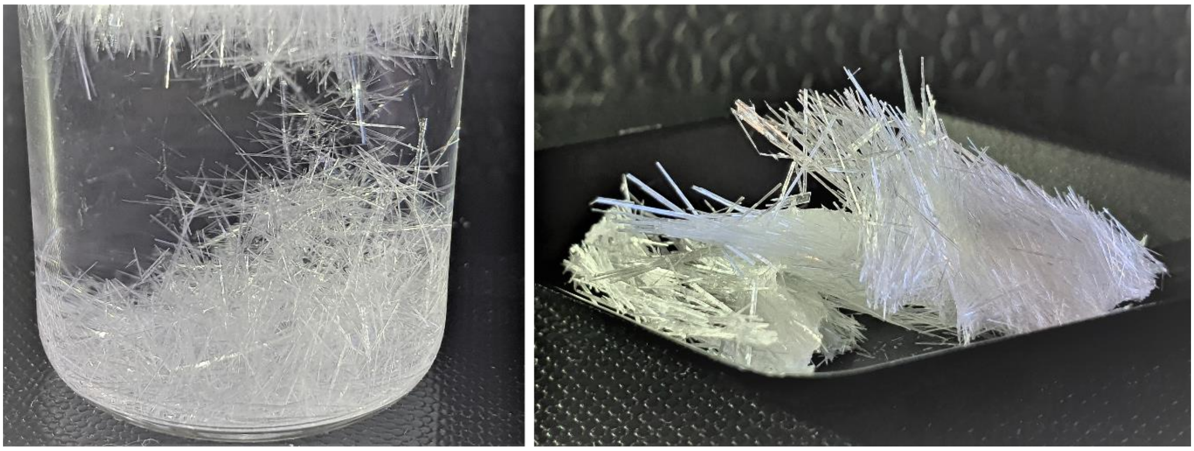

Scheme S1 Crystals of benzoic acid.

5. Mechanistic investigation

5.1 Control experiments

To an oven dried Schlenk tube, 104 mg of PS (F.W. = 192,000), which contains 1 mmol of the single repeat unit, was added. Then the reaction tube was vacuumed and purged with oxygen via an oxygen balloon. Benzene (0.5 mL) and CH_3CN (0.5 mL) were injected through a syringe. Finally, after the addition of *p*TsOH·H_2O (9.5 mg, 5 mol%) and NaN_3 or DPA or TEMPO (0.5 mmol), the reaction tube was allowed to stir at room temperature under 405 nm for 15 h. After the completion of the reaction, 1,3,5-trimethoxybenzene (50 mg) was added as internal standard, and 0.1 mL of the resulting mixture was taken out to dissolve in CD_3Cl (0.4 mL) for ^1H NMR yield analysis.

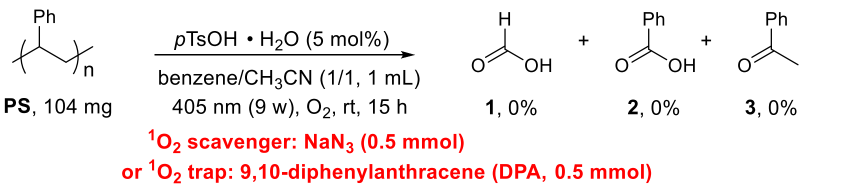

(1)

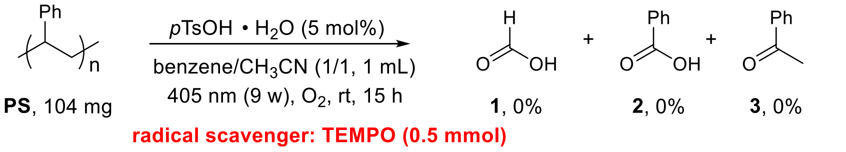

(2)

5.2 UV-Vis experiments

UV-Vis spectrum of PS: To an oven dried Schlenk tube, PS (10.4 mg, 0.1 mmol of single repeat unit, F.W. 192,000) was added. The reaction tube was vacuumed and purged with nitrogen three times. DCE (5 mL) and acetonitrile (5 mL) were injected through a syringe. Then, the mixture was stirred for 1 hour at room temperature in dark. The solution was then directly used for UV-Vis analysis.

UV-Vis spectrum of pTsOH·H₂O: To an oven dried Schlenk tube, *p*TsOH·H_2O (19.0 mg, 0.1 mmol) was added. The reaction tube was vacuumed and purged with nitrogen three times. DCE (5 mL) and acetonitrile (5 mL) were injected through a syringe. Then, the mixture was stirred for 1 hour at room temperature in dark. After that, the solution was directly used for UV-Vis analysis.

UV-Vis spectrum of the mixture of PS and pTsOH·H₂O: To an oven dried Schlenk tube, PS (10.4 mg, 0.1 mmol of single repeat unit, F.W. 192,000) and *p*TsOH·H_2O (19.0 mg, 0.1 mmol)

were added. The reaction tube was vacuumed and purged with nitrogen three times. DCE (5 mL) and acetonitrile (5 mL) were injected through a syringe. Then, the mixture was stirred for 1 hour at room temperature in dark. The solution was then directly used for UV-Vis analysis.

UV-Vis spectrum of the mixture of PS and H_2SO_4: To an oven dried Schlenk tube, PS (10.4 mg, 0.1 mmol of single repeat unit, F.W. 192,000) and H_2SO_4 (9.8 mg, 0.1 mmol) were added. The reaction tube was vacuumed and purged with nitrogen three times. DCE (5 mL) and acetonitrile (5 mL) were injected through a syringe. Then, the mixture was stirred for 1 hour at room temperature in dark. The solution was then directly used for UV-Vis analysis.

UV-Vis spectrum of the mixture of PS and HOTf: To an oven dried Schlenk tube, PS (10.4 mg, 0.1 mmol of single repeat unit, F.W. 192,000) and HOTf (15.0 mg, 0.1 mmol) were added. The reaction tube was vacuumed and purged with nitrogen three times. DCE (5 mL) and acetonitrile (5 mL) were injected through a syringe. Then, the mixture was stirred for 1 hour at room temperature in dark. The solution was then directly used for UV-Vis analysis.

UV-Vis spectrum of oxidized PS by-product: To an oven dried Schlenk tube, oxidized PS by-product (3 mg) was added. The reaction tube was vacuumed and purged with nitrogen three times. DCE (3 mL) was injected through a syringe. Then, the mixture was stirred for 1 min and directly used for UV-Vis analysis.

5.3 EPR experiments

Experimental Methods: All solvents and reagents were of analytic grade and were purchased from Sigma-Aldrich (Dorset, UK) and used without further purification. All EPR samples were prepared in 1:1 benzene:acetonitrile solvents under aerobic conditions, unless otherwise specified. The relative concentrations of PS (single repeat unit):*p*TsOH•H_2O:4-oxo-TMP:DMPO for spin trapping experiments were 10:0.5:1:4 (1 M : 50 mM : 100 mM : 400 mM) unless otherwise specified. Samples were transferred into 1.3 mm outer diameter (1 mm inner diameter) silica capillary EPR tubes (80 mm height; Wilmad LabGlass) and inserted into a 4 mm outer diameter (3 mm inner diameter) quartz tube. The position of the sample in the EPR resonator was optimised from measurements on a TEMPO standard solution. *In situ* optical irradiation at 405 nm used a Thorlabs Mounted High Power LED (M405L3)[3] with the output beam directed using an optical fibre. Optimal output (980 mW typical) was maintained by driving with a constant current of 0.7 A from a Thorlabs (LEDD1B) LED Driver.[4] All EPR samples were measured at room temperature on a Bruker EMXmicro spectrometer equipped

with a Bruker ER4112SHQ X-band resonator. Spectrometer settings were: microwave power 30 dB (0.22 mW), modulation amplitude 0.5 G, time constant 82 ms, conversion time 10 ms, sweep time 30 s, receiver gain 30 dB and an average microwave frequency of 9.86 GHz. Spectrum analysis and simulation used the EasySpin toolbox (5.2.28) for the Matlab program package.[5] The extracted spin-Hamiltonian parameters for the various DMPO-adducts are consistent with previously reported values.[6] Oxygen-centred and carbon-centred DMPO-radical adducts give quite distinct spectra due to their characteristic hyperfine coupling constants due to interaction of the unpaired electron with the ^{14}N (nuclear spin, I = 1) and β-^{1}H (sometimes γ-^{1}H) nuclei. Note some studies under oxidative conditions report decomposition of DMPO to "DMPO-X", which has a characteristic ^{14}N coupling of 0.72 mT: we did not observe this in any of our experiments.

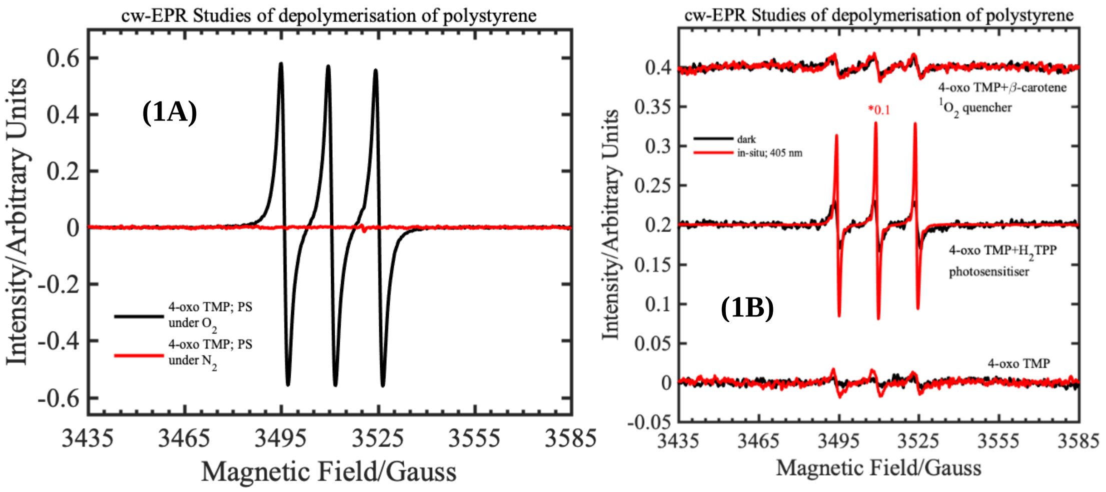

Figure S6. A: cw-EPR spectra of PS + pTsOH•H_2O + 4-oxo-TMP under air (black) and N_2 (red) atmospheres after irradiation with 405 nm LED. **B**: cw-EPR spectra of 4-oxo-TMP before (black) and after (red) irradiation with 405 nm LED (bottom), in the presence of the photosensitiser H_2TPP (middle) and the $^{1}O_2$ quencher β-carotene (top).

Because of the short-lived nature of the radical species, the irradiation experiments were performed *in situ* in the EPR resonator. There are some differences from the actual depolymerisation reactions performed as described above: a 980 mW output power LED was used in comparison to the 9 W LED used for the catalysis studies, and samples were under air rather than O_2 atmosphere. Care needs taken in interpreting the EPR spin trapping data because DMPO can itself trap $^{1}O_2$ (producing radical species),[7] and (as noted in the main text) TEMPO and related nitroxides can act as radical scavengers (hence reaction inhibitors) including under acidic conditions.[8] Moreover, nitroxyls can be unstable under acidic conditions.[9] Hence, the

spin traps are not necessarily innocent in the chemistry being studied. Consequently, we have performed control experiments in the absence of the PS substrate and/or the *p*TsOH·H_2O acid catalyst against which to compare the results. We also performed additional experiments using standard $^1O_2$ photosensitisers.

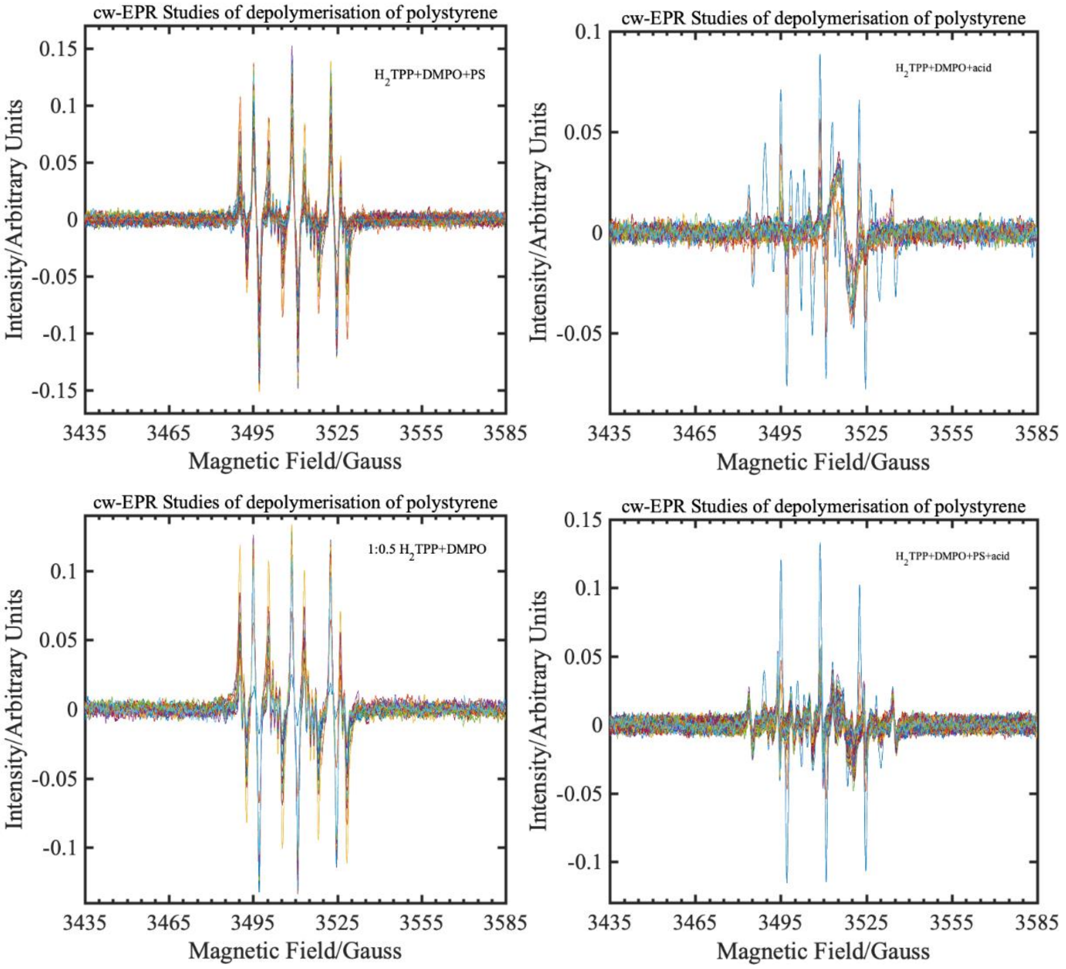

Figure S7. Control cw-EPR *in situ* irradiation (405 nm LED) spin trapping (DMPO) experiments with established H_2TPP photosensitiser, monitored as a function of time for every 8 minutes for a minimum of 3 hours/maximum of 24 hrs. Sensitiser and DMPO (top left); with PS substrate (top right); with *p*- *p*TsOH·H_2O acid catalyst (bottom left); with substrate and acid (bottom right). The addition of the acid catalyst triggers more rapid growth then decay of DMPO-adducts, including C-centred DMPO radical adducts. The addition of PS into the reaction mixture (H_2TPP + DMPO + acid) enhances the growth and persistence of the signal from C-centred DMPO radical adducts.

Figure S6 illustrates generation of 4-oxo-TEMPO under 405 nm irradiation of the reaction system containing 4-oxo-TMP, a standard $^1O_2$ spin trap. The signal is absent when this

experiment is performed under an N_2 atmosphere. Figure S7 shows results in the presence of an established $^1O_2$ photosensitiser (H_2TPP), in the absence and presence of substrate and acid catalyst. The experiments confirm that $^1O_2$ can generate free radicals in the presence of the spin trap DMPO alone, hence care must be taken in the interpretation of the experiments with the PS substrate. In the absence of acid we observe O-centred DMPO radical adducts and an unidentified nitroxyl (three-line spectrum). In the presence of an acid, we observe more rapid generation of radicals and the generation of C-centred DMPO radical adducts. In the presence of PS and acid, we see more C-centred radicals over a longer timescale: this is illustrated in Figure S8, which shows the spectra after 3 hours of irradiation with and without PS substrate. Spectra measured without the efficient H_2TPP photosensitiser are weaker (Figure S9). Figure S10 shows the time evolution of the three spectral components detected on irradiation in the presence of both 4-oxo-TMP and DMPO (1:4) spin traps (see Figure 1, main text). We do not observe the DMPO-adduct spectra at lower concentrations of DMPO.

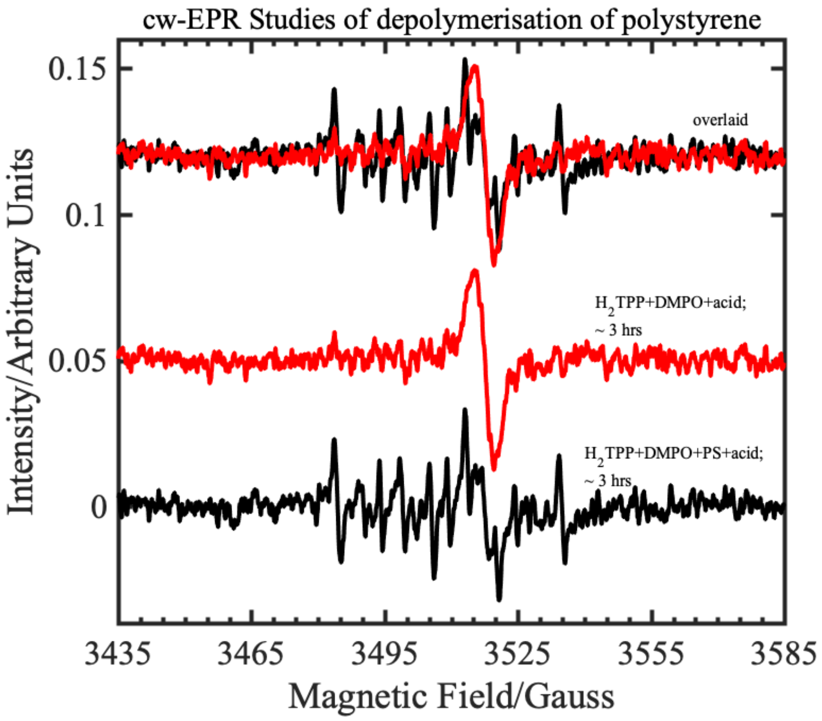

Figure S8. Control cw-EPR *in situ* irradiation (405 nm LED) spin trapping (DMPO) experiments with established H_2TPP photosensitiser. Spectra of H_2TPP + DMPO + *p*TsOH•H_2O after 3 hours of *in situ* irradiation in the presence (black) and absence (red) of PS. This comparison shows that the presence of PS leads to increased trapping of C-centred DMPO-adducts.

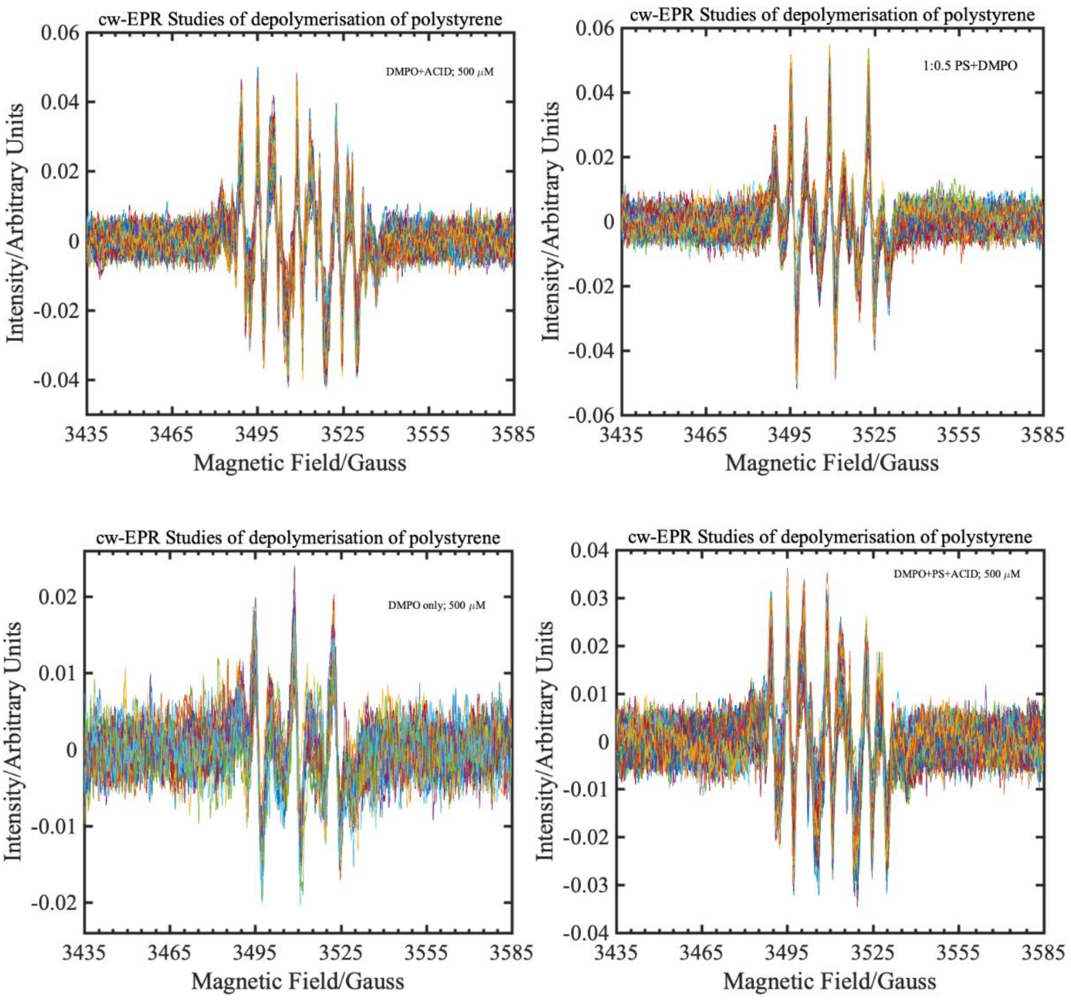

Figure S9. cw-EPR spectra of DMPO solutions monitored as a function of time for every 8 minutes for a minimum of 3 hours/maximum of 13 hrs of *in situ* 405 nm LED irradiation (top left), with PS substrate (top right), with *p*TsOH·H_2O acid catalyst (bottom left) and substrate and acid (bottom right). The addition of acid catalyst and PS increases the signal from the DMPO-adducts by a factor of *ca*. two. In reactions with acid, there is an increase in the signals due to O-centred DMPO-radical adducts, and trace amounts of C-centred DMPO-radical adducts can be observed in the wings of the spectrum.

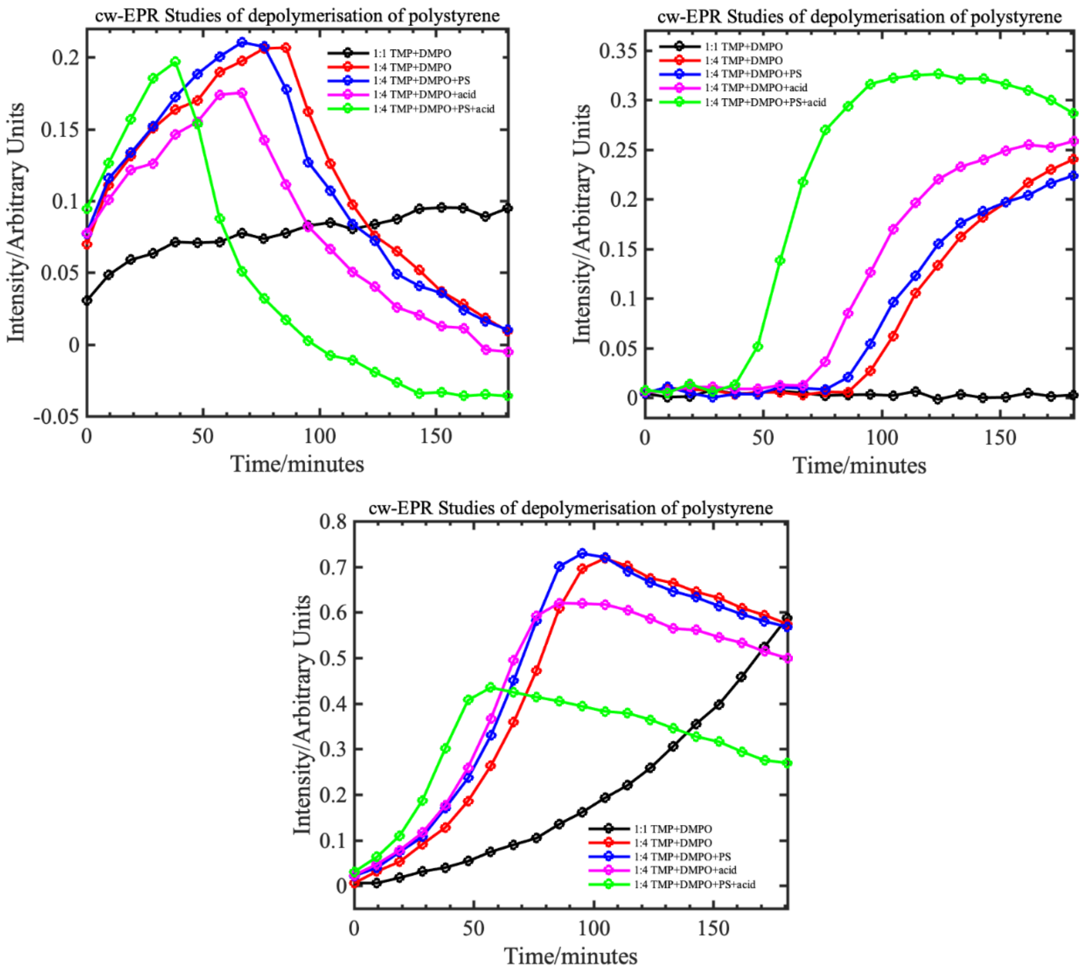

Figure S10. Time evolution of various DMPO-adducts (monitored by EPR) formed in the presence of the $^1O_2$ molecular probe 4-oxo-TMP on *in situ* irradiation at 405 nm. Top (left): O-centred DMPO-radical adduct. Top (right): C-centred DMPO-radical adduct. Bottom: 4-oxo-TEMPO and an unknown DMPO-adduct. The intensities were monitored by EPR at the magnetic fields in the main Figure **1**. The effects of PS substrate (blue), *p*-TsOH**.**H_2O acid catalyst (magenta), and both together (green) on the time-evolution are shown. At lower concentrations of DMPO (black) we do not observe the C-centred DMPO-adduct clearly.

5.4 DFT calculations

5.4.1 Computational details

All density functional theory (DFT) calculations were carried out using the Gaussian 16 software package.[10] All geometries were optimized using the B3LYP hybrid functional[11-12] and Grimme's D3(BJ) dispersion correction (GD3BJ)[13-14] with a basis set of 6-31G(d) for all atoms. Vibrational frequency calculations were performed for all the stationary points to confirm if each optimized structure is a local minimum on the respective potential energy surface or a

transition state structure with only one imaginary frequency. Solvation energy corrections were calculated in acetonitrile solvent with the SMD continuum solvation model[15] based on the gas phase optimized geometries. Additional calculations were performed with 1,2-dichloroethane as solvent. The same functional with a basis set of 6-311+G(d,p) for all atoms was used for single-point energy calculations.

5.4.2 The radical initiation step with hydroperoxyl radicals

Considering that the generated hydroperoxyl radical may escape from the solvent cage after the HAT transition state **^{1}TS-1**, we also investigated the radical initiation process using the hydroperoxyl radical. Computational results show that the HAT with hydroperoxyl radical has a moderate energy barrier of 21.6 kcal/mol (Figure S11), which is higher than that with singlet oxygen and the hydroxyl radical while lower than that with triplet oxygen and the superoxide radical. Furthermore, this reaction is exergonic by 2.9 kcal/mol with respect to **7**, indicating the thermodynamic feasibility of the pathway.

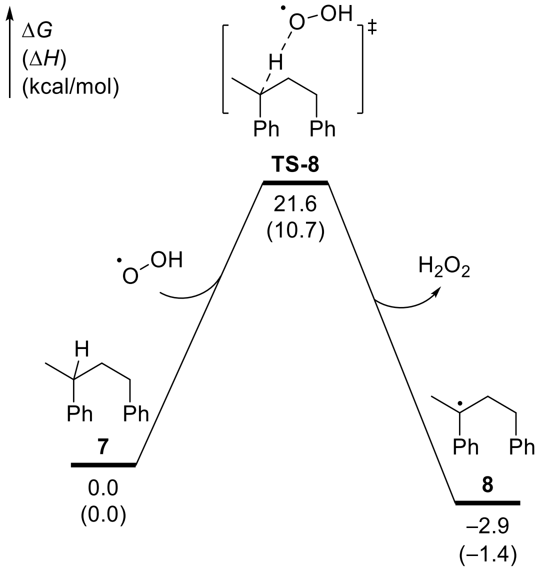

Figure S11. Computational results of the radical initiation with the hydroperoxyl radical.

5.4.3 A competitive C-C bond cleavage pathway from the oxygen-centered radical 11

Computational results of the competitive C–C bond cleavage pathway leading to ketone **13** and methyl radical **14** are shown in the Figure S12. This C–C bond cleavage pathway through **TS-9** has an energy barrier of 7.3 kcal/mol, which is 3.3 kcal/mol higher than that of **TS-6**. The higher energy barrier is attributed to the lower stability of methyl radical. Therefore, this possibility is demonstrated to be disfavored compared with the main pathway shown in Scheme 8 (manuscript).

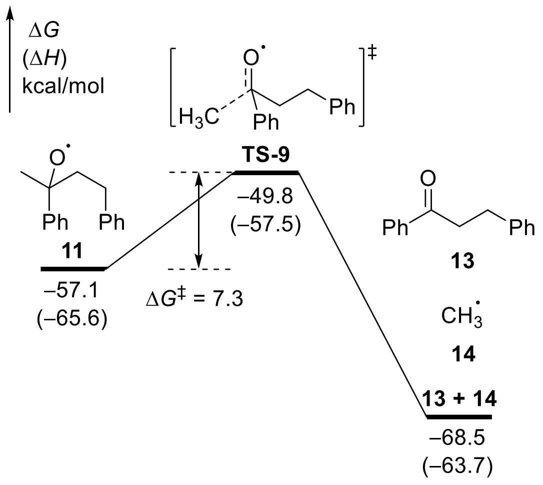

Figure S12. Free energy profile of the competitive C–C bond cleavage pathway**.** All energies are with respect to **7.**

5.4.4 Computational studies of the reaction between alkyl radical 8 and the triplet oxygen molecule

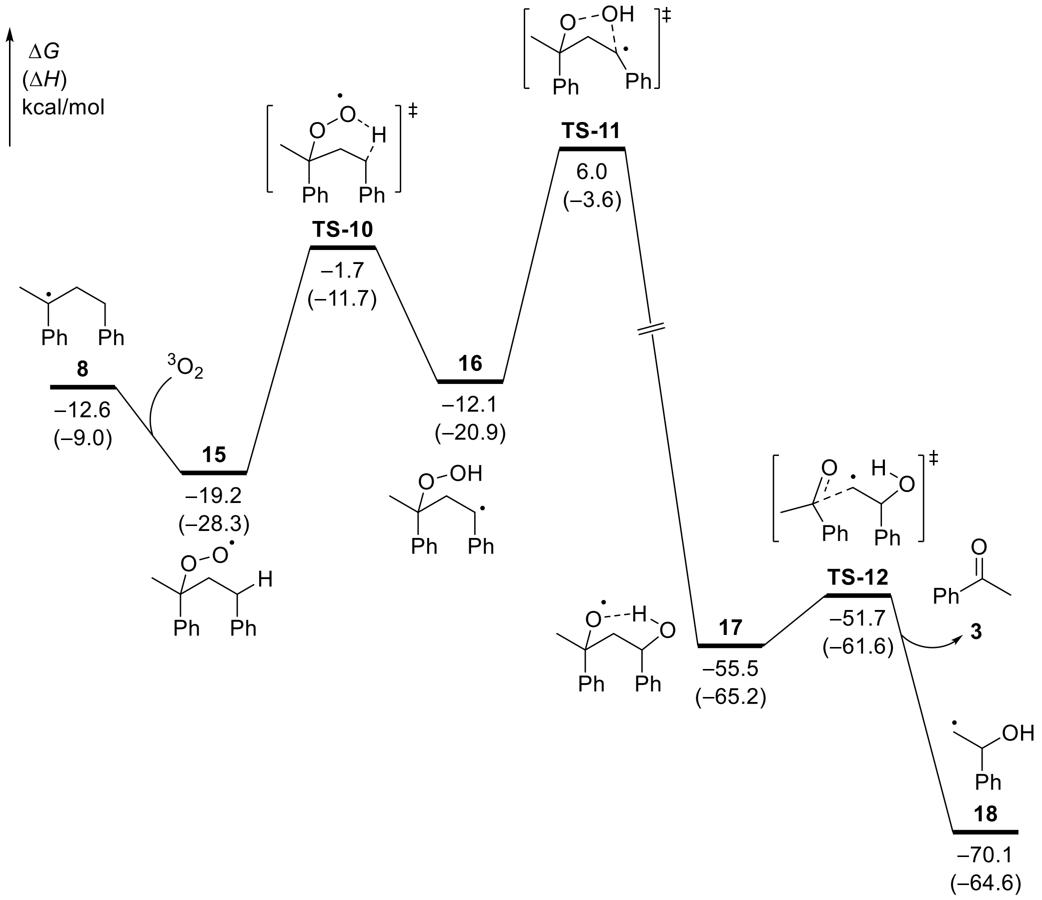

Figure S13. Free energy profile of the reaction between alkyl radical **8** and the triplet oxygen molecule. All energies are with respect to **7**.

Besides the radical rebound between alkyl radical **8** and hydroperoxyl radical shown in Scheme 8, the reaction of alkyl radical **8** with the triplet oxygen molecule is also studied (Figure S13). The radical capture is shown to be exergonic by 6.6 kcal/mol of free energy. From the oxygen-centered radical **15**, intramolecular HAT can occur through **TS-10** and generate the alkyl radical **16**. The energy barrier is 17.5 kcal/mol. Subsequently, the intramolecular hydroxyl transfer may occur through **TS-11** to generate the oxygen-centered radical **17**. The following *β*-scission via **TS-12** can realize the C–C bond cleavage easily. Although the formation of radical **17** is highly exergonic, the overall activation free energy is demonstrated to be 25.2 kcal/mol. The higher energy barrier compared with that shown in Scheme 8 renders this pathway kinetically disfavored.

5.4.5 Computational results calculated in 1,2-dichloroethane solvent

We calculated the reactions of the oxidation of 1,3-diphenylbutane **7** with $^1O_2$ to generate **9** and the radical substitution of **9** with •OH in the 1,2-dichloroethane solvent. The computational results suggest that the activation free energies of **^{1}TS-1** and **TS-5** are close to those calculated in acetonitrile.

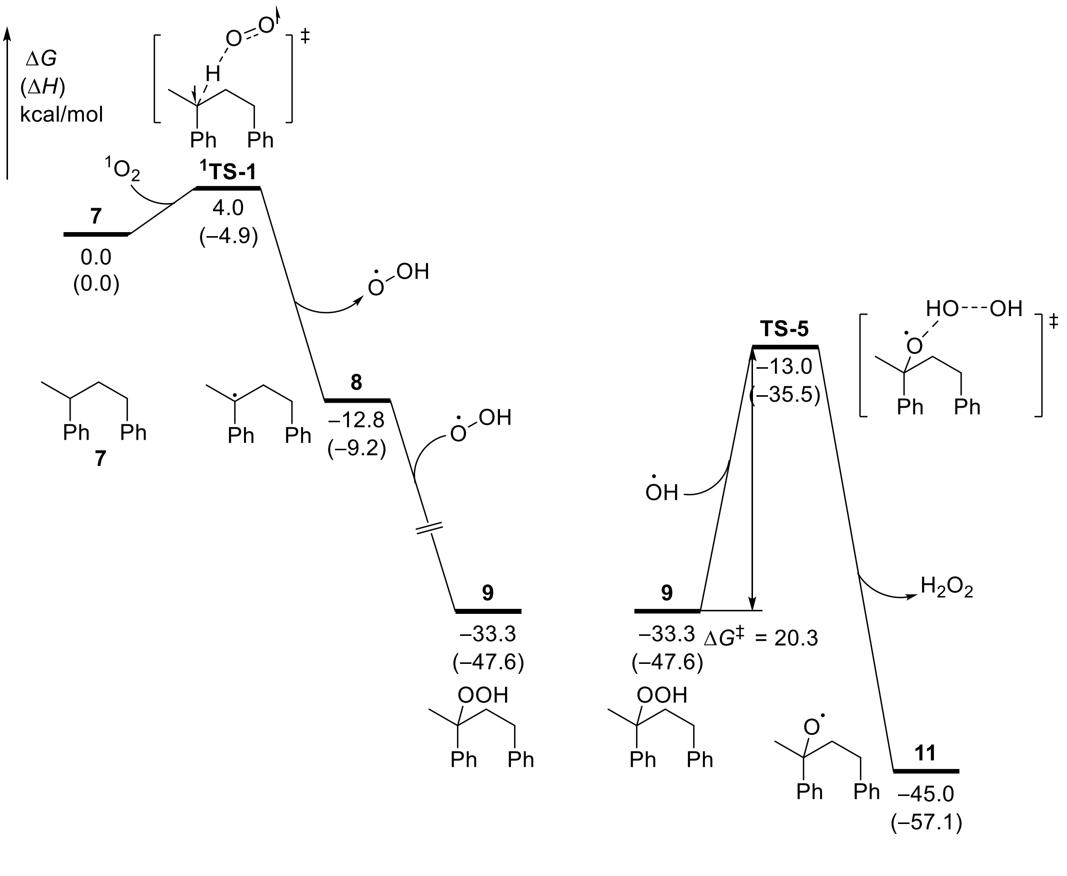

Figure S14. Computational results calculated in 1,2-dichloroethane solvent.

5.4.6 Cartesian Coordinates (Å) and Energies of Optimized Structures

3

B3LYP SCF energy: -384.91824647 a.u.

B3LYP enthalpy: -384.770871 a.u.

B3LYP free energy: -384.812233 a.u.

B3LYP SCF energy in acetonitrile solution: -385.04594426 a.u.

B3LYP enthalpy in acetonitrile solution: -384.898569 a.u.

B3LYP free energy in acetonitrile solution: -384.939931 a.u.

Cartesian coordinates

ATOM	X	Y	Z
C	1.695666	-0.204304	0.000045
C	2.552677	1.049985	-0.000094
H	2.346518	1.666003	-0.883361
H	2.346564	1.666165	0.883080
H	3.603445	0.756411	-0.000102
C	0.205852	-0.055490	-0.000020
C	-0.575617	-1.220916	-0.000053
C	-0.429133	1.194661	0.000038
C	-1.963961	-1.138454	-0.000063
H	-0.065297	-2.178099	-0.000081
C	-1.821120	1.277463	0.000049
H	0.158285	2.107057	-0.000019
C	-2.589546	0.112113	0.000015
H	-2.561831	-2.045373	-0.000165

H	-2.305436	2.249674	0.000135
H	-3.674028	0.177443	0.000068
O	2.212859	-1.311202	0.000117

7

B3LYP SCF energy: -620.61347738 a.u.

B3LYP enthalpy: -620.301891 a.u.

B3LYP free energy: -620.358308 a.u.

B3LYP SCF energy in acetonitrile solution: -620.80246538 a.u.

B3LYP enthalpy in acetonitrile solution: -620.490879 a.u.

B3LYP free energy in acetonitrile solution: -620.547296 a.u.

B3LYP SCF energy in 1, 2-dichloroethane solution: -620.80286175 a.u.

B3LYP enthalpy in 1, 2-dichloroethane solution: -620.491276 a.u.

B3LYP free energy in 1, 2-dichloroethane solution: -620.547692 a.u.

Cartesian coordinates

ATOM	X	Y	Z
C	0.186259	2.178038	-0.968324
H	0.492597	1.887017	-1.981012
H	-0.097047	3.237452	-1.024426
C	1.396936	2.059735	-0.016858
H	2.062351	2.915842	-0.188999

H	1.051049	2.149066	1.021228
C	2.243302	0.780161	-0.175464
H	2.632237	0.779896	-1.203597
C	3.449403	0.817058	0.779999
H	3.117810	0.819541	1.824943
H	4.088612	-0.060279	0.636151
H	4.054370	1.716717	0.615488
C	-1.023838	1.370659	-0.548654
C	-1.462495	0.270148	-1.290634
C	-1.718958	1.703961	0.621336
C	-2.544364	-0.498297	-0.862376
H	-0.928828	-0.007891	-2.194632
C	-2.804248	0.943034	1.052700
H	-1.399847	2.565058	1.204888
C	-3.217776	-0.167035	0.313552
H	-2.853549	-1.362536	-1.443425
H	-3.327862	1.216324	1.965094
H	-4.058754	-0.766616	0.650740
C	1.455530	-0.505740	0.004460
C	1.459573	-1.482966	-0.995443
C	0.714914	-0.749433	1.168636
C	0.729008	-2.664202	-0.852233
H	2.033285	-1.310139	-1.903399
C	-0.022968	-1.920921	1.314326

H	0.687566	-0.001770	1.955846
C	-0.020780	-2.883718	0.302128
H	0.741433	-3.407095	-1.645420
H	-0.611420	-2.078074	2.213737
H	-0.600998	-3.795403	0.413880

8

B3LYP SCF energy: -619.96890717 a.u.

B3LYP enthalpy: -619.670697 a.u.

B3LYP free energy: -619.728971 a.u.

B3LYP SCF energy in acetonitrile solution: -620.15845984 a.u.

B3LYP enthalpy in acetonitrile solution: -619.860250 a.u.

B3LYP free energy in acetonitrile solution: -619.918524 a.u.

B3LYP SCF energy in 1, 2-dichloroethane solution: -620.15893034 a.u.

B3LYP enthalpy in 1, 2-dichloroethane solution: -619.860720 a.u.

B3LYP free energy in 1, 2-dichloroethane solution: -619.918994 a.u.

Cartesian coordinates

ATOM	X	Y	Z
C	1.470499	2.146954	-0.212017
H	1.348248	2.739309	0.702887
H	2.235409	2.647132	-0.818708

C	0.128114	2.163496	-1.004971
H	-0.058938	3.199660	-1.318991
H	0.259515	1.579651	-1.921264
C	-1.044680	1.679508	-0.201063
C	-1.662620	2.672418	0.746270
H	-2.700014	2.916002	0.471152
H	-1.694775	2.299530	1.779337
H	-1.100702	3.611651	0.752047
C	1.909811	0.745079	0.126691
C	1.471199	0.118321	1.298883
C	2.685982	0.006747	-0.775435
C	1.781545	-1.216506	1.555162
H	0.863116	0.677225	2.005136
C	3.001653	-1.328453	-0.523198
H	3.037385	0.482673	-1.688579
C	2.544314	-1.946117	0.642232
H	1.421371	-1.689188	2.464599
H	3.603920	-1.885909	-1.235569
H	2.784029	-2.987229	0.839095
C	-1.530959	0.339927	-0.262854
C	-2.674159	-0.059768	0.489240
C	-0.902173	-0.669325	-1.048153
C	-3.144236	-1.364806	0.458431
H	-3.194201	0.671507	1.098354

C -1.375195 -1.971826 -1.066136

H -0.010087 -0.432360 -1.613706

C -2.500769 -2.336197 -0.316758

H -4.020045 -1.631282 1.044539

H -0.856935 -2.716717 -1.664005

H -2.867247 -3.358379 -0.334260

9

B3LYP SCF energy: -770.95623428 a.u.

B3LYP enthalpy: -770.635106 a.u.

B3LYP free energy: -770.696658 a.u.

B3LYP SCF energy in acetonitrile solution: -771.20725508 a.u.

B3LYP enthalpy in acetonitrile solution: -770.886127 a.u.

B3LYP free energy in acetonitrile solution: -770.947679 a.u.

B3LYP SCF energy in 1, 2-dichloroethane solution: -771.20635087 a.u.

B3LYP enthalpy in 1, 2-dichloroethane solution: -770.885223 a.u.

B3LYP free energy in 1, 2-dichloroethane solution: -770.946775 a.u.

Cartesian coordinates

ATOM X Y Z

C -0.420690 -2.042303 -0.531208

H -0.836300 -1.755912 -1.502271

H	-0.423634	-3.139470	-0.500011
C	-1.371458	-1.542839	0.576108
H	-2.241799	-2.204276	0.620695
H	-0.878013	-1.605689	1.552374
C	-1.915962	-0.112861	0.380164
C	-2.881508	0.261684	1.513668
H	-2.352429	0.292199	2.470445
H	-3.305492	1.254506	1.330052
H	-3.690245	-0.470880	1.591115
C	1.005526	-1.550923	-0.408434
C	1.551487	-0.660863	-1.338275
C	1.804834	-1.963723	0.665829
C	2.847740	-0.169993	-1.185345
H	0.940927	-0.324078	-2.170670
C	3.102827	-1.480106	0.822630
H	1.401269	-2.666787	1.391825
C	3.627740	-0.574604	-0.101682
H	3.243910	0.537135	-1.908748
H	3.705461	-1.810505	1.664728
H	4.636770	-0.190689	0.020844
C	-0.815345	0.941214	0.272371
C	-0.748680	1.835463	-0.800751
C	0.150693	1.030456	1.283068
C	0.277688	2.780006	-0.872796

H -1.489548 1.783866 -1.588773

C 1.175519 1.968715 1.209514

H 0.123893 0.341595 2.121148

C 1.245794 2.847367 0.126650

H 0.318952 3.460779 -1.718849

H 1.928155 2.003095 1.991580

H 2.049722 3.575342 0.064387

O -2.603972 -0.036248 -0.893799

O -3.707691 -0.978553 -0.893822

H -4.455535 -0.361965 -0.797964

10

B3LYP SCF energy: -310.22956515 a.u.

B3LYP enthalpy: -310.078793 a.u.

B3LYP free energy: -310.120395 a.u.

B3LYP SCF energy in acetonitrile solution: -310.32841015 a.u.

B3LYP enthalpy in acetonitrile solution: -310.177638 a.u.

B3LYP free energy in acetonitrile solution: -310.219240 a.u.

Cartesian coordinates

ATOM X Y Z

C -1.964702 -0.443881 0.392331

H	-2.170259	-1.522922	0.270331
H	-2.242981	-0.221541	1.434260
C	-2.824544	0.357391	-0.529818
H	-3.861246	0.561734	-0.282380
H	-2.478729	0.598573	-1.529586
C	-0.478339	-0.210299	0.200738
C	0.402274	-1.278320	0.001392
C	0.042291	1.090805	0.224797
C	1.770846	-1.057359	-0.165792
H	0.011774	-2.293016	-0.024712
C	1.407679	1.316677	0.058547
H	-0.634469	1.928750	0.371872
C	2.278041	0.241645	-0.137426
H	2.438627	-1.900411	-0.321269
H	1.793757	2.332260	0.081307
H	3.342235	0.416625	-0.268430

11

B3LYP SCF energy: -695.16161206 a.u.

B3LYP enthalpy: -694.858486 a.u.

B3LYP free energy: -694.917395 a.u.

B3LYP SCF energy in acetonitrile solution: -695.37783887 a.u.

B3LYP enthalpy in acetonitrile solution: -695.074713 a.u.

B3LYP free energy in acetonitrile solution: -695.133622 a.u.

B3LYP SCF energy in 1, 2-dichloroethane solution: -695.37762332 a.u.

B3LYP enthalpy in 1, 2-dichloroethane solution: -695.074497 a.u.

B3LYP free energy in 1, 2-dichloroethane solution: -695.133405 a.u.

Cartesian coordinates

ATOM	X	Y	Z
C	-0.274157	-2.270594	-0.632566
H	-0.647279	-2.131467	-1.653656
H	-0.081917	-3.344558	-0.512960
C	-1.394000	-1.886748	0.358320
H	-2.171807	-2.657429	0.325649
H	-0.997893	-1.867794	1.379826
C	-2.099503	-0.541442	0.037258
C	-3.303441	-0.361652	1.031300
H	-2.897560	-0.296956	2.044823
H	-3.838406	0.561941	0.800781
H	-3.984954	-1.212823	0.958635
C	1.019509	-1.511822	-0.437540
C	1.418202	-0.509324	-1.326895
C	1.826580	-1.775279	0.676655
C	2.574559	0.234320	-1.094990
H	0.798619	-0.288554	-2.191150

C	2.986845	-1.038952	0.911467
H	1.536641	-2.561029	1.371520
C	3.361591	-0.025421	0.027431
H	2.854655	1.023255	-1.787079
H	3.598886	-1.256416	1.782856
H	4.261530	0.554940	0.210820
C	-1.194758	0.692118	0.146884
C	-1.202015	1.650419	-0.869451
C	-0.358986	0.884113	1.252716
C	-0.369768	2.768004	-0.795831
H	-1.853759	1.500664	-1.722812
C	0.478494	1.994967	1.324429
H	-0.333335	0.150661	2.052578
C	0.478514	2.940798	0.297639
H	-0.379567	3.500012	-1.599078
H	1.139057	2.115472	2.178114
H	1.136003	3.804022	0.350621
O	-2.728114	-0.631873	-1.178806

12

B3LYP SCF energy: -386.10766395 a.u.

B3LYP enthalpy: -385.935971 a.u.

B3LYP free energy: -385.979132 a.u.

B3LYP SCF energy in acetonitrile solution: -386.24615036 a.u.

B3LYP enthalpy in acetonitrile solution: -386.074457 a.u.

B3LYP free energy in acetonitrile solution: -386.117618 a.u.

Cartesian coordinates

ATOM	X	Y	Z
C	1.423058	-0.812969	-0.678651
H	1.764440	-0.377783	-1.626317
C	2.433906	-0.419727	0.400507
H	3.389422	-0.934961	0.210049
H	2.063537	-0.747669	1.383871
C	0.010275	-0.373309	-0.368805
C	-0.950011	-1.304405	0.044001
C	-0.360928	0.975744	-0.459642
C	-2.250002	-0.905003	0.359085
H	-0.676960	-2.355031	0.115876
C	-1.658312	1.378233	-0.145074
H	0.379681	1.706879	-0.765523
C	-2.608225	0.439815	0.264982
H	-2.981485	-1.644461	0.673999
H	-1.929719	2.427832	-0.221849
H	-3.619763	0.754664	0.506343
O	2.605246	0.992207	0.357629

H	3.074503	1.265538	1.159235
H	1.455824	-1.902935	-0.795131

13

B3LYP SCF energy: -655.30870676 a.u.

B3LYP enthalpy: -655.044678 a.u.

B3LYP free energy: -655.100910 a.u.

B3LYP SCF energy in acetonitrile solution: -655.51467989 a.u.

B3LYP enthalpy in acetonitrile solution: -655.250651 a.u.

B3LYP free energy in acetonitrile solution: -655.306883 a.u.

Cartesian coordinates

ATOM	X	Y	Z
C	1.651392	2.055256	-0.197573
H	1.598586	2.644091	0.724450
H	2.448451	2.483806	-0.815848
C	0.305518	2.224805	-0.949590
H	0.166700	3.284006	-1.188374
H	0.328247	1.663734	-1.887800
C	-0.866257	1.823793	-0.059983
C	1.952730	0.608672	0.114078
C	1.508422	0.026749	1.307466

C	2.597934	-0.202639	-0.827541
C	1.688016	-1.335308	1.547067
H	1.000050	0.644464	2.043207
C	2.780415	-1.565694	-0.592105
H	2.953819	0.238693	-1.756222
C	2.320394	-2.137094	0.595809
H	1.328926	-1.771174	2.475072
H	3.283128	-2.180357	-1.333949
H	2.458713	-3.198574	0.780641
C	-1.516069	0.486019	-0.209000
C	-2.662859	0.230267	0.560097
C	-1.011052	-0.525729	-1.038366
C	-3.295738	-1.005914	0.495683
H	-3.032519	1.020938	1.204049
C	-1.637577	-1.769573	-1.091736
H	-0.108093	-0.366030	-1.614508
C	-2.781970	-2.010588	-0.330427
H	-4.185354	-1.192455	1.090587
H	-1.227951	-2.551316	-1.724606
H	-3.271463	-2.979540	-0.376823
O	-1.242629	2.605198	0.804605

14

B3LYP SCF energy: -39.83890415 a.u.

B3LYP enthalpy: -39.805019 a.u.

B3LYP free energy: -39.828896 a.u.

B3LYP SCF energy in acetonitrile solution: -39.85489774 a.u.

B3LYP enthalpy in acetonitrile solution: -39.821013 a.u.

B3LYP free energy in acetonitrile solution: -39.844890 a.u.

Cartesian coordinates

ATOM	X	Y	Z
C	0.000000	0.000039	0.000021
H	0.931792	0.551611	-0.000042
H	-0.943756	0.530890	-0.000042
H	0.011966	-1.082736	-0.000042

15

B3LYP SCF energy: -770.32535854 a.u.

B3LYP enthalpy: -770.016393 a.u.

B3LYP free energy: -770.077806 a.u.

B3LYP SCF energy in acetonitrile solution: -770.56746728 a.u.

B3LYP enthalpy in acetonitrile solution: -770.258502 a.u.

B3LYP free energy in acetonitrile solution: -770.319915 a.u.

Cartesian coordinates

ATOM	X	Y	Z
C	-0.419883	-2.038995	-0.582588
H	-0.820486	-1.742494	-1.557427
H	-0.410674	-3.136400	-0.573838
C	-1.394084	-1.581592	0.523640
H	-2.267600	-2.240761	0.528515
H	-0.917404	-1.678146	1.505030
C	-1.937408	-0.149287	0.388732
C	-2.923927	0.182379	1.510263
H	-2.407173	0.165976	2.474117
H	-3.340825	1.182705	1.361529
H	-3.736660	-0.548168	1.526859
C	0.998246	-1.532772	-0.428233
C	1.551431	-0.628900	-1.339950
C	1.781555	-1.949450	0.656123
C	2.840228	-0.127930	-1.159302
H	0.953666	-0.290017	-2.180855
C	3.071816	-1.455203	0.840551
H	1.372960	-2.664509	1.367410
C	3.604272	-0.535892	-0.065649
H	3.242888	0.589018	-1.869173
H	3.662669	-1.788632	1.689608

H	4.607384	-0.144269	0.078328
C	-0.861687	0.919515	0.273773
C	-0.813081	1.819780	-0.795181
C	0.106406	1.015835	1.282058
C	0.199568	2.779412	-0.864265
H	-1.555803	1.766223	-1.581285
C	1.117582	1.968613	1.209981
H	0.091529	0.321451	2.116023
C	1.170369	2.854362	0.131879
H	0.226921	3.466440	-1.705560
H	1.872619	2.009380	1.989211
H	1.962977	3.594587	0.070990
O	-2.689778	-0.081204	-0.899022
O	-3.695899	-0.931499	-0.942287

16

B3LYP SCF energy: -770.30342739 a.u.

B3LYP enthalpy: -769.996088 a.u.

B3LYP free energy: -770.058095 a.u.

B3LYP SCF energy in acetonitrile solution: -770.55399939 a.u.

B3LYP enthalpy in acetonitrile solution: -770.246660 a.u.

B3LYP free energy in acetonitrile solution: -770.308667 a.u.

Cartesian coordinates

ATOM	X	Y	Z
C	-1.207301	-1.684372	-0.561862
H	-0.559058	-1.797672	1.362573
H	-1.624394	-2.668088	-0.348718
C	0.160184	-1.664974	-1.177961
H	0.329279	-2.650510	-1.624464
H	0.189676	-0.940838	-1.999702
C	1.402557	-1.389198	-0.249366
C	2.637194	-2.066340	-0.871069
H	2.799356	-1.689681	-1.885139
H	3.524885	-1.849048	-0.271808
H	2.491292	-3.150416	-0.914557
C	-2.065870	-0.575200	-0.348571
C	-1.676734	0.770771	-0.596345
C	-3.382551	-0.794724	0.150669
C	-2.553257	1.818975	-0.358580
H	-0.677633	0.981493	-0.955860
C	-4.249801	0.260127	0.383298
H	-3.702720	-1.815264	0.346958
C	-3.843197	1.577638	0.130773
H	-2.228517	2.837970	-0.550139
H	-5.248601	0.063377	0.762977

H	-4.522468	2.404415	0.315772
C	1.710523	0.093490	-0.050846
C	1.991800	0.624212	1.212933
C	1.821685	0.936851	-1.166440
C	2.332535	1.969839	1.357538
H	1.929449	-0.012738	2.084142
C	2.164037	2.281046	-1.023288
H	1.645588	0.546502	-2.165244
C	2.413347	2.806639	0.244829
H	2.534572	2.363792	2.349797
H	2.238811	2.913534	-1.903592
H	2.675247	3.854335	0.362406
O	1.276733	-2.113006	0.984236
O	0.257558	-1.534474	1.838304

17

B3LYP SCF energy: -770.37522855 a.u.

B3LYP enthalpy: -770.066773 a.u.

B3LYP free energy: -770.127326 a.u.

B3LYP SCF energy in acetonitrile solution: -770.62575465 a.u.

B3LYP enthalpy in acetonitrile solution: -770.317299 a.u.

B3LYP free energy in acetonitrile solution: -770.377852 a.u.

Cartesian coordinates

ATOM	X	Y	Z
C	0.541817	-2.148291	0.169125
H	0.460978	-3.191031	-0.182817
C	1.519315	-1.453430	-0.806441
H	2.393495	-2.101671	-0.940195
H	1.044051	-1.339732	-1.784668
C	2.051374	-0.091400	-0.283538
C	3.178125	0.397003	-1.265770
H	2.728027	0.567508	-2.247397
H	3.598808	1.334144	-0.895984
H	3.965736	-0.356472	-1.344014
C	-0.851806	-1.546833	0.139731
C	-1.331264	-0.762396	1.189821
C	-1.658886	-1.747729	-0.985375
C	-2.584225	-0.157081	1.099130
H	-0.705341	-0.618938	2.061703
C	-2.912693	-1.145742	-1.077349
H	-1.301039	-2.375715	-1.799447
C	-3.376455	-0.340978	-0.034888
H	-2.938454	0.467186	1.914534
H	-3.527816	-1.307028	-1.958590
H	-4.351131	0.134081	-0.104214

C	0.992716	1.009097	-0.175351
C	0.908980	1.780296	0.986169
C	0.103251	1.262769	-1.225114
C	-0.063086	2.773297	1.106591
H	1.598265	1.578220	1.797810
C	-0.875292	2.246495	-1.102038
H	0.147770	0.673010	-2.135697
C	-0.963176	3.004979	0.066446
H	-0.123306	3.358869	2.019989
H	-1.575026	2.413145	-1.915737
H	-1.729453	3.768713	0.164665
O	2.727058	-0.295490	0.895006
O	1.054465	-2.155391	1.490365
H	1.990098	-1.877575	1.460191

18

B3LYP SCF energy: -385.43898878 a.u.

B3LYP enthalpy: -385.282351 a.u.

B3LYP free energy: -385.325637 a.u.

B3LYP SCF energy in acetonitrile solution: -385.57441983 a.u.

B3LYP enthalpy in acetonitrile solution: -385.417782 a.u.

B3LYP free energy in acetonitrile solution: -385.461068 a.u.

Cartesian coordinates

ATOM	X	Y	Z
C	1.696162	0.065175	-0.360492
H	1.917682	0.151685	-1.433036
C	2.356356	1.184766	0.374938
H	3.397556	1.411414	0.170318
H	1.888784	1.634745	1.245177
C	0.184463	0.032777	-0.174917
C	-0.488450	-1.184344	-0.029793
C	-0.557617	1.220594	-0.179586
C	-1.875611	-1.213622	0.122032
H	0.085766	-2.104517	-0.041272
C	-1.944313	1.192591	-0.033231
H	-0.045937	2.171816	-0.301354
C	-2.608045	-0.026018	0.122280
H	-2.384418	-2.166817	0.238016
H	-2.506020	2.122755	-0.037871
H	-3.687794	-0.048808	0.240245
O	2.315865	-1.190470	-0.010225
H	2.229790	-1.280031	0.954188

^{1}TS-1

B3LYP SCF energy: -770.86834913 a.u.

B3LYP enthalpy: -770.555048 a.u.

B3LYP free energy: -770.619397 a.u.

B3LYP SCF energy in acetonitrile solution: -771.11748350 a.u.

B3LYP enthalpy in acetonitrile solution: -770.804182 a.u.

B3LYP free energy in acetonitrile solution: -770.868531 a.u.

B3LYP SCF energy in 1, 2-dichloroethane solution: -771.11587336 a.u.

B3LYP enthalpy in 1, 2-dichloroethane solution: -770.802572 a.u.

B3LYP free energy in 1, 2-dichloroethane solution: -770.866920 a.u.

Imaginary frequency: -1866.0608 cm^{-1}

Cartesian coordinates

ATOM	X	Y	Z
C	0.275752	-2.266952	-0.628207
H	0.899627	-2.623791	0.196726
H	0.061787	-3.137380	-1.260291
C	1.111201	-1.276505	-1.487004
H	1.960104	-1.840223	-1.889377
H	0.512603	-0.970458	-2.357204
C	1.662890	-0.044358	-0.777487
H	1.907455	-0.497198	0.552217
C	3.111715	0.258663	-1.131221
H	3.193640	0.458314	-2.210519

H	3.497806	1.135529	-0.606050
H	3.755309	-0.596995	-0.900772
C	-1.019528	-1.702261	-0.096636
C	-1.087996	-1.116084	1.172949
C	-2.169447	-1.700403	-0.897104
C	-2.264564	-0.513239	1.617053
H	-0.208566	-1.117962	1.810020
C	-3.348162	-1.099095	-0.457315
H	-2.134350	-2.166266	-1.879964
C	-3.396099	-0.495019	0.800871
H	-2.294668	-0.052024	2.600183
H	-4.229129	-1.104749	-1.093650
H	-4.311586	-0.022322	1.145357
C	0.783233	1.114295	-0.510142
C	1.207360	2.118293	0.389431
C	-0.496736	1.252638	-1.082222
C	0.390038	3.196840	0.706004
H	2.174641	2.025249	0.874192
C	-1.312213	2.335268	-0.769737
H	-0.864517	0.504385	-1.770993
C	-0.877197	3.312263	0.127441
H	0.737127	3.945202	1.412844
H	-2.296044	2.408771	-1.223683
H	-1.518933	4.152044	0.377827

O 2.256429 -0.868498 1.615442

O 3.210598 -1.724776 1.429944

^{3}TS-1

B3LYP SCF energy: -770.87309778 a.u.

B3LYP enthalpy: -770.559578 a.u.

B3LYP free energy: -770.624975 a.u.

B3LYP SCF energy in acetonitrile solution: -771.12222498 a.u.

B3LYP enthalpy in acetonitrile solution: -770.808705 a.u.

B3LYP free energy in acetonitrile solution: -770.874102 a.u.

Imaginary frequency: -1386.6546 cm^{-1}

Cartesian coordinates

ATOM	X	Y	Z
C	-0.102016	-2.276301	0.645365
H	-0.708549	-2.671095	-0.175288
H	0.166197	-3.131765	1.277273
C	-0.986612	-1.334189	1.510509
H	-1.780155	-1.952971	1.943937
H	-0.388819	-0.972673	2.359512
C	-1.648456	-0.158299	0.800835
H	-1.849304	-0.643609	-0.594866

C	-3.128791	-0.019598	1.116031
H	-3.261935	0.143355	2.196589
H	-3.597435	0.820715	0.599171
H	-3.670737	-0.934226	0.851047
C	1.156076	-1.638238	0.107817
C	1.192096	-1.061639	-1.167214
C	2.301574	-1.557461	0.911010
C	2.331173	-0.392591	-1.614924
H	0.317049	-1.123882	-1.807130
C	3.442282	-0.889128	0.468039
H	2.292334	-2.014763	1.898444
C	3.456989	-0.296442	-0.796531
H	2.335946	0.059005	-2.602960
H	4.319938	-0.834101	1.106567
H	4.342884	0.227696	-1.143908
C	-0.886002	1.071650	0.520684
C	-1.429129	2.060702	-0.332934
C	0.408699	1.299557	1.032046
C	-0.714549	3.206729	-0.659194
H	-2.408628	1.906172	-0.774762
C	1.121055	2.449283	0.708691
H	0.868772	0.568472	1.682298
C	0.567442	3.409767	-0.139696
H	-1.154096	3.940955	-1.328472

H	2.117871	2.588804	1.116585
H	1.128257	4.303269	-0.398220
O	-2.141602	-1.014853	-1.631492
O	-3.053470	-1.925418	-1.477135

TS-2

B3LYP SCF energy: -696.34621039 a.u.

B3LYP enthalpy: -696.022662 a.u.

B3LYP free energy: -696.083964 a.u.

B3LYP SCF energy in acetonitrile solution: -696.57475634 a.u.

B3LYP enthalpy in acetonitrile solution: -696.251208 a.u.

B3LYP free energy in acetonitrile solution: -696.312510 a.u.

Imaginary frequency: -26.9814 cm^{-1}

Cartesian coordinates

ATOM	X	Y	Z
C	0.143990	-2.418846	0.087380
H	0.599406	-2.544158	1.075492
H	-0.145827	-3.417497	-0.263732
C	1.224880	-1.879066	-0.875296
H	1.972243	-2.670199	-1.012878
H	0.786064	-1.704940	-1.867175

C	1.975187	-0.621227	-0.407739
C	3.261193	-0.405706	-1.223054
H	3.011518	-0.211098	-2.273965
H	3.822218	0.456543	-0.849509
H	3.908350	-1.288743	-1.180945
C	-1.089281	-1.552002	0.194313
C	-1.299166	-0.723182	1.301166
C	-2.028905	-1.523396	-0.844120
C	-2.390703	0.142514	1.355160
H	-0.588932	-0.753313	2.122488
C	-3.126307	-0.665295	-0.795082
H	-1.889109	-2.172970	-1.705830
C	-3.305480	0.179372	0.302440
H	-2.523925	0.790892	2.216566
H	-3.842205	-0.654765	-1.612739
H	-4.155419	0.854871	0.339826
C	1.169509	0.665563	-0.351277
C	1.453851	1.614442	0.641474
C	0.177301	0.971075	-1.290275
C	0.741929	2.811102	0.722257
H	2.243863	1.407833	1.359867
C	-0.540178	2.163197	-1.211165
H	-0.064326	0.261697	-2.073300
C	-0.266969	3.086236	-0.200897

H	0.973609	3.524713	1.508392
H	-1.324670	2.363791	-1.935129
H	-0.833980	4.010427	-0.135039
H	2.309174	-0.842357	0.645282
O	2.203250	-1.019182	2.351475
H	1.510838	-0.325959	2.398825

TS-3

B3LYP SCF energy: -770.92123052 a.u.

B3LYP enthalpy: -770.606482 a.u.

B3LYP free energy: -770.670062 a.u.

B3LYP SCF energy in acetonitrile solution: -771.25740327 a.u.

B3LYP enthalpy in acetonitrile solution: -770.942655 a.u.

B3LYP free energy in acetonitrile solution: -771.006235 a.u.

Imaginary frequency: -163.5607 cm^{-1}

Cartesian coordinates

ATOM	X	Y	Z
C	-1.245454	-1.710415	-0.991968
H	-1.005268	-2.491674	-0.261010
H	-1.878179	-2.159376	-1.772763
C	0.085533	-1.245307	-1.665663

H	0.362026	-2.047766	-2.367397
H	-0.137802	-0.367156	-2.289454
C	1.279026	-0.989549	-0.763144
H	0.695047	-1.546109	0.917080
C	2.250420	-2.141336	-0.734211
H	2.630724	-2.397347	-1.741423
H	3.115888	-1.959566	-0.090730
H	1.755697	-3.044975	-0.332607
C	-2.011394	-0.579059	-0.351120
C	-1.867026	-0.269574	1.008843
C	-2.846156	0.236429	-1.130083
C	-2.523208	0.830938	1.561332
H	-1.219435	-0.887633	1.623824
C	-3.503303	1.337440	-0.580765
H	-2.969596	0.006751	-2.187146
C	-3.340690	1.641973	0.772325
H	-2.386119	1.060676	2.615478
H	-4.140719	1.957693	-1.207854
H	-3.845493	2.502546	1.205595
C	1.656545	0.348611	-0.417114
C	2.932156	0.684382	0.138713
C	0.767529	1.459233	-0.585184
C	3.273656	1.983479	0.485731
H	3.665435	-0.100259	0.293003

C	1.122398	2.754121	-0.235986
H	-0.229832	1.286259	-0.970469
C	2.380513	3.047852	0.304979
H	4.262646	2.172801	0.903085
H	0.394864	3.551973	-0.381881
H	2.652268	4.063658	0.581555
O	0.643030	-2.109142	1.764264
O	0.333791	-3.382834	1.306111

TS-4

B3LYP SCF energy: -770.89615822 a.u.

B3LYP enthalpy: -770.580373 a.u.

B3LYP free energy: -770.644447 a.u.

B3LYP SCF energy in acetonitrile solution: -771.15160283 a.u.

B3LYP enthalpy in acetonitrile solution: -770.835818 a.u.

B3LYP free energy in acetonitrile solution: -770.899892 a.u.

Imaginary frequency: -168.3920 cm^{-1}

Cartesian coordinates

ATOM	X	Y	Z
C	-0.619808	-2.048584	-0.408783
H	-1.020752	-1.852224	-1.410410

H	-0.730504	-3.126295	-0.233732
C	-1.501934	-1.316471	0.625033
H	-2.470446	-1.822907	0.683469
H	-1.038689	-1.371671	1.616026
C	-1.818864	0.154283	0.261202
C	-2.825722	0.739834	1.324183
H	-2.323097	0.726779	2.295087
H	-3.079513	1.768227	1.060712
H	-3.726956	0.125331	1.349195
C	0.846771	-1.684197	-0.353774
C	1.435831	-0.879791	-1.334169
C	1.637470	-2.114823	0.719430
C	2.769791	-0.487261	-1.231802
H	0.833644	-0.531702	-2.168474
C	2.973268	-1.730168	0.825101
H	1.197285	-2.751362	1.484400
C	3.542826	-0.908219	-0.149276
H	3.201722	0.155277	-1.993686
H	3.570008	-2.072403	1.666484
H	4.581748	-0.601446	-0.066670
C	-0.601912	1.091398	0.218440
C	-0.429380	1.977654	-0.847503
C	0.346430	1.071429	1.246830
C	0.688794	2.810960	-0.899206

H	-1.168436	1.998515	-1.640201
C	1.466909	1.896999	1.191400
H	0.232415	0.389346	2.082954
C	1.644405	2.768534	0.115547
H	0.815719	3.487713	-1.739922
H	2.206150	1.851306	1.985631
H	2.521502	3.407781	0.069724
O	-2.530487	0.211632	-0.911104
O	-4.568743	-1.045874	-0.521844
H	-4.137211	-1.155793	-1.392915

TS-5

B3LYP SCF energy: -846.66840727 a.u.

B3LYP enthalpy: -846.334635 a.u.

B3LYP free energy: -846.403317 a.u.

B3LYP SCF energy in acetonitrile solution: -846.95732661 a.u.

B3LYP enthalpy in acetonitrile solution: -846.623554 a.u.

B3LYP free energy in acetonitrile solution: -846.692236 a.u.

B3LYP SCF energy in 1, 2-dichloroethane solution: -846.95604022 a.u.

B3LYP enthalpy in 1, 2-dichloroethane solution: -846.622268 a.u.

B3LYP free energy in 1, 2-dichloroethane solution: -846.690950 a.u.

Imaginary frequency: -746.1247 cm^{-1}

Cartesian coordinates

ATOM	X	Y	Z
C	-0.688592	-1.980421	-0.012138
H	-1.191366	-1.925263	-0.982019
H	-0.882173	-2.981077	0.395435
C	-1.351444	-0.961036	0.935947
H	-2.378330	-1.290202	1.128548
H	-0.843387	-0.960588	1.906211
C	-1.455980	0.487284	0.401298
C	-2.393133	1.315809	1.298519
H	-1.974525	1.401921	2.306558
H	-2.501782	2.325761	0.890140
H	-3.376686	0.841440	1.367397
C	0.800513	-1.795740	-0.191620
C	1.312730	-1.110012	-1.298545
C	1.695096	-2.257243	0.781249
C	2.679548	-0.863559	-1.416091
H	0.625396	-0.736120	-2.051470
C	3.064399	-2.017899	0.666979
H	1.311625	-2.801048	1.642461
C	3.560401	-1.313143	-0.431074
H	3.055610	-0.310895	-2.272378
H	3.743635	-2.381485	1.433666

H	4.625688	-1.118922	-0.520031
C	-0.108385	1.222840	0.287506
C	0.118168	2.121036	-0.762864
C	0.891322	1.051255	1.251469
C	1.333022	2.798992	-0.869121
H	-0.653299	2.275259	-1.508310
C	2.102464	1.730731	1.147257
H	0.744629	0.364709	2.076688
C	2.333062	2.601970	0.081517
H	1.495339	3.480802	-1.699545
H	2.872406	1.566346	1.895390
H	3.282494	3.122918	-0.003824
O	-1.932844	0.462808	-0.938781
O	-3.502100	-0.270216	-0.938857
H	-3.950188	0.579508	-1.095619
O	-4.910981	-0.917694	-0.113534
H	-4.596826	-1.837422	-0.201666

TS-6

B3LYP SCF energy: -695.14666286 a.u.

B3LYP enthalpy: -694.846322 a.u.

B3LYP free energy: -694.905666 a.u.

B3LYP SCF energy in acetonitrile solution: -695.36825350 a.u.

B3LYP enthalpy in acetonitrile solution: -695.067913 a.u.

B3LYP free energy in acetonitrile solution: -695.127257 a.u.

Imaginary frequency: -431.4138 cm^{-1}

Cartesian coordinates

ATOM	X	Y	Z
C	-0.247292	-1.936968	-1.025876
H	-0.642281	-1.397826	-1.894661
H	-0.063200	-2.966592	-1.377345
C	-1.320940	-2.011536	0.027336
H	-2.139466	-2.696017	-0.178989
H	-0.995735	-2.013850	1.065685
C	-2.435996	-0.301522	0.032491
C	-3.278069	-0.568152	1.286361
H	-2.683943	-0.718825	2.191278
H	-3.923202	0.303411	1.451415
H	-3.916791	-1.438842	1.118678
C	1.062014	-1.329971	-0.568880
C	1.651271	-0.278234	-1.275038
C	1.717051	-1.821030	0.568736
C	2.851436	0.287681	-0.845513
H	1.149727	0.119474	-2.151606
C	2.916487	-1.259139	1.002619

H	1.284342	-2.652187	1.121273
C	3.486889	-0.197549	0.297214
H	3.283057	1.116515	-1.399499
H	3.407699	-1.651648	1.888963
H	4.419766	0.245017	0.635109
C	-1.355789	0.753591	0.159474
C	-1.166953	1.632294	-0.914239
C	-0.553421	0.894098	1.299614
C	-0.201186	2.634570	-0.848413
H	-1.795534	1.511907	-1.789893
C	0.423657	1.884824	1.360076
H	-0.669312	0.212852	2.136868
C	0.600664	2.760347	0.287159
H	-0.071448	3.316170	-1.684866
H	1.055263	1.966114	2.239717
H	1.362550	3.533213	0.335853
O	-2.995054	-0.456089	-1.095837

TS-7

B3LYP SCF energy: -461.75949482 a.u.

B3LYP enthalpy: -461.577515 a.u.

B3LYP free energy: -461.627926 a.u.

B3LYP SCF energy in acetonitrile solution: -461.93767734 a.u.

B3LYP enthalpy in acetonitrile solution: -461.755698 a.u.

B3LYP free energy in acetonitrile solution: -461.806109 a.u.

Imaginary frequency: -630.9443 cm^{-1}

Cartesian coordinates

ATOM	X	Y	Z
C	-0.555214	1.636573	-0.756101
H	-1.068528	1.326130	-1.673386
C	-1.559553	1.749558	0.349723
H	-2.508377	2.234592	0.148757
H	-1.205513	1.787599	1.376537
C	0.567001	0.672263	-0.428785
C	1.696944	1.105617	0.275235
C	0.469082	-0.682928	-0.775107
C	2.706434	0.209499	0.627955
H	1.786723	2.155332	0.545780
C	1.477967	-1.581720	-0.422845
H	-0.401652	-1.031307	-1.323605
C	2.598788	-1.138517	0.280723
H	3.578098	0.564229	1.171121
H	1.387018	-2.627778	-0.701607
H	3.384639	-1.837137	0.553378
O	-2.384741	-0.214807	0.394438

H	-1.531226	-0.588857	0.676515
O	-3.092205	-1.658564	0.178839
H	-3.887122	-1.450994	0.700595
H	-0.127181	2.633087	-0.965085

TS-8

B3LYP SCF energy: -771.49171876 a.u.

B3LYP enthalpy: -771.166290 a.u.

B3LYP free energy: -771.231259 a.u.

B3LYP SCF energy in acetonitrile solution: -771.75092293 a.u.

B3LYP enthalpy in acetonitrile solution: -771.425494 a.u.

B3LYP free energy in acetonitrile solution: -771.490463 a.u.

Imaginary frequency: -1772.8540 cm^{-1}

Cartesian coordinates

ATOM	X	Y	Z
C	0.612348	-2.179732	-0.604848
H	1.271363	-2.425950	0.234545
H	0.525078	-3.086257	-1.216250
C	1.310398	-1.098715	-1.470347
H	2.251189	-1.527735	-1.834750
H	0.700362	-0.909913	-2.365616

C	1.632671	0.219113	-0.772694
C	2.934067	0.855324	-1.244728
H	2.860993	1.108314	-2.313078
H	3.151602	1.778210	-0.700638
H	3.774667	0.166245	-1.113201
C	-0.747732	-1.788920	-0.078385
C	-0.883955	-1.176935	1.173570
C	-1.891458	-1.974658	-0.864029
C	-2.128964	-0.730571	1.613891
H	-0.000951	-1.025998	1.787566
C	-3.139277	-1.531948	-0.425740
H	-1.799581	-2.460232	-1.833615
C	-3.259613	-0.900332	0.813419
H	-2.213988	-0.241147	2.579961
H	-4.016525	-1.680706	-1.049774
H	-4.229123	-0.548587	1.154943
C	0.528215	1.183915	-0.497306
C	0.703773	2.142934	0.518934
C	-0.691885	1.175033	-1.192147
C	-0.306882	3.044175	0.838400
H	1.628736	2.143765	1.088613
C	-1.701528	2.080781	-0.877297
H	-0.864991	0.447307	-1.975063
C	-1.517195	3.016628	0.141292

H	-0.154392	3.762842	1.638857
H	-2.639605	2.045925	-1.423705
H	-2.310212	3.715060	0.393129
O	2.426794	-0.432101	1.556349
O	3.585211	-1.196346	1.281798
H	3.250072	-2.109311	1.362227
H	2.001370	-0.140799	0.448758

TS-9

B3LYP SCF energy: -695.14173086 a.u.

B3LYP enthalpy: -694.841555 a.u.

B3LYP free energy: -694.901807 a.u.

B3LYP SCF energy in acetonitrile solution: -695.36194593 a.u.

B3LYP enthalpy in acetonitrile solution: -695.061770 a.u.

B3LYP free energy in acetonitrile solution: -695.122022 a.u.

Imaginary frequency: -381.8848 cm^{-1}

Cartesian coordinates

ATOM	X	Y	Z
C	-0.145921	-2.532643	0.275888
H	0.166590	-2.751830	1.302456
H	-0.478681	-3.475701	-0.174549

C	1.103166	-2.045815	-0.502842
H	1.840064	-2.854312	-0.510081
H	0.832341	-1.828041	-1.540009
C	1.749339	-0.842223	0.203331
C	3.449905	-0.630724	-1.008351
H	3.011318	-0.486500	-1.990932
H	3.905809	0.241486	-0.553752
H	3.942551	-1.579839	-0.825461
C	-1.275373	-1.530800	0.270936
C	-1.418582	-0.607268	1.312940
C	-2.145984	-1.446603	-0.822288
C	-2.387869	0.392839	1.252449
H	-0.741537	-0.656687	2.161407
C	-3.120982	-0.451359	-0.885709
H	-2.050315	-2.162850	-1.635958
C	-3.239416	0.477092	0.150063
H	-2.471288	1.112119	2.062139
H	-3.787058	-0.399719	-1.742924
H	-3.993078	1.257953	0.100137
C	1.193968	0.544273	-0.053884
C	1.416901	1.526281	0.920720
C	0.437708	0.872353	-1.186322
C	0.877726	2.801393	0.778295
H	1.999933	1.254326	1.793953

C	-0.109676	2.145986	-1.325577
H	0.243519	0.129633	-1.951528
C	0.106453	3.114314	-0.343982
H	1.049370	3.550254	1.546801
H	-0.713013	2.378724	-2.198239
H	-0.324856	4.105623	-0.451836
O	2.327520	-1.069652	1.299297

TS-10

B3LYP SCF energy: -770.28734337 a.u.

B3LYP enthalpy: -769.984932 a.u.

B3LYP free energy: -770.044972 a.u.

B3LYP SCF energy in acetonitrile solution: -770.53450155 a.u.

B3LYP enthalpy in acetonitrile solution: -770.232090 a.u.

B3LYP free energy in acetonitrile solution: -770.292130 a.u.

Imaginary frequency: -1775.1600 cm^{-1}

Cartesian coordinates

ATOM	X	Y	Z
C	1.110598	-1.691663	0.227374
H	0.542759	-1.723548	-0.956388
H	1.597851	-2.669029	0.248568

C	-0.149377	-1.639335	1.085077
H	-0.273156	-2.611371	1.571237
H	-0.044096	-0.890692	1.876260
C	-1.485199	-1.359311	0.307287
C	-2.670925	-1.981651	1.056267
H	-2.747711	-1.557507	2.061364
H	-3.602302	-1.775933	0.521922
H	-2.537853	-3.064871	1.138122
C	2.069094	-0.580788	0.177765
C	1.687611	0.762845	0.366607
C	3.420602	-0.850222	-0.123426
C	2.624721	1.787111	0.272834
H	0.652658	1.005006	0.574609
C	4.355541	0.175071	-0.215153
H	3.730495	-1.880663	-0.279393
C	3.962000	1.501583	-0.016056
H	2.307863	2.815592	0.422009
H	5.392534	-0.058245	-0.439911
H	4.690071	2.304457	-0.087801
C	-1.750347	0.125840	0.073774
C	-2.024700	0.639431	-1.197656
C	-1.796333	0.995978	1.172607
C	-2.307823	1.995764	-1.366260
H	-1.996483	-0.018284	-2.055685

C -2.080767 2.350736 1.005049

H -1.607100 0.618099 2.173975

C -2.332046 2.858127 -0.270400

H -2.507303 2.378050 -2.363483

H -2.107044 3.006313 1.871027

H -2.548830 3.913760 -0.406932

O -1.440879 -2.124709 -0.908562

O -0.401402 -1.666321 -1.741894

TS-11

B3LYP SCF energy: -770.26877295 a.u.

B3LYP enthalpy: -769.963656 a.u.

B3LYP free energy: -770.024301 a.u.

B3LYP SCF energy in acetonitrile solution: -770.52428938 a.u.

B3LYP enthalpy in acetonitrile solution: -770.219172 a.u.

B3LYP free energy in acetonitrile solution: -770.279817 a.u.

Imaginary frequency: -1408.6630 cm^{-1}

Cartesian coordinates

ATOM X Y Z

C -0.841838 -1.777443 -0.361087

H -1.207964 -2.788322 -0.203400

C	0.487472	-1.685225	-1.086745
H	0.726930	-2.683959	-1.468455
H	0.388115	-1.028121	-1.959296
C	1.727051	-1.215068	-0.254405
C	3.034269	-1.575635	-0.992279
H	3.095406	-1.067207	-1.958775
H	3.891623	-1.267645	-0.387566
H	3.084992	-2.656092	-1.157301
C	-1.842794	-0.760330	-0.290885
C	-1.613626	0.590093	-0.655623
C	-3.140964	-1.103895	0.172849
C	-2.625686	1.535565	-0.552282
H	-0.636581	0.894455	-1.004976
C	-4.145795	-0.154592	0.271709
H	-3.339683	-2.134699	0.457082
C	-3.895612	1.176251	-0.089145
H	-2.420506	2.565176	-0.832283
H	-5.129992	-0.445395	0.628724
H	-4.681895	1.921290	-0.010282
C	1.685505	0.292202	0.004070
C	1.545896	0.799626	1.298103
C	1.791535	1.195386	-1.063886
C	1.482537	2.177036	1.515688
H	1.466317	0.107627	2.126105

C	1.733081	2.571625	-0.847963
H	1.906523	0.826119	-2.079922
C	1.571047	3.068469	0.446743
H	1.359467	2.553248	2.527679
H	1.813445	3.253696	-1.690117
H	1.519217	4.139854	0.619182
O	1.823097	-1.974736	0.936927
O	0.216167	-1.806653	1.504839
H	0.138006	-2.743301	1.770287

TS-12

B3LYP SCF energy: -770.36269070 a.u.

B3LYP enthalpy: -770.056870 a.u.

B3LYP free energy: -770.116927 a.u.

B3LYP SCF energy in acetonitrile solution: -770.61744551 a.u.

B3LYP enthalpy in acetonitrile solution: -770.311625 a.u.

B3LYP free energy in acetonitrile solution: -770.371682 a.u.

Imaginary frequency: -374.8939 cm^{-1}

Cartesian coordinates

ATOM	X	Y	Z
C	-0.477380	-2.079815	-0.378147

H	-0.339062	-3.169137	-0.221008
C	-1.491839	-1.694759	0.674265
H	-2.419956	-2.260110	0.639052
H	-1.111031	-1.518270	1.677600
C	-2.320712	0.126199	0.234207
C	-3.202365	0.284788	1.475224
H	-2.635095	0.289285	2.409057
H	-3.720955	1.247825	1.393926
H	-3.953342	-0.508169	1.501437
C	0.896801	-1.450998	-0.168930
C	1.500384	-0.695010	-1.172939
C	1.585028	-1.661923	1.032310
C	2.756745	-0.125097	-0.965887
H	0.969105	-0.555326	-2.106373
C	2.841268	-1.095724	1.240805
H	1.139645	-2.278183	1.811182
C	3.429635	-0.318557	0.240810
H	3.209351	0.475029	-1.750401
H	3.362806	-1.265587	2.178964
H	4.408656	0.125204	0.399157
C	-1.119925	1.036579	0.150982
C	-0.876833	1.696453	-1.060672
C	-0.265375	1.270979	1.236660
C	0.196697	2.574598	-1.184417

H	-1.547131	1.512586	-1.893142
C	0.819235	2.133593	1.107696
H	-0.425827	0.756715	2.179051
C	1.051589	2.790025	-0.102603
H	0.369942	3.086722	-2.126739
H	1.491196	2.283878	1.947054
H	1.898427	3.463040	-0.201332
O	-2.900042	-0.215071	-0.851618
O	-0.971664	-1.934805	-1.688812
H	-1.660802	-1.234478	-1.670218

H_2O

B3LYP SCF energy: -76.40759806 a.u.

B3LYP enthalpy: -76.382681 a.u.

B3LYP free energy: -76.404126 a.u.

B3LYP SCF energy in acetonitrile solution: -76.46787010 a.u.

B3LYP enthalpy in acetonitrile solution: -76.442953 a.u.

B3LYP free energy in acetonitrile solution: -76.464398 a.u.

Cartesian coordinates

ATOM	X	Y	Z
O	0.000000	0.000000	0.119426

H 0.000000 -0.762602 -0.477706

H 0.000000 0.762602 -0.477706

H_2O_2

B3LYP SCF energy: -151.53111183 a.u.

B3LYP enthalpy: -151.500693 a.u.

B3LYP free energy: -151.527205 a.u.

B3LYP SCF energy in acetonitrile solution: -151.61483526 a.u.

B3LYP enthalpy in acetonitrile solution: -151.584416 a.u.

B3LYP free energy in acetonitrile solution: -151.610928 a.u.

B3LYP SCF energy in 1, 2-dichloroethane solution: -151.61249391 a.u.

B3LYP enthalpy in 1, 2-dichloroethane solution: -151.582075 a.u.

B3LYP free energy in 1, 2-dichloroethane solution: -151.608587 a.u.

Cartesian coordinates

ATOM	X	Y	Z
O	-0.718361	-0.117101	-0.055591
H	-1.012065	0.664961	0.444715
O	0.718362	0.117091	-0.055605
H	1.012060	-0.664876	0.444856

OOH

B3LYP SCF energy: -150.89667994 a.u.

B3LYP enthalpy: -150.878864 a.u.

B3LYP free energy: -150.904857 a.u.

B3LYP SCF energy in acetonitrile solution: -150.96941660 a.u.

B3LYP enthalpy in acetonitrile solution: -150.951601 a.u.

B3LYP free energy in acetonitrile solution: -150.977594 a.u.

B3LYP SCF energy in 1, 2-dichloroethane solution: -150.96650581 a.u.

B3LYP enthalpy in 1, 2-dichloroethane solution: -150.948690 a.u.

B3LYP free energy in 1, 2-dichloroethane solution: -150.974683 a.u.

Cartesian coordinates

ATOM	X	Y	Z
O	0.055904	-0.611664	0.000000
H	-0.894471	-0.866118	0.000000
O	0.055904	0.719929	0.000000

OH

B3LYP SCF energy: -75.72178850 a.u.

B3LYP enthalpy: -75.710222 a.u.

B3LYP free energy: -75.730470 a.u.

B3LYP SCF energy in acetonitrile solution: -75.76875804 a.u.

B3LYP enthalpy in acetonitrile solution: -75.757192 a.u.

B3LYP free energy in acetonitrile solution: -75.777439 a.u.

B3LYP SCF energy in 1, 2-dichloroethane solution: -75.76781890 a.u.

B3LYP enthalpy in 1, 2-dichloroethane solution: -75.756253 a.u.

B3LYP free energy in 1, 2-dichloroethane solution: -75.776500 a.u.

Cartesian coordinates

ATOM	X	Y	Z
O	0.000000	0.000000	0.109255
H	0.000000	0.000000	-0.874036

$^1O_2$

B3LYP SCF energy: -150.30044256 a.u.

B3LYP enthalpy: -150.293375 a.u.

B3LYP free energy: -150.315624 a.u.

B3LYP SCF energy in acetonitrile solution: -150.31362682 a.u.

B3LYP enthalpy in acetonitrile solution: -150.306559 a.u.

B3LYP free energy in acetonitrile solution: -150.328809 a.u.

B3LYP SCF energy in 1, 2-dichloroethane solution: -150.31048074 a.u.

B3LYP enthalpy in 1, 2-dichloroethane solution: -150.303413 a.u.

B3LYP free energy in 1, 2-dichloroethane solution: -150.325663 a.u.

Cartesian coordinates

ATOM	X	Y	Z
O	0.000000	0.000000	0.607424
O	0.000000	0.000000	-0.607424

$^3O_2$

B3LYP SCF energy: -150.31705884 a.u.

B3LYP enthalpy: -150.309978 a.u.

B3LYP free energy: -150.333263 a.u.

B3LYP SCF energy in acetonitrile solution: -150.37464366 a.u.

B3LYP enthalpy in acetonitrile solution: -150.367563 a.u.

B3LYP free energy in acetonitrile solution: -150.390848 a.u.

Cartesian coordinates

ATOM	X	Y	Z
O	0.000000	0.000000	0.607116
O	0.000000	0.000000	-0.607116

O_2 radical anion

B3LYP SCF energy: -150.29554561 a.u.

B3LYP enthalpy: -150.289488 a.u.

B3LYP free energy: -150.312615 a.u.

B3LYP SCF energy in acetonitrile solution: -150.49888663 a.u.

B3LYP enthalpy in acetonitrile solution: -150.492829 a.u.

B3LYP free energy in acetonitrile solution: -150.515956 a.u.

Cartesian coordinates

ATOM	X	Y	Z
O	0.000000	0.000000	0.677536
O	0.000000	0.000000	-0.677536

6. Analytical data of products

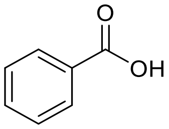

Benzoic acid (2)[16]

^{1}H NMR (400 MHz, Chloroform-*d*) δ 11.72 (b, 1H), 8.14 (d, *J* = 6.8 Hz, 2H), 7.63 (t, *J* = 7.4 Hz, 1H), 7.49 (t, *J* = 7.8 Hz, 2H).

^{13}C NMR (101 MHz, Chloroform-*d*) δ 172.33, 133.81, 130.21, 129.30, 128.48.

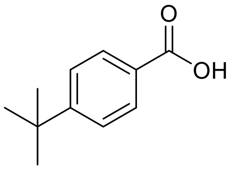

4-(*tert*-Butyl)benzoic acid (4)[17]

^{1}H NMR (400 MHz, Chloroform-*d*) δ 8.06 (d, *J* = 7.6 Hz, 2H), 7.50 (d, *J* = 7.7 Hz, 2H), 1.36 (s, 9H).

^{13}C NMR (101 MHz, Chloroform-*d*) δ 172.40, 157.58, 130.11, 126.53, 125.46, 35.18, 31.09.

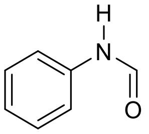

***N*-Phenylformamide (6a)**[18]

^{1}H NMR (400 MHz, Chloroform-*d*) δ 9.07 (d, *J* = 8.0 Hz, 0.5H), 8.69 (d, *J* = 11.3 Hz, 0.5H), 8.49 – 8.24 (m, 1H), 7.55 (m, 1H), 7.40 – 7.25 (m, 2H), 7.22 – 7.05 (m, 2H).

^{13}C NMR (101 MHz, Chloroform-*d*) δ 163.03, 159.59, 136.92, 136.70, 129.58, 128.90, 125.12, 124.62, 120.04, 118.64.

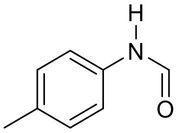

***N*-*p*-Tolylformamide (6b)**[19]

^{1}H NMR (400 MHz, Chloroform-*d*) δ 8.91 (d, *J* = 10.9 Hz, 0.5H), 8.62 (d, *J* = 11.4 Hz, 0.5H), 8.29 (d, *J* = 1.9 Hz, 0.5H), 8.26 – 8.12 (m, 0.5H), 7.43 (d, *J* = 8.2 Hz, 1H), 7.19 – 7.05 (m, 2H), 6.99 (m, 1H), 2.37 – 2.24 (m, 3H).

^{13}C NMR (101 MHz, Chloroform-*d*) δ 163.08, 159.42, 134.93, 134.37, 134.23, 134.10, 130.06, 129.37, 120.06, 118.94, 20.75, 20.66.

7. NMR spectra of products

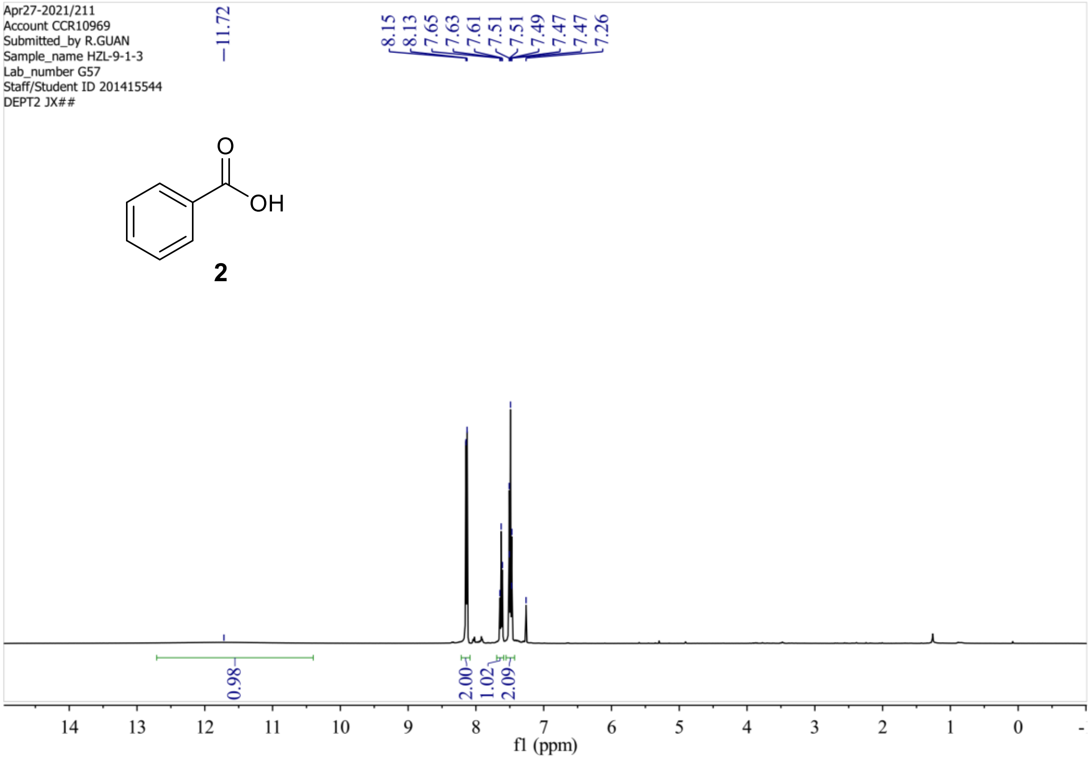

^{1}H NMR

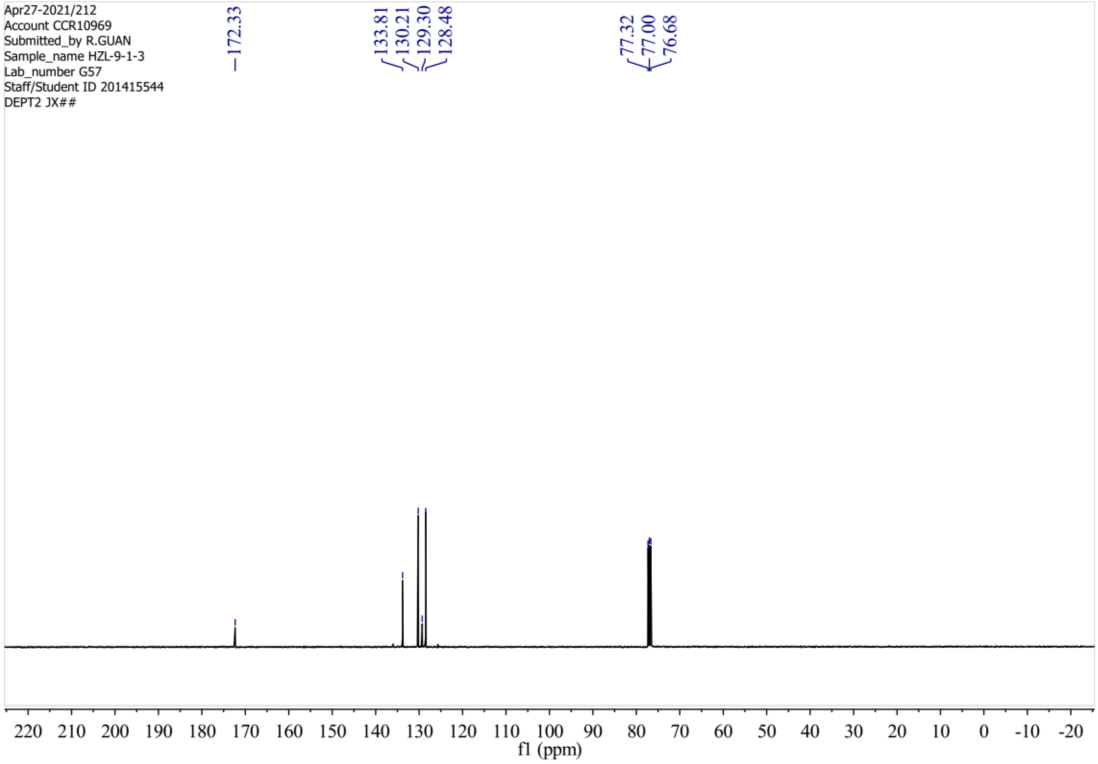

^{13}C NMR

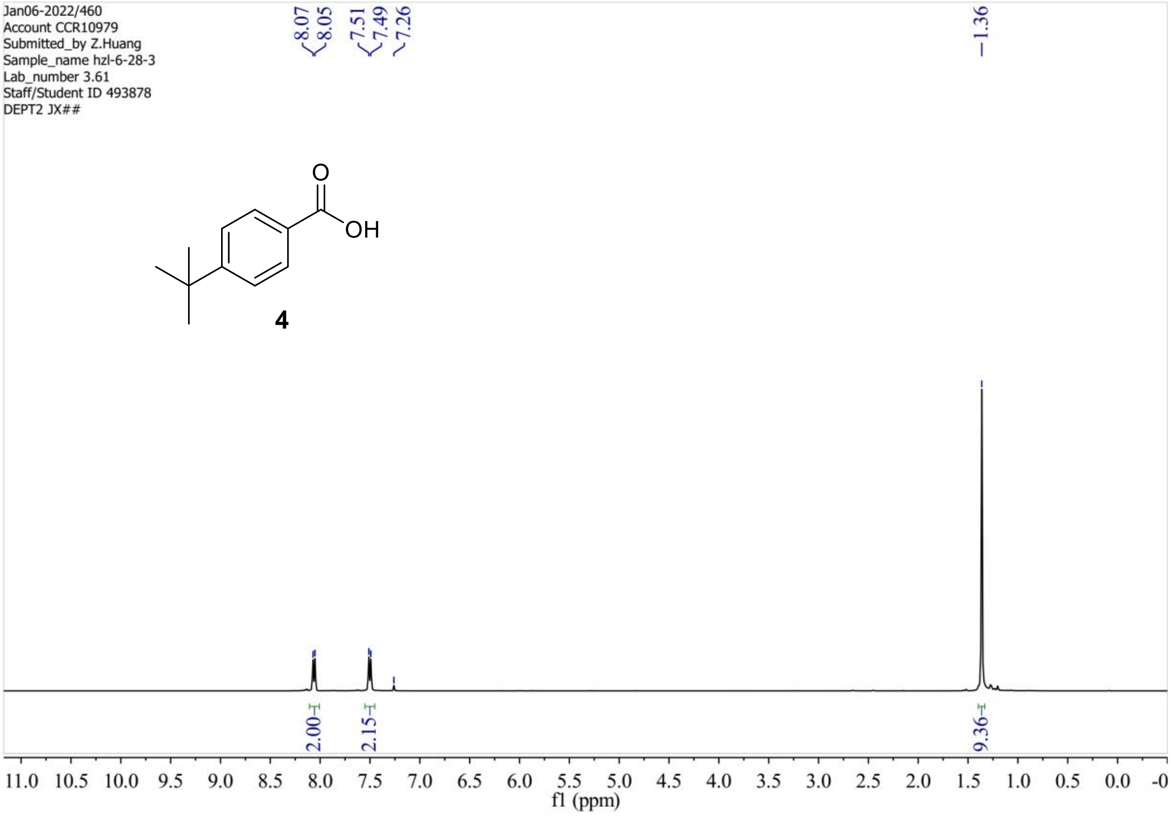

^{1}H NMR

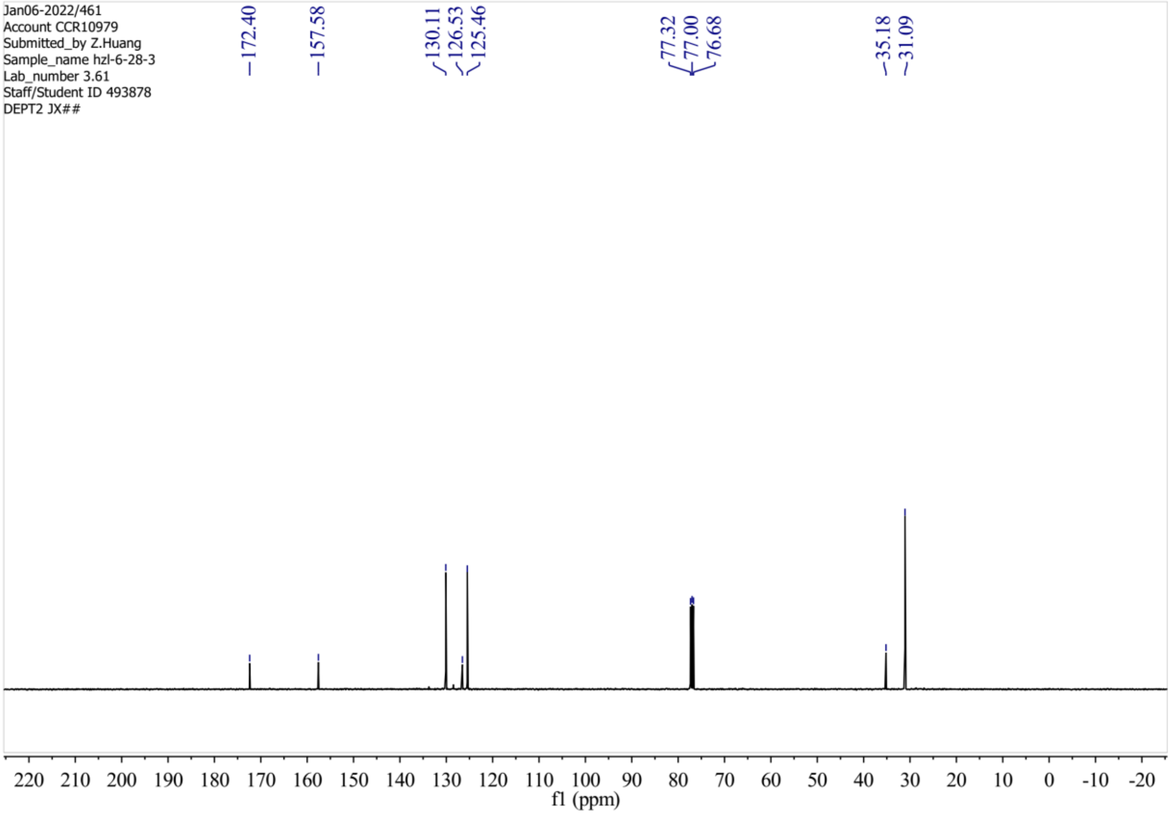

^{13}C NMR

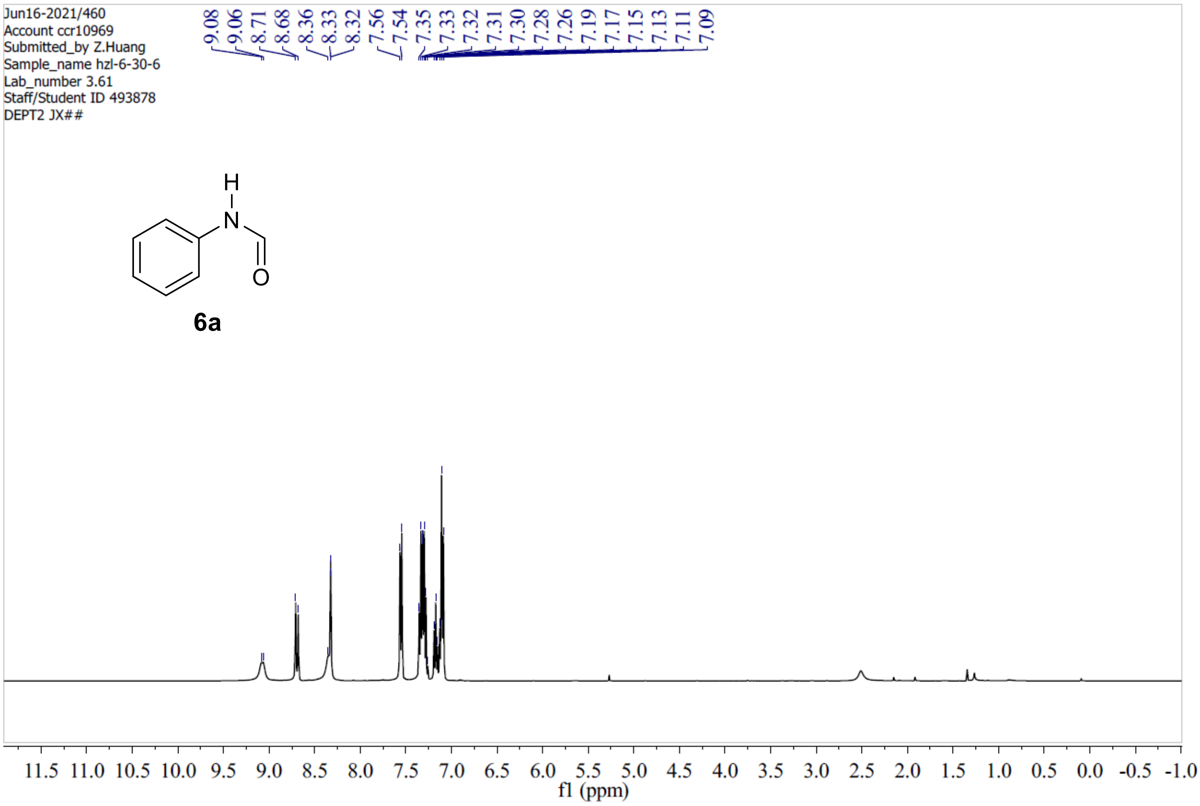

^{1}H NMR

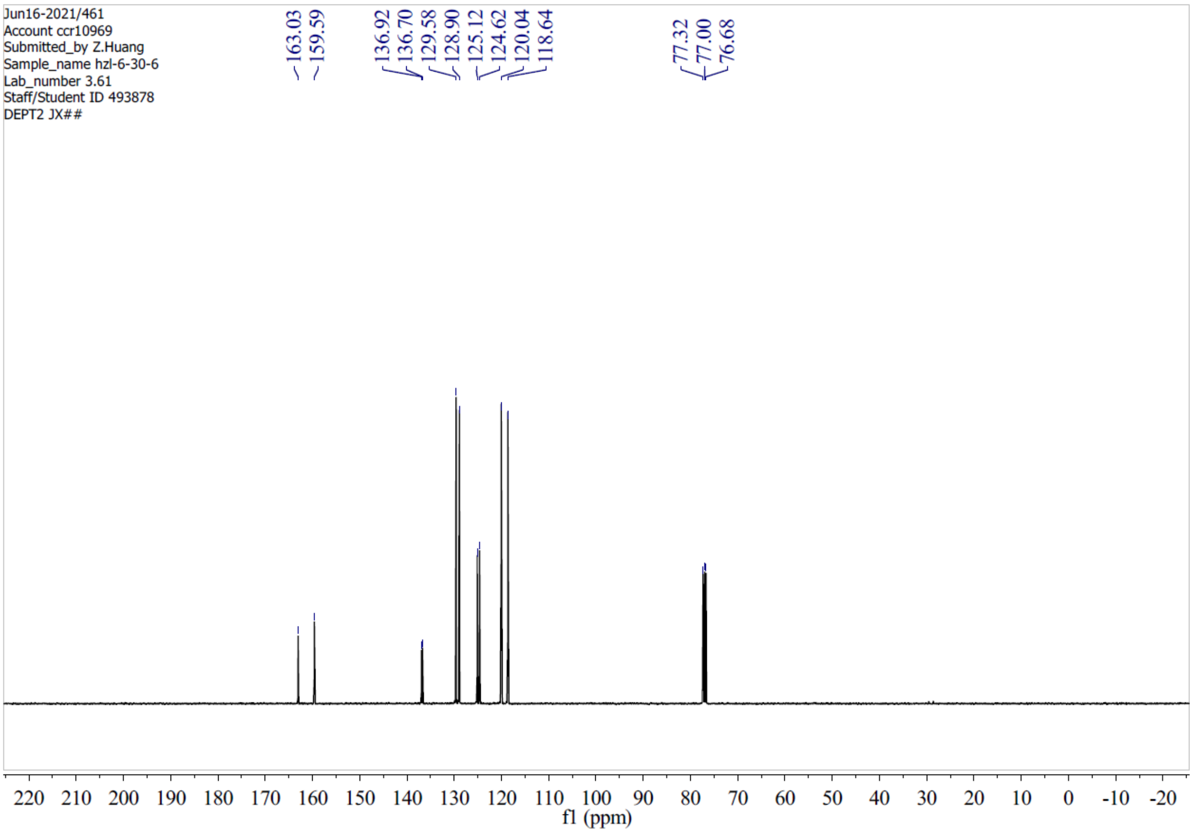

^{13}C NMR

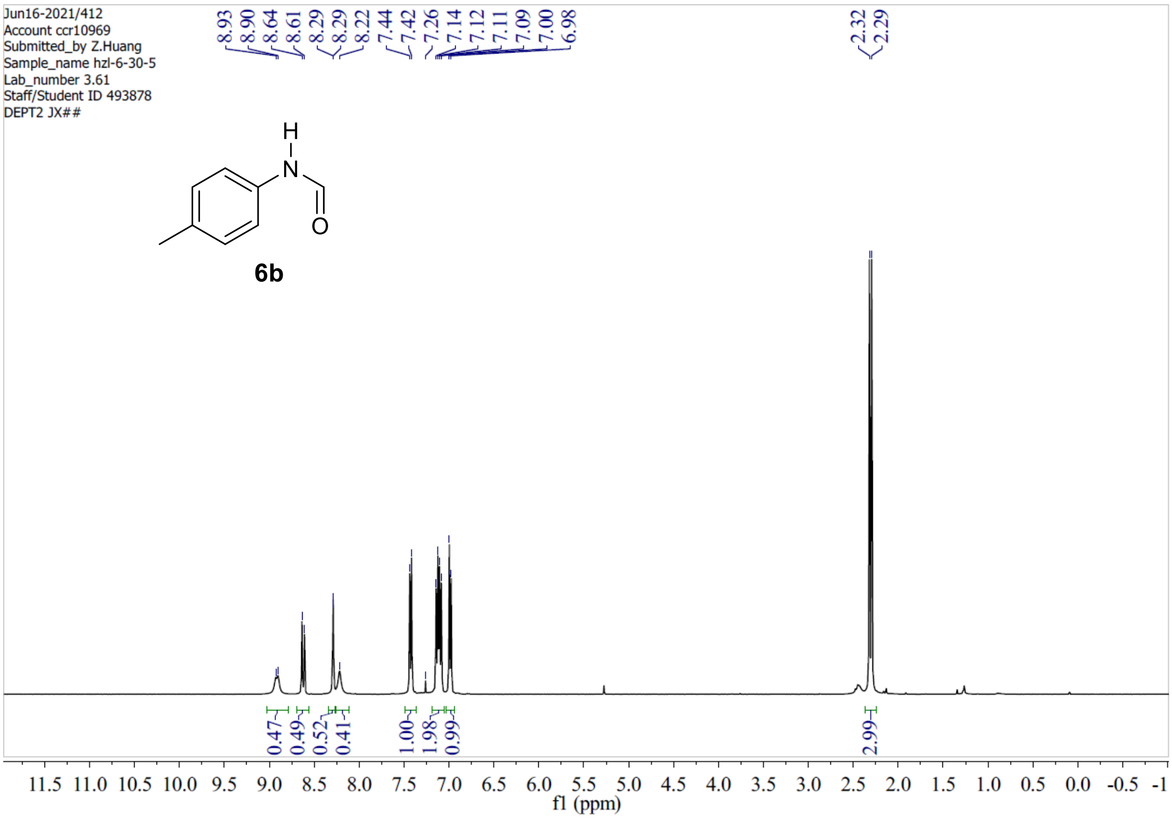

^{1}H NMR

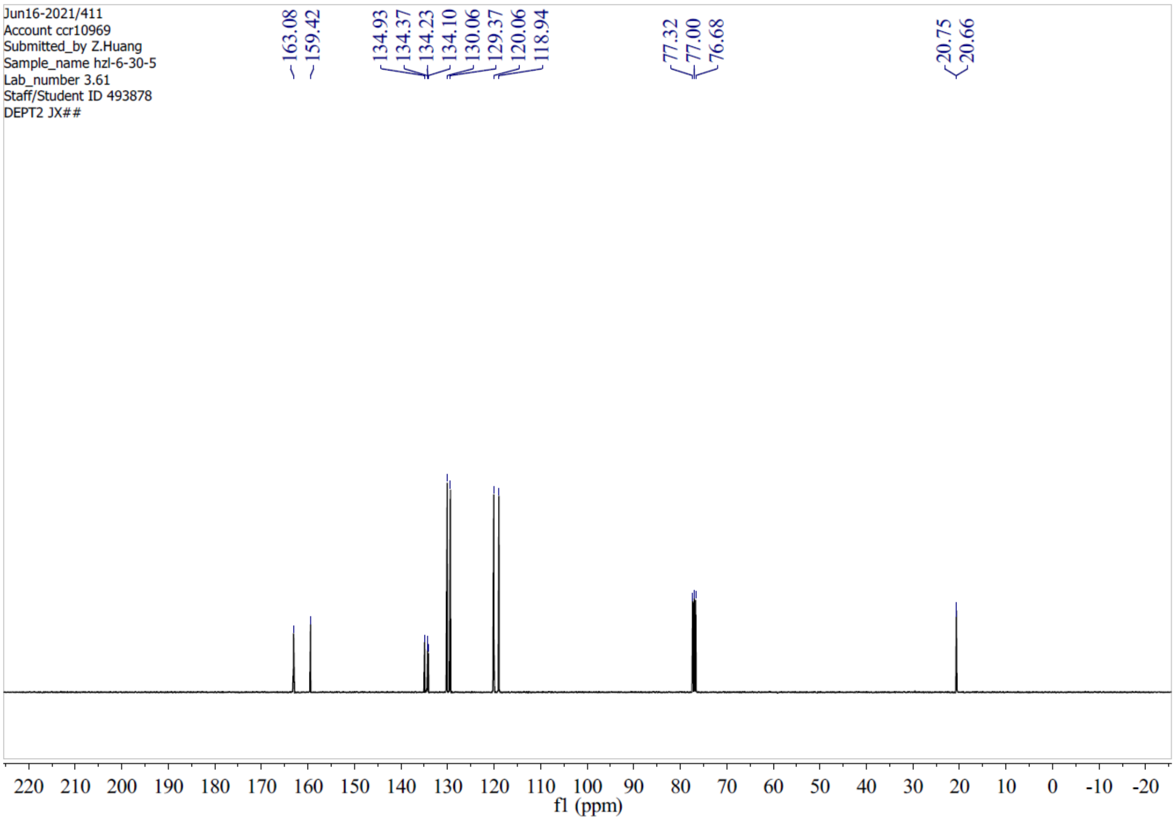

^{13}C NMR

8. References

1. Kropf, H.; Von Wallis, H., 1,2-Dioxolane durch Barton-Typ-Cyclisierung von Alkyl-hydroperoxiden. *Synthesis* **1981,** *1981* (03), 237-240.
2. Cambié, D.; Bottecchia, C.; Straathof, N. J. W.; Hessel, V.; Noël, T., Applications of continuous-flow photochemistry in organic synthesis, material science, and water treatment. *Chem. Rev.* **2016,** *116* (17), 10276-10341.
3. https://www.thorlabs.com/thorproduct.cfm?partnumber=M405L3
4. https://www.thorlabs.com/thorproduct.cfm?partnumber=LEDD1B
5. Stoll, S.; Schweiger, A., EasySpin, a comprehensive software package for spectral simulation and analysis in EPR. *J. Magn. Reson.* **2006,** *178* (1), 42-55.
6. Buettner, G. R., Spin Trapping: ESR parameters of spin adducts 1474 1528V. *Free Rad. Biol. Med.* **1987,** *3* (4), 259-303.
7. Bilski, P.; Reszka, K.; Bilska, M.; Chignell, C. F., Oxidation of the spin trap 5,5-dimethyl-1-pyrroline N-oxide by singlet oxygen in aqueous solution. *J. Am. Chem. Soc.* **1996,** *118* (6), 1330-1338.
8. Amorati, R.; Pedulli, G. F.; Pratt, D. A.; Valgimigli, L., TEMPO reacts with oxygen-centered radicals under acidic conditions. *Chem. Commun.* **2010,** *46* (28), 5139-5141.
9. Berliner, L. J.; Reuben, J., *Spin labeling: theory and applications*. Springer Science & Business Media: 2012; Vol. 8.
10. Frisch, M. J.; Trucks, G. W.; Schlegel, H. B.; Scuseria, G. E.; Robb, M. A.; Cheeseman, J. R.; Scalmani, G.; Barone, V.; Petersson, G. A.; Nakatsuji, H.; Li, X.; Caricato, M.; Marenich, A. V.; Bloino, J.; Janesko, B. G.; Gomperts, R.; Mennucci, B.; Hratchian, H. P.; Ortiz, J. V.; Izmaylov, A. F.; Sonnenberg, J. L.; Williams; Ding, F.; Lipparini, F.; Egidi, F.; Goings, J.; Peng, B.; Petrone, A.; Henderson, T.; Ranasinghe, D.; Zakrzewski, V. G.; Gao, J.; Rega, N.; Zheng, G.; Liang, W.; Hada, M.; Ehara, M.; Toyota, K.; Fukuda, R.; Hasegawa, J.; Ishida, M.; Nakajima, T.; Honda, Y.; Kitao, O.; Nakai, H.; Vreven, T.; Throssell, K.; Montgomery Jr., J. A.; Peralta, J. E.; Ogliaro, F.; Bearpark, M. J.; Heyd, J. J.; Brothers, E. N.; Kudin, K. N.; Staroverov, V. N.; Keith, T. A.; Kobayashi, R.; Normand, J.; Raghavachari, K.; Rendell, A. P.; Burant, J. C.; Iyengar, S. S.; Tomasi, J.; Cossi, M.; Millam, J. M.; Klene, M.; Adamo, C.; Cammi, R.; Ochterski, J. W.; Martin, R. L.; Morokuma, K.; Farkas, O.; Foresman, J. B.; Fox, D. J. *Gaussian 16 Rev. C.01*, Wallingford, CT, 2016.
11. Becke, A. D., Density-functional thermochemistry. III. The role of exact exchange. *J. Chem. Phys.* **1993,** *98* (7), 5648-5652.
12. Lee, C.; Yang, W.; Parr, R. G., Development of the Colle-Salvetti correlation-energy formula into a functional of the electron density. *Phys. Rev. B* **1988,** *37* (2), 785-789.
13. Grimme, S.; Antony, J.; Ehrlich, S.; Krieg, H., A consistent and accurate ab initio parametrization of density functional dispersion correction (DFT-D) for the 94 elements H-Pu. *J. Chem. Phys.* **2010,** *132* (15), 154104.
14. Grimme, S.; Ehrlich, S.; Goerigk, L., Effect of the damping function in dispersion corrected density functional theory. *J. Comput. Chem.* **2011,** *32* (7), 1456-65.
15. Marenich, A. V.; Cramer, C. J.; Truhlar, D. G., Universal solvation model based on solute electron density and on a continuum model of the solvent defined by the bulk dielectric constant and atomic surface tensions. *J. Phys. Chem. B* **2009,** *113* (18), 6378-96.
16. Annen, S.; Zweifel, T.; Ricatto, F.; Grützmacher, H., Catalytic aerobic dehydrogenative coupling of primary alcohols and water to acids promoted by a rhodium(i) amido n-heterocyclic carbene complex. *ChemCatChem* **2010,** *2* (10), 1286-1295.
17. Nemoto, K.; Yoshida, H.; Egusa, N.; Morohashi, N.; Hattori, T., Direct Carboxylation of Arenes and Halobenzenes with CO_2 by the Combined Use of $AlBr_3$ and R_3SiCl. *J. Org. Chem.* **2010,** *75* (22), 7855-7862.
18. Suchý, M.; Elmehriki, A. A. H.; Hudson, R. H. E., A Remarkably simple protocol for the N-formylation of amino acid esters and primary amines. *Org. Lett.* **2011,** *13* (15), 3952-3955.

19. Gim, H. J.; Kang, B.; Jeon, R., Synthesis and biological activity of 5-(4-[2-(Methyl-p-substituted phenylamino)ethoxy]benzyl)thiazolidine-2,4-diones. *Arch. Pharmacal Res.* **2007,** *30* (9), 1055-1061.
